# DeepAnnotation: A novel interpretable deep learning–based genomic selection model that integrates comprehensive functional annotations

**DOI:** 10.1093/gigascience/giaf083

**Published:** 2025-08-28

**Authors:** Wenlong Ma, Weigang Zheng, Shenghua Qin, Chao Wang, Bowen Lei, Yuwen Liu

**Affiliations:** State Key Laboratory of Genome and Multi-omics Technologies, Shenzhen Branch, Guangdong Laboratory for Lingnan Modern Agriculture, Key Laboratory of Livestock and Poultry Multi-Omics of MARA, Agricultural Genomics Institute at Shenzhen, Chinese Academy of Agricultural Sciences, Shenzhen 518124, China; Innovation Group of Pig Genome Design and Breeding, Research Centre for Animal Genome, Agricultural Genomics Institute at Shenzhen, Chinese Academy of Agricultural Sciences, Shenzhen 518124, China; State Key Laboratory of Genome and Multi-omics Technologies, Shenzhen Branch, Guangdong Laboratory for Lingnan Modern Agriculture, Key Laboratory of Livestock and Poultry Multi-Omics of MARA, Agricultural Genomics Institute at Shenzhen, Chinese Academy of Agricultural Sciences, Shenzhen 518124, China; Innovation Group of Pig Genome Design and Breeding, Research Centre for Animal Genome, Agricultural Genomics Institute at Shenzhen, Chinese Academy of Agricultural Sciences, Shenzhen 518124, China; Key Laboratory of Agricultural Animal Genetics, Breeding and Reproduction of Ministry of Education & Key Lab of Swine Genetics and Breeding of Ministry of Agriculture and Rural Affairs, Huazhong Agricultural University, Wuhan 430070, China; State Key Laboratory of Genome and Multi-omics Technologies, Shenzhen Branch, Guangdong Laboratory for Lingnan Modern Agriculture, Key Laboratory of Livestock and Poultry Multi-Omics of MARA, Agricultural Genomics Institute at Shenzhen, Chinese Academy of Agricultural Sciences, Shenzhen 518124, China; Innovation Group of Pig Genome Design and Breeding, Research Centre for Animal Genome, Agricultural Genomics Institute at Shenzhen, Chinese Academy of Agricultural Sciences, Shenzhen 518124, China; State Key Laboratory of Genome and Multi-omics Technologies, Shenzhen Branch, Guangdong Laboratory for Lingnan Modern Agriculture, Key Laboratory of Livestock and Poultry Multi-Omics of MARA, Agricultural Genomics Institute at Shenzhen, Chinese Academy of Agricultural Sciences, Shenzhen 518124, China; Innovation Group of Pig Genome Design and Breeding, Research Centre for Animal Genome, Agricultural Genomics Institute at Shenzhen, Chinese Academy of Agricultural Sciences, Shenzhen 518124, China; State Key Laboratory of Genome and Multi-omics Technologies, Shenzhen Branch, Guangdong Laboratory for Lingnan Modern Agriculture, Key Laboratory of Livestock and Poultry Multi-Omics of MARA, Agricultural Genomics Institute at Shenzhen, Chinese Academy of Agricultural Sciences, Shenzhen 518124, China; Innovation Group of Pig Genome Design and Breeding, Research Centre for Animal Genome, Agricultural Genomics Institute at Shenzhen, Chinese Academy of Agricultural Sciences, Shenzhen 518124, China; State Key Laboratory of Genome and Multi-omics Technologies, Shenzhen Branch, Guangdong Laboratory for Lingnan Modern Agriculture, Key Laboratory of Livestock and Poultry Multi-Omics of MARA, Agricultural Genomics Institute at Shenzhen, Chinese Academy of Agricultural Sciences, Shenzhen 518124, China; Innovation Group of Pig Genome Design and Breeding, Research Centre for Animal Genome, Agricultural Genomics Institute at Shenzhen, Chinese Academy of Agricultural Sciences, Shenzhen 518124, China; Kunpeng Institute of Modern Agriculture at Foshan, Chinese Academy of Agricultural Sciences, Foshan 528226, China

**Keywords:** genomic selection, deep learning, multiomics functional annotations, intermediate molecular phenotypes, interpretability, causal SNPs

## Abstract

**Background:**

Genomic selection, which leverages genomic information to predict the breeding value of individuals, has dramatically accelerated the improvement of economically important traits. The growing availability of multiomics data in agricultural species offers an unprecedented opportunity to enrich this process with prior biological knowledge. However, fully harnessing these rich data sources for accurate phenotype prediction in genomic selection remains in its early stages.

**Results:**

In this study, we present DeepAnnotation, a novel interpretable genomic selection model designed for phenotype prediction by integrating comprehensive multiomics functional annotations using deep learning. To capture the complex information flow from genotype to phenotype, DeepAnnotation aligns multiomics biological annotations with sequential network layers in a deep learning architecture, mirroring the natural regulatory cascade from genotype to intermediate molecular phenotypes—such as *cis*-regulatory elements, genes, and gene modules—and ultimately to phenotypes of economic traits. Comparing against 7 classical models (rrBLUP, LightGBM, KAML, BLUP, BayesR, MBLUP, and BayesRC), DeepAnnotation demonstrated significantly superior prediction accuracy (Pearson correlation coefficient increased by 6.4% to 120.0%) and computational efficiency for 3 pork production traits (lean meat percentage, loin muscle depth, and back fat thickness) using a dataset of 1,700 training Duroc boars and 240 independent validation individuals, each genotyped for 11,633,164 single-nucleotide polymorphisms (SNPs), particularly in identifying top-performing individuals. Furthermore, the interpretability embedded within our framework enables the identification of potential causal SNPs and the exploration of their mediated molecular mechanisms underlying trait variation.

**Conclusions:**

DeepAnnotation is an open-source, interpretable deep learning approach for phenotype prediction, leveraging comprehensive multiomics functional annotations. Freely accessible via GitHub and Docker, it provides a valuable tool for researchers and practitioners in genomic selection.

## Background

Throughout domestication, agricultural species have undergone a deliberate and sustained process of selective breeding aimed at enhancing desirable traits. This elegantly organized practice has led to significant genetic advancements in livestock, poultry, crops, and aquaculture over successive generations [1–4]. In recent years, genomic selection (GS), which uses genomic information to predict economic traits, has substantially revolutionized breeding programs [5]. The core principle of GS, also known as genomic prediction (GP), involves predicting the genomic estimated breeding values (GEBVs) of individuals using phenotype prediction models based on genome-wide DNA markers, genotyped via microarrays or high-throughput next-generation sequencing [6–8]. This shift from traditional phenotype-based selection to genotype-based selection has dramatically accelerated genetic gains by increasing precision and expediting decision-making [9–11]. It has enabled the creation of core germplasm resources and accelerated the breeding programs across various agricultural species [8, 12].

One of the main challenges in applying GS to breeding programs is the “*p* >> *n*” problem, where the number of genotypic markers (*p*) is often much larger than the population size (*n*). For instance, hundreds of thousands, or even millions, of single-nucleotide polymorphisms (SNPs) must be analyzed in relatively small populations [13]. To address this challenge, the best linear unbiased prediction (BLUP) method was proposed, assuming that SNP effects were drawn from a normal distribution with mean zero and constant nonzero variance [14, 15]. BLUP has been widely used for complex trait prediction, significantly advancing biological breeding [5, 16]. Another prominent BLUP-based GS tool is the ridge regression best linear unbiased prediction (rrBLUP) model, which applies ridge regression and is equivalent to BLUP when genetic covariance between lines is proportional to their similarity in genotype space [17, 18]. rrBLUP performs well in phenotype prediction, particularly when the heritability is high, where additive genetic effects dominate and could be well fitted through linear algorithms [13, 19–22]. However, experimental evidence showed that a large proportion of SNPs may have no effects on a specific trait, while others may exhibit small or large effects [23, 24], suggesting that the nonzero SNP effects estimated by the BLUP-based model may overlook important aspects of genetic variance [25]. To address this limitation, the Bayesian-based mixture model called BayesR was introduced, assuming SNP effects follow a mixture of normal distributions, including a point mass at zero [26]. BayesR has proven to be a powerful and flexible tool for genomic prediction, offering insights into the genetic architecture of complex traits [27, 28]. Benefiting from its high scalability, BayesR could be extended to incorporate prior biological information, as demonstrated by the state-of-the-art BayesRC model [29]. In BayesRC, the genome is divided into disjoint categories, each with potentially different proportions of SNP effects drawn from a mixture of normal distributions, significantly improving both genomic prediction accuracy and the power of genomic mapping [30–32]. Moreover, as an extension of the BLUP model, MultiBLUP (hereafter called MBLUP) successfully integrates prior biological information, offering better prediction performance and computational efficiency than alternative methods by utilizing multiple genomic relation matrices (GRMs) [33].

Both BLUP and Bayesian-based models rely on linear algorithms, which may not fully capture the complexity of traits involving nonadditive effects [34, 35]. To better model these complex genetic architectures, machine learning–based approaches have been developed. One notable example is the light gradient boosting machine (LightGBM), which uses a tree-based ensemble algorithm and requires no prior knowledge of genetic effects. LightGBM has outperformed traditional models in certain trait predictions [34]. Other models, such as the kinship-adjusted multiple-loci linear mixed model (KAML), incorporate prior biological knowledge to improve accuracy. KAML enhances prediction efficiency by leveraging weighted kinship information from genome-wide association studies (GWASs) [36].

To capture the complex, nonlinear genetic architecture underlying complex traits and incorporate prior biological information, deep learning (DL) has become one of the most promising methods in genomic selection [37–40]. Recent advances in functional genomics have enabled the integration of multiomics data, enhancing the interpretability of DL models [41–44]. The underlying intuition is that in living organisms, genetic information is transmitted from genotype to final phenotype through multiple levels of intermediate biological processes, including epigenetic modification, transcription, and translation [45]. By structuring DL models to reflect these biological processes, researchers can create biologically meaningful architectures that enhance interpretability. Integrating multiomics data enables backward tracing of DL neurons to identify specific biological entities—such as *cis*-regulatory elements and gene networks—that drive trait formation. This, in turn, helps prioritize potential causal variants and could improve GS performance [46–51]. While DL models integrating multiomics data have been employed in predicting polygenic risk scores of human traits [47, 52–54], their application in agricultural species remains limited, mainly due to the financial and logistical challenges of acquiring multiomics data for individual animals or plants. To address this, models that do not require individual-level multiomics data have been developed [55–57]. However, these studies often rely on preexisting functional annotations, such as GWAS or pathway data. Notably, functional annotation resources for agricultural species are relatively limited compared to those available for humans, highlighting the potential of computational methods to complement functional annotations using existing data. Another obstacle is the lack of a comprehensive understanding of the transmission flow from genetic variants to gene networks. Despite ongoing advancements in experimental technologies, the biological links between causal variants and their target genes remain difficult to validate under strict conditions. Several previous studies have contributed to expanding the comprehensiveness of functional annotations [58–60] and the regulatory networks of hub genes [61, 62].

Here, we introduce DeepAnnotation, a novel, interpretable DL-based genomic selection model that integrates comprehensive species- and tissue-specific functional annotations—without requiring individual-level data—to predict phenotypes. Unlike traditional “black box” DL models, DeepAnnotation aligns multiomics biological annotations with sequential network layers, reflecting the flow of genetic information from DNA. This design offers advantages in both phenotype prediction accuracy and model interpretability. To demonstrate its effectiveness, we applied DeepAnnotation to predict pork production traits, where it outperformed established methods—including rrBLUP, KAML, LightGBM, BLUP, BayesR, MBLUP, and BayesRC—in terms of prediction accuracy, computational efficiency, and top-performing individual selection. Additionally, DeepAnnotation is highly interpretable, providing valuable insights into the potential molecular mechanisms underlying complex traits. DeepAnnotation is freely available via GitHub and Docker, offering a powerful tool for data-driven breeding of complex traits.

## Analyses

### Interpretable deep learning genomic selection model

**Figure 1:**
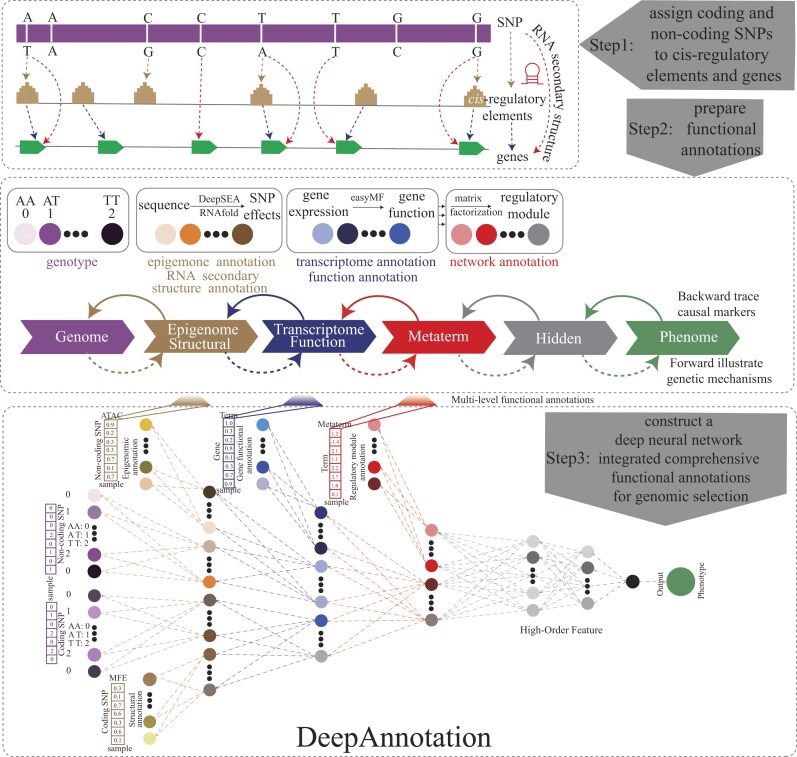
Schematic diagram of DeepAnnotation. The DeepAnnotation pipeline consist of 3 steps: step 1, assign coding and noncoding SNPs to *cis*-regulatory elements and genes; step 2, prepare functional annotations; and step 3, construct a deep neural network that integrates comprehensive functional annotations for genomic selection. For each sample, the first layer receives genotype (a vector of coding and noncoding SNPs) as input. Besides direct feature transfer along with the deep neural network (DNN), DeepAnnotation receives in parallel additional multilevel functional annotations: *cis*-regulatory effects (predicted chromatin accessibility of noncoding SNPs) and RNA secondary structure effects (predicted minimum free energies of coding SNPs) were added in parallel at the second layer, gene function effects (predicted functional scores of genes for each functional term) at the third layer, and regulatory module effects (calculated weights of functional terms for each metaterm) at the fourth layer. Subsequently, 2 hidden layers are used for summarizing the features extracted from the aforementioned layers. Finally, the last layer outputs the predicted phenotypes.

To integrate functional annotations into phenotype prediction, we developed a novel genomic selection model, DeepAnnotation (Fig. 1). This interpretable DL framework incorporates prior biological knowledge, specifically the natural flow of regulatory information from genotype to intermediate molecular phenotypes. Unlike traditional models that rely on low-density microarray data as genotype input, DeepAnnotation utilizes whole-genome sequencing data, which captures the full spectrum of SNPs present within a population. This key distinction enables a comprehensive use of both coding and noncoding functional annotations during model training.

The DeepAnnotation pipeline consists of 3 primary steps. First, we constructed comprehensive functional annotations by assigning *cis*-regulatory elements to their target genes and predicting their potential biological functions. To address gaps in gene function knowledge, we integrated transcriptomic data and applied the easyMF method to enhance and refine these gene function annotations [63]. Through matrix factorization, we identified regulatory module metaterms, which represent groups of genes that cooperate in coordinated biological processes. Second, we employed DeepSEA to predict chromatin accessibility changes resulting from noncoding *cis*-regulatory SNPs [64] and used RNAfold (version 1.8.5) to predict RNA secondary structure alterations induced by coding SNPs [65]. These tools provided valuable insights into the impact of SNPs on regulatory regions as well as transcript and protein abundance. Finally, we integrated functional network annotations and SNP impact predictions into a deep neural network (DNN), forming the DeepAnnotation model. By leveraging functional annotations, DeepAnnotation significantly improves the accuracy and performance of genomic selection. Additionally, its biologically meaningful architecture enables us to trace genetic variations back to their phenotypic consequences, unraveling the complex genetic mechanisms underlying trait variation (Supplementary File 1).

The detailed architecture of the DeepAnnotation model consists of 7 layers, each dedicated to processing distinct types of omics data (Fig. 1 and Supplementary File 1). These layers encompass genotype data, epigenomic features, RNA secondary structure data, transcriptomic profiles, gene function annotations, regulatory module metaterms, and additional high-order features, represented by 2 hidden layers. Beginning with genotype data as the foundational layer, subsequent layers are added to enrich the model’s understanding of intermediate biological processes between genotype and phenotype. These subsequent layers consist of various elements that enhance our understanding of genetic regulation. First, the chromatin accessibility and RNA secondary structure layers provide critical insights into how noncoding and coding SNPs influence gene regulation. Next, the gene function annotation layer elucidates the functional roles of individual genes, while the regulatory module metaterms layer captures higher-order regulatory interactions, identifying coordinated patterns of gene expression across multiple genes and regions. The 2 hidden layers aggregate the information from previous layers, allowing the model to summarize and integrate complex functional annotations. Finally, the top layer predicts the phenotype, consolidating the model’s ability to both predict complex traits and provide biological insights.

To optimize hyperparameters within the constraints of available computational resources, we employed a 2-fold cross-validation (CV) experiment to determine the optimal hyperparameter settings (Methods) (Supplementary Table S1). To evaluate the final prediction performance, we conducted a 5-fold cross-validation using these optimal hyperparameters, ensuring a robust assessment of model accuracy and generalizability (Methods).

### Prediction performance of DeepAnnotation

In preparing functional annotation data for training DeepAnnotation, we utilized RNAfold, DeepSEA, and easyMF models. These tools allowed us to enrich functional annotations beyond genotype, encompassing aspects such as RNA secondary structure, chromatin accessibility, gene function, and gene networks (metaterm regulatory modules). Specifically, the input data for training the DeepAnnotation model were organized into 4 distinct components (Methods):

1. Genotype data: 590,342 potential noncoding *cis*-regulatory SNPs within open chromatin regions and 65,706 coding SNPs.
2. SNP functional impacts: Predicted by DeepSEA for noncoding SNPs and RNAfold for coding SNPs to assess their potential biological effects.
3. Gene annotations: Derived from 4,111 annotated genes located in conserved regions, encompassing 227 Gene Ontology (GO) and Kyoto Encyclopedia of Genes and Genomes (KEGG) terms, all with an area under the curve (AUC) value exceeding 0.9, as determined by easyMF.
4. Metaterm regulatory modules: 31 metaterms extracted from a pool of 27,797 genes, excluding the 4,111 genes from (3), spread across 14,996 terms using easyMF.

To identify the optimal hyperparameter combinations based on these functional annotations, we employed a rigorous evaluation process using Pearson correlation coefficient (PCC) scores between predicted and observed lean meat percentage (LMP) at 100 kg trait measurements within a 2-fold CV framework (Methods). We calculated the mean and median PCCs from a pool of 500 hyperparameter combinations, employing a voting strategy to determine the best hyperparameters. This strategy considered the mean and median PCCs obtained from models utilizing different levels of input annotation:

Level 1 (Genotype): only genotype data.
Level 2 (SNPAnnotation): genotype + SNP functional impact.
Level 3 (Function): genotype + SNP functional impact + gene annotation.
Level 4 (Network): genotype + SNP functional impact + gene annotation + metaterm regulatory module.

**Figure 2:**
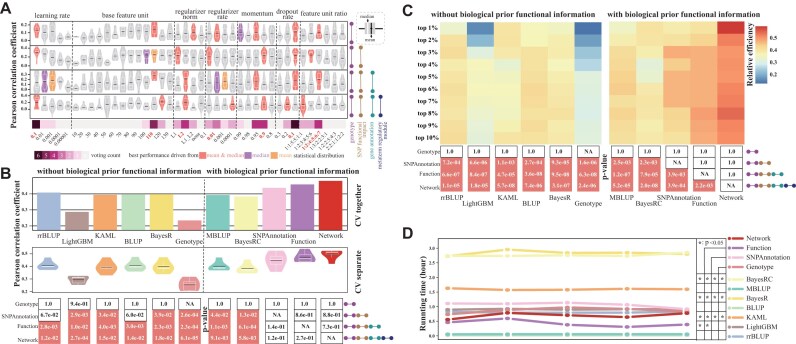
Prediction performance of DeepAnnotation through cross-validation compared with rrBLUP, LightGBM, KAML, BLUP, BayesR, MultiBLUP, BayesRC, and DeepAnnotation on the LMP trait. (A) Pearson correlation coefficient scores of different hyperparameter combinations based on a 2-fold cross-validation (CV) experiment from DeepAnnotation using different types of functional annotation data. (B) PCC evaluation of different models based on 5-fold cross-validation experiment. In the “CV together” approach, each testing fold from the 5-fold cross-validation was consolidated before metric computation. On the other hand, in the “CV separate” approach, metrics were computed separately for each testing fraction of the 5-fold cross-validation. The paired *t* test *P* values of DeepAnnotation compared with other models were displayed on the black boxes, with coral representing significance, with *P* < 0.05. (C) Relative efficiency (RE) values between the predicted and observed phenotypic values of top-ranked samples from the top 1% to the top 10%. The paired *t* test *P* values are displayed in the black boxes, with coral representing significance, with *P* < 0.05. (D) Elapsed training time of different models for 5 CVs. Each column of vertical points represents 1 CV. For DeepAnnotation, the training times do not include the hyperparameter optimization.

For example, the best learning rates based on mean PCC were 0.1, 0.1, 0.001, and 0.1 for levels 1, 2, 3, and 4, respectively, while the best median PCC-based rates were 0.1, 0.1, 0.01, and 0.1 for levels 1, 2, 3, and 4, respectively (Fig. 2A). The voting results indicated a preference for a learning rate of 0.1 with 6 votes for this value, 1 vote for 0.01 and 0.001, and 0 votes for 0.0001 and 0.00001 (Fig. 2A). In cases where multiple parameter values received the same number of votes, we used performance-based selection to determine the most favorable parameter. For instance, both momentum values of 0.9 and 0.95 received 4 votes, but 0.9 yielded a higher average PCC (0.187 vs. 0.139) and was chosen as the optimal value. By employing this strategy, the final optimal hyperparameter settings for DeepAnnotation were as follows: learning rate of 0.1, base feature unit of 110, regularizer norm of L2, regularizer rate of 0.01, momentum of 0.9, dropout rate of 0.1, and a feature unit ratio of 1:2:4:5:6:7 (Fig. 2A and Supplementary Table S1).

We then compared the prediction performance of DeepAnnotation with other established models—rrBLUP, KAML, LightGBM, BLUP, BayesR, MBLUP, and BayesRC—using a rigorous 5-fold CV approach. For models that predict phenotypes directly from genotype without biological prior functional information, the overall PCC scores from all CV results were 0.408 (rrBLUP), 0.288 (LightGBM), 0.393 (KAML), 0.398 (BLUP), 0.398 (BayesR), and 0.234 (Genotype, level 1 for DeepAnnotation with only genotype data) (Fig. 2B). For models incorporating biological prior functional information, the PCC scores were 0.392 (MBLUP), 0.383 (BayesRC), 0.434 (SNPAnnotation, level 2), 0.459 (Function, level 3), and 0.481 (Network, level 4) (Fig. 2B). These results demonstrate that integrating functional annotations significantly improves prediction performance. DeepAnnotation showed a relative improvement of 6.4% to 67.3% over other models, depending on level of functional information used (Fig. 2B). The same conclusion could be reached under the distribution of 5 CV results: averaged PCC scores of 0.408, 0.293, 0.394, 0.405, 0.397, and 0.254 for rrBLUP, LightGBM, KAML, BLUP, BayesR, and Genotype without biological prior functional information. Averaged PCC scores were 0.400, 0.382, 0.447, 0.473, and 0.494 for MBLUP, BayesRC, SNPAnnotation, Function, and Network with biological prior functional information (Fig. 2B). Notably, DeepAnnotation with functional annotations outperformed all other models with statistical significance (*P* < 0.05, paired *t* test based on 5 CV results), except for SNPAnnotation (level 2) when compared to rrBLUP (*P* = 0.067) and BLUP (*P* = 0.06) models.

To further investigate the advantage of DeepAnnotation, we analyzed the relative efficiency (RE) values between predicted and observed phenotypes for top-ranked individuals (top 1% to top 10%) (Fig. 2C). DeepAnnotation consistently outperformed other models in identifying the highest-ranking individuals (paired *t* test, *P* < 0.05) with biological prior functional information. While DeepAnnotation (not accounting for hyperparameter optimization) trained faster than Bayesian-based models (*P* < 0.05), it was slightly slower than BLUP-based models, which are compiled on C frameworks (Fig. 2D). However, the training times for rrBLUP, LightGBM, KAML, and DeepAnnotation were similar, taking only a few hours. Overall, these findings highlight DeepAnnotation’s flexibility and efficiency, incorporating multiomics data to progressively improve phenotype prediction accuracy, particularly in identifying top-ranking candidates.

**Figure 3:**
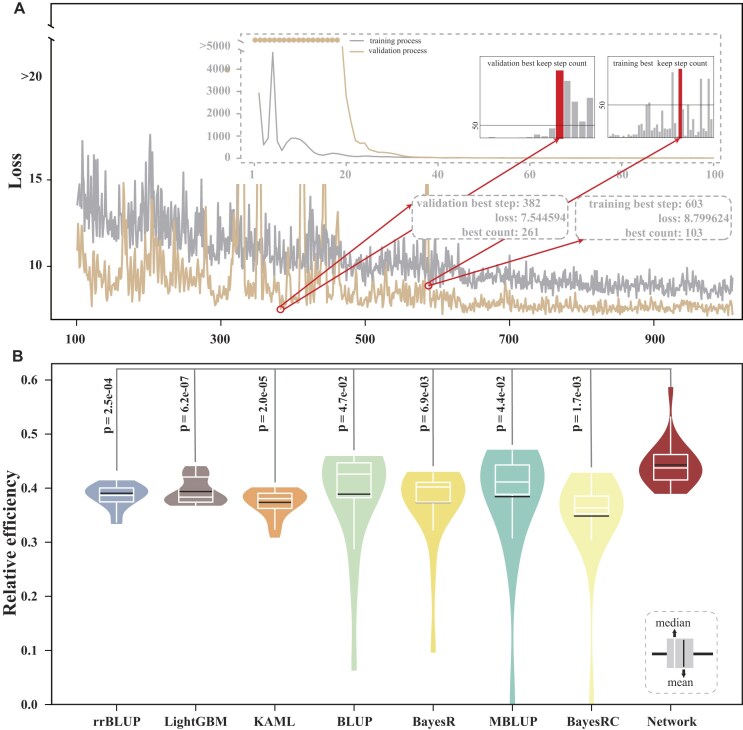
Prediction performance of DeepAnnotation for an independent test dataset of LMP. (**A**) Training and validation status of DeepAnnotation for all functional annotations of each epoch based on the 5-fold cross-validation. (**B**) The distribution of RE scores ofthe top 1∼20 ranked samples under the distribution of 5 CV results.

To assess the robustness of DeepAnnotation, we evaluated its performance on an independent test set of LMP (Fig. 3) (Methods). Using the Network model trained with all functional annotations, we tracked the training losses at each step and then calculated the mean loss of each training process. We determined the optimal training step by counting the number of steps after which the loss was not going down anymore. For example, during optimization, we observed that the minimal training loss remained constant at 8.799624, starting from step 603, and continued for the subsequent 103 steps (Fig. 3A). Therefore, a 5-fold CV experiment was then conducted again with 603 training steps to predict phenotypes, and RE values for top-ranked individuals were averaged to provide a robust performance assessment (Fig. 3B). As anticipated, DeepAnnotation exhibited superior performance compared to other models, as evidenced by its significant (paired *t* test, *P* < 0.05) higher RE scores on the top 1∼20 ranked samples (Fig. 3B), demonstrating its capability in accurately identifying superior individuals. Taken together, these findings highlight the broad utility and great robustness of DeepAnnotation in accurately identifying individuals with exceptional performance.

### Biological interpretability of DeepAnnotation

**Figure 4:**
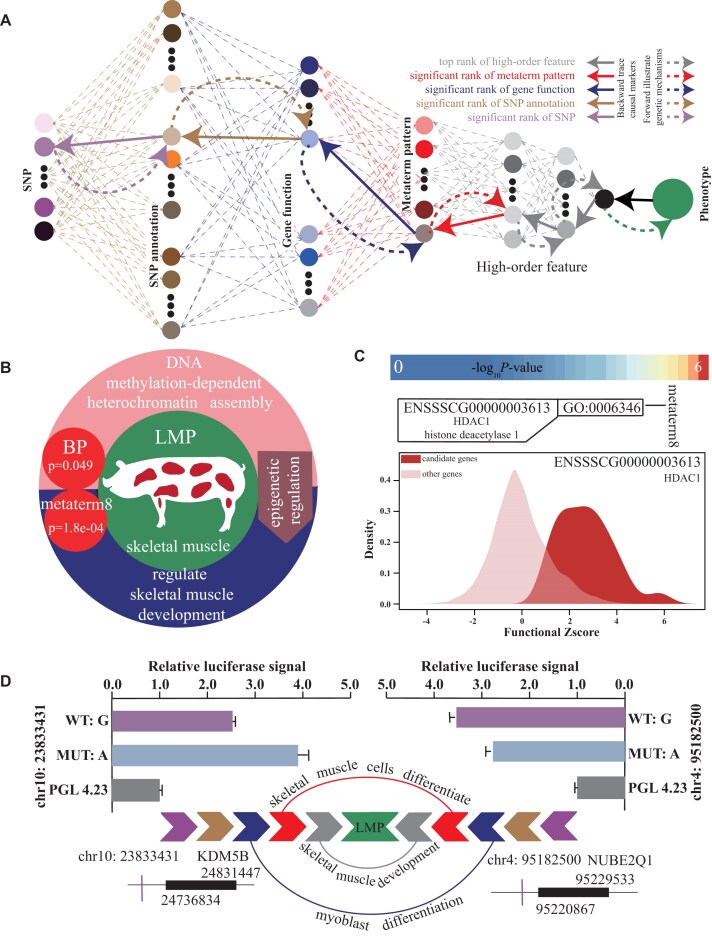
Interpretability of DeepAnnotation allows for the fine-mapping of potential causal SNPs. (A) Schematic diagram of how DeepAnnotation exploited backward tracing to identify potential causal SNPs and their mediated causal genes and gene regulatory modules. (B) Metaterm 8 acts as a coregulatory module that regulates skeletal muscle development through epigenetic regulation. (C) Potential causal genes in metaterm 8 and their functional properties compared with others. (D) DeepAnnotation pinpoints 2 experimentally validated noncoding enhancer-modulating SNPs and proposes how that might influence pork production traits through regulating the expression of genes relevant to skeletal muscle growth and development. The first SNP, chr10:23833431, is predicted to regulate the expression of KDM5B, while the second SNP, chr4:95182500, may influence the expression of NUBE2Q1. Both genes are implicated in myoblast differentiation and function, playing critical roles within the regulatory module governing skeletal muscle cell differentiation. These regulatory effects may ultimately influence skeletal muscle development and contribute to variations in the LMP trait. Supporting evidence from dual-luciferase reporter assays is shown in the bar charts on the left and right.

To demonstrate the biological interpretability of DeepAnnotation, which means providing insights into the genetic underpinnings of LMP traits, we offer a proof-of-concept regarding the model’s interpretability. Focusing on the optimal validation status of the Network model (Fig. 3A), we aim to exploit the proposed backward tracing strategy (Fig. 4A, Supplementary Fig. S1, and Supplementary File 1) by extracting the 5 weights of all nodes trained at training step 382 (Fig. 3A) with all functional annotations through the 5-fold CV experiment and calculating their significance using a meta-strategy with multiple testing correction via the “RobustRankAggreg” R package [66]. A total of 8 metaterms, 4,264 terms, 950 genes, 5,290 *cis*-regulatory elements, 4,804 noncoding SNPs, 567 RNA secondary structures, and 484 coding SNPs showed significant (adjusted *P* < 1.0e-02) contributions to LMP.

Among the significant metaterms, metaterm 8 (adjusted *P* = 1.8e-04) appeared to be acting as a critical coregulatory module regulating skeletal muscle development through epigenetic regulation. The signature functional term for this metaterm was GO:0006346 (adjusted *P* = 0.049) (Fig. 4B and Supplementary Table S2). Previous studies have highlighted the importance of DNA methylation as a key epigenetic modification in skeletal muscle development [67, 68], aligning with the biological process denoted by GO:0006346 (BP domain, DNA methylation-dependent heterochromatin assembly).

Leveraging the comprehensive functional annotation resource and node weights estimated during the training process of DeepAnnotation, we prioritized potential causal markers within metaterm 8. From the pig GO database, only HDAC1 (ENSSSCG00000003613, histone deacetylase 1, *P* = 2.7e-04) was identified as a potential causal gene annotated in GO:0006346. Histone deacetylases (HDACs) play crucial roles in regulating skeletal muscle metabolism, motor adaptation, and exercise capacity [69, 70]. A previous study has shown that HDAC1 is sufficient to activate FoxO and induce skeletal muscle fiber atrophy [70], pointing to its key regulatory role in skeletal muscle development. Further functional annotation using easyMF predicted 49 candidate genes with similar functions to HDAC1, with significant functional similarity (*P* = 9.2e-22) compared to other genes (Fig. 4C and Supplementary Table S3).

Among these 49 genes, 6 genes were identified to significantly (*P* < 0.01) contribute to LMP through a back-tracing strategy (Supplementary Table S3). To identify potential causal SNPs that regulate these 6 genes within ±1-Mb regions, we calculated their significant levels based on the back-tracing strategy. A total of 3 noncoding SNPs were identified as statistically significant (*P* < 0.05) and proposed as *cis*-regulatory variants influencing gene expression related to skeletal muscle development (Supplementary Table S3). However, SNP chr4:95309469 was not predicted to reside within a peak region by the DeepSEA model. The predicted ATAC signals for this variant, with a threshold of 0.147, were 0.117 for the reference allele and 0.106 for the alternative allele, failing to meet the significance criteria. Consequently, the remaining 2 noncoding SNPs were prioritized as potential critical *cis*-regulatory variants with stronger evidence for functional relevance. The first SNP, chr10:23833431 (adjusted *P* = 5.6e-03), is located 903,403 bp upstream of KDM5B (ENSSSCG00000010928, lysine demethylase 5B, adjusted *P* = 3.8e-06), a gene known to play a key role in skeletal muscle differentiation [71]. This SNP was located in an ATAC-seq peak region of skeletal muscle tissue with a predicted score of 0.564 from the DeepSEA model (threshold = 0.147), suggesting its regulatory role in KDM5B expression. Studies have indicated that KDM5B was highly expressed in human skeletal muscle tissue and regulated myoblast differentiation and muscle development [71–73]. Moreover, in a mouse strain with KMD5B knockout, increased phosphorylation of proteins involved in insulin signaling was observed in skeletal muscles, suggesting a role for KDM5B in regulating muscle metabolism [74, 75]. Furthermore, studies of purebred and Duroc-crossbred Iberian pigs, which exhibited significant differences in muscle growth, indicated that KDM5B may regulate gene expression in muscle [76]. These findings suggested a potential role for KDM5B in skeletal muscle development. Additionally, dual-luciferase reporter assays confirmed that chr10:23833431 was located in an enhancer (Student *t* test, *P* < 0.001) (Fig. 4D), and its 2 alleles displayed differential enhancer activity (Student *t* test, *P* = 0.003687) (Fig. 4D).

The second SNP, chr4:95182500 (adjusted *P* = 3.6e-02), was located 38,367 bp upstream of UBE2Q1 (ENSSSCG00000006544, ubiquitin conjugating enzyme E2 Q1, adjusted *P* = 4.9e-04). This SNP resides within an ATAC-seq peak in skeletal muscle tissue with a predicted score of 0.168 from the DeepSEA model (threshold = 0.147), suggesting a potential regulatory role in UBE2Q1 expression. Previous studies have found that UBE2Q1 expression was upregulated in the muscles of male mice with spinal and bulbar muscular atrophy [77], suggesting its involvement in muscle atrophy. Further studies revealed that UBE2Q1 interacts with miR-27a, which inhibits the fast myofiber phenotype [78], suggesting the vital role of UBE2Q1 in skeletal muscle development. Dual-luciferase reporter assays results further validated that chr4:95182500 was located in an enhancer (Student *t* test, *P* < 0.001), with its 2 alleles exhibiting differential enhancer activity (Student *t* test, *P* = 0.0006329) (Fig. 4D).

Taken together, through the back-tracing approach of DeepAnnotation, we proposed 2 potential molecular mechanisms involving noncoding *cis*-regulatory SNPs that may influence pig LMP. This discovery emphasizes the significant potential of DeepAnnotation to elucidate the flow of genetic information from genotype to phenotype by integrating diverse functional annotations (Fig. 4D).

## Discussion

We introduced DeepAnnotation, an innovative framework that predicts phenotypes from genotypes by integrating a broad range of functional annotations. To the best of our knowledge, DeepAnnotation is the first interpretable deep learning approach leveraging comprehensive multiomics functional annotation in livestock. Beyond accelerating the selection of exceptional individuals in genetic improvement, this method also provides insight into the genetic basis underlying critical economic traits. DeepAnnotation is now publicly freely available through the GitHub [79] and Docker [80] repository, extending its capabilities to facilitate big data–driven breeding of complex traits.

With significant advancements in high-density genotyping platforms, the number of genomic loci tested for each individual has surged from tens of thousands to tens of millions of SNPs. Functional annotation of these genomic loci has benefited from the continuous development of high-throughput experimental methods and sophisticated computational algorithms. Together, these technological breakthroughs hold great promise for improving genomic selection prediction accuracy by accurately incorporating biologically functional variants into prediction models. To explore this potential, we conducted an experiment to predict phenotypes using different sets of SNPs (Supplementary Fig. S2) using the rrBLUP model:

1. 11,633,164 SNPs (whole genome) from a published pig population genetics dataset [81]
2. 32,451 SNPs from the GeneSeek porcine 50 K SNP array
3. 296,537 SNPs located in Duroc muscle ATAC-seq peak regions
4. 900,965 SNPs predicted to be located in skeletal muscle open chromatin regions by DeepSEA

The results showed that predicting phenotypes from whole-genome SNPs was slightly more accurate than using only array SNPs (mean PCC: 0.409 vs. 0.405). This suggests that valuable information exists within the whole genome that is not captured by array-based approaches. However, using a more comprehensive set of SNPs also introduces the challenge of incorporating potentially irrelevant information, which could contribute noise during model training. To mitigate this, functional annotations, such as the chromatin accessibility, can be used to prioritize *cis*-regulatory variants. This approach improved prediction performance to a mean PCC of 0.413 (Supplementary Fig. S2). The growing adoption of functional genomics in livestock genetics has led to the incorporation of various omics data into refined databases or web servers, such as FAANG [82], ISwine [46], GWAS Atlas [83], IAnimal [84], and PigBiobank [85]. The integration of such rich data will undoubtedly strengthen genomic selection accuracy for a wide range of farm animals.

However, functional genomics data remain incomplete, and in cases where data such as open chromatin regions are not readily available, computational models like DeepSEA offer an effective strategy to complement these gaps. This is reflected by the increase in prediction PCC to 0.415 when DeepSEA-predicted SNPs were included (Supplementary Fig. S2). Similarly, the incorporation of gene function based on coexpression patterns was instrumental, especially since only 0.017% (2 of 11,941 protein-coding genes annotated with evidence code IDA and TAS) of pig protein-coding genes have robust GO annotations. By using the easyMF model, we were able to complement gene functions, greatly enhancing the construction of multilevel functional annotations within DeepAnnotation.

Ongoing debates persist regarding the performance of genomic selection models, comparing traditional statistical algorithms with newer DL-based algorithms [11, 39, 86]. Furthermore, the relative advantages of employing single genomic data versus multiomics data remain an active topic of discussion [37, 51, 87]. In livestock, the development of complex traits involves intricate biological regulatory processes, where genetic information flows from DNA through intermediate molecular phenotypes before manifesting as economically relevant traits [88]. Capturing this nonlinear information flow through intermediate molecular phenotypes can potentially outperform linear statistical models that rely solely on genotype data [51]. Our findings align with this, as the prediction performance of DeepAnnotation gradually improved, significantly outperforming other genomic selection models (Fig. 2B, C). Notably, DeepAnnotation demonstrated superior performance in selecting top-ranked individuals (top 1% and top 10%) (Fig. 3B, C), highlighting the advantage of integrating deep learning with multiomics data. This integration enables the capture of intricate genetic interactions that might be overlooked by relying solely on single omics (genotype) and linear models.

**Figure 5:**
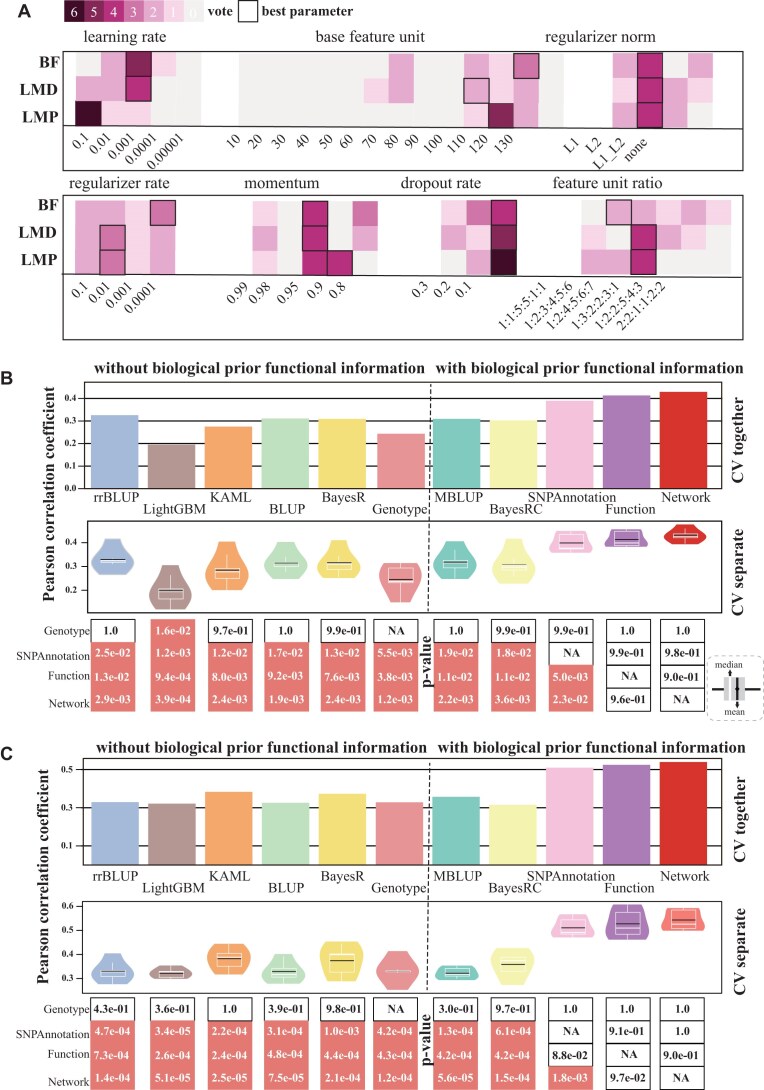
Prediction performance of DeepAnnotation in terms of robustness. (A) Optimized hyperparameters of DeepAnnotation on LMP, LMD, and BF traits. PCC evaluation of different models based on 5-fold cross-validation experiment for (B) LMD and (C) BF. In the “CV together” approach, each testing fold from the 5-fold cross-validation was consolidated before metric computation. On the other hand, in the “CV separate” approach, metrics were computed separately for each testing fraction of the 5-fold cross-validation. The paired *t* test *P* values of DeepAnnotation compared with other models are displayed in the black boxes, with coral representing significance with *P* < 0.05.

To further validate the superior performance of DeepAnnotation, we optimized hyperparameters for 2 additional pork production traits: loin muscle depth (LMD) at 100 kg and back fat thickness (BF) at 100 kg (Fig. 5A and Supplementary Table S1). Our analyses consistently showed the advantage of DeepAnnotation in prediction accuracy compared with other models (Fig. 5B, C). The relative improvement in prediction accuracy was substantial, ranging from 19.5% to 120.0% for LMD (Fig. 5B) and 33.1% to 71.0% for BF (Fig. 5C) based on the overall PCC scores when taking all CV results together, depending on the level of functional information used. These results underscore the importance of considering different combinations of hyperparameters for optimizing performance in diverse scenarios.

Beyond the superior prediction performance, we also focused on the biological interpretability of the DeepAnnotation model, a crucial aspect in deep learning application [34, 41]. Unlike the traditional “black box” nature of neural networks, DeepAnnotation’s layers were designed with biologically interpretable identities to mimic the flow of genetic information (Fig. 1). This architecture facilitates the identification of trait-relevant SNPs, genes, and pathways through back-propagation from the phenotype layer to the genotype layer, enabling the proposal of potential molecular mechanisms influencing traits. This capability was demonstrated by fine-mapping 2potential causal noncoding SNPs implicated in LMP formation, whose *cis*-regulatory functions were experimentally validated (Fig. 4D), further underscoring the model’s interpretability.

**Figure 6:**
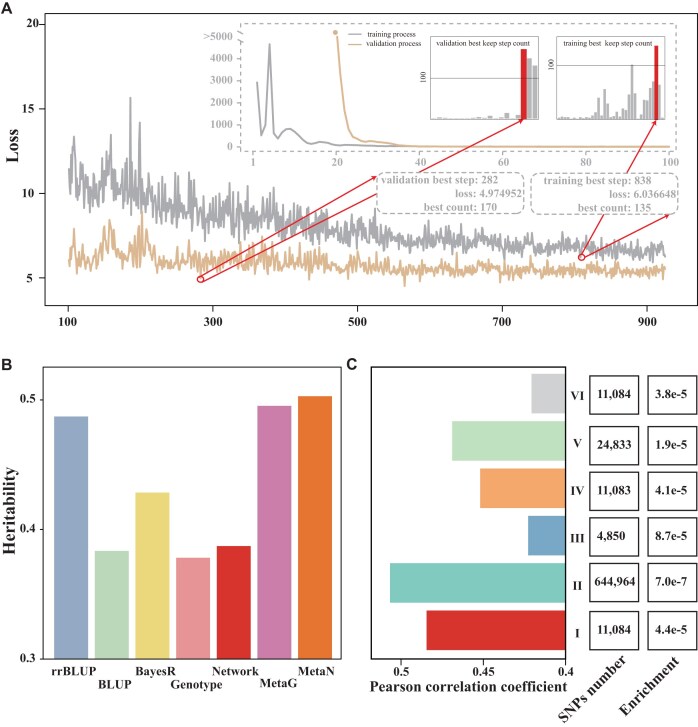
Evaluation of statistically significant variants. (A) Training and validation performance of the Genotype model across epochs, based on 5-fold cross-validation. (B) Estimated heritability of significant SNPs (*P* < 0.05) identified by different models for the LMP trait. (C) Genomic prediction accuracy (measured by PCC using the rrBLUP model), SNP number, and enrichment of informative SNPs across different SNP sets: (I) 11,084 potential causal variants identified by DeepAnnotation (including 10,041 noncoding variants or *cis*-regulatory elements and 1,043 coding or RNA secondary structure-related variants with *P* < 0.01 from the backtracking strategy). (II) 644,964 SNPs located within 1 Mb of the 11,084 DeepAnnotation-predicted causal variants. (III) 4,850 SNPs with GWAS *P* < 1.0e-6. (IV) 11,083 SNPs with GWAS *P* < 1.0e-5. (V) 24,833 SNPs with GWAS *P* < 1.0e-4. (VI) Ten sets of 11,084 SNPs randomly selected across the genome (mean PCC reported).

Identifying causal variants remains a significant challenge. To further evaluate the statistically significant variants identified by DeepAnnotation, we estimated both the heritability and genomic prediction accuracy for the real LMP trait. We compared the Network and Genotype models, representing DeepAnnotation with and without functional annotations, respectively. During 5-fold cross-validation on 1,700 training samples, validation losses were monitored to determine the optimal training epoch, which occurred at epoch 282 for the Genotype model (Fig. 6A). For comparison, we selected rrBLUP, BLUP, and BayesR as baseline models based on their balance between performance and runtime (Fig. 2B, D). The heritability explained by statistically significant SNPs (*P* < 0.05) was 0.378, 0.383, 0.387, 0.428, 0.487, 0.495, and 0.503 for Genotype, BLUP, Network, BayesR, rrBLUP, metaG (a meta-analysis combining rrBLUP, BLUP, BayesR, and Genotype), and metaN (combining Network with other models), respectively (Fig. 6B). While DeepAnnotation alone performed slightly worse than the baseline models, its integration achieved higher heritability, with metaN performing the best, highlighting the complementarity between DeepAnnotation and BLUP- or Bayesian-based approaches, as well as emphasizing the importance of accurate functional annotations. Moreover, the 11,084 potential causal variants (set I) identified by DeepAnnotation demonstrated significantly higher GWAS power compared to the remaining SNPs (*t* test, *P* = 3.89e-4). In terms of genomic prediction accuracy (measured by PCC) (Fig. 6C), DeepAnnotation prioritized SNPs (with *P* < 0.01 from the backtracking strategy), outperforming those selected solely from GWAS at stringent thresholds (*P* < 1.0e-6 [set III], 1.0e-5 [set IV], 1.0e-4 [set V]), as well as randomly selected SNPs (set VI). Although the SNPs located within 1 Mb (set II) of the DeepAnnotation-identified variants achieved the highest prediction accuracy, DeepAnnotation demonstrated greater enrichment of informative SNPs—calculated as the ratio of prediction accuracy (PCC) to SNP set size—than set II, set IV, set V, and set VI, but slightly less than the highly stringent GWAS set III (Fig. 6C). These findings indicate that, although the current backward tracing strategy (Fig. 4A, Supplementary Fig. S1, and Supplementary File 1) may miss certain key loci, DeepAnnotation achieved a higher enrichment of informative SNPs than standard SNP selection strategies, with the exception of the most stringent GWAS threshold, underscoring the potential of DeepAnnotation to uncover novel genetic insights. Future work should focus on improving the backward tracing strategy to identify additional informative SNPs, genes, and gene modules, thereby further elucidating the genetic architecture of LMP and other economically important traits.

Despite the superior performance and the broad application of DeepAnnotation, several limitations in current study warrant consideration. First, the dataset used for training (1,700 samples) and independent validation (240 samples) in this study, while informative, is relatively small for deep learning applications. Deep learning models typically require large-scale datasets to achieve robust training and validation, particularly when the number of SNPs far exceeds the number of samples. The limited sample size in this study may hinder the accurate estimation of molecular marker effects and the generalizability of the model, as well as causal variant identification. Future work should explore the performance of DeepAnnotation using significantly larger training and external validation datasets to better assess its scalability and robustness, as well as the performance of causal variant fine-mapping. Second, the influence of linkage disequilibrium (LD) was not accounted for when identifying potential causal SNPs. In this study, potential causal markers were detected through a back-tracing strategy combined with a meta-strategy and multiple testing correlation, without further adjustments for LD. Although dual-luciferase reporter assays provided experimental evidence supporting the *cis*-regulatory function of the identified variants, this approach alone is insufficient to fully validate causality. Future studies should incorporate LD adjustments and employ direct gene knockout experiments to further investigate the functional impact of the identified SNPs and confirm their causal roles.

### Potential implications

We introduce DeepAnnotation, a novel genomic selection approach, which is publicly available through GitHub and Docker image. This framework leverages comprehensive multiomics functional annotations to predict phenotypes. Comparative evaluations against 7 well-established models—namely, rrBLUP, LightGBM, KAML, BLUP, BayesR, MBLUP, and BayesRC—highlighted DeepAnnotation’s superior performance with computational efficiency. The interpretable architecture of DeepAnnotation addresses the typical “black box” nature of deep learning models. By providing insight into the underlying genetic mechanisms, it facilitates the fine-mapping of potential causal SNPs and uncovers crucial information about the genetic basis of complex traits. This interpretability is particularly valuable for understanding the biological processes that contribute to economically important traits in livestock. It can aid in the identification of key genetic markers and regulatory mechanisms, accelerating the development of breeding strategies and improving the efficiency of livestock breeding programs.

## Methods

### Genomic selection models

#### BLUP model

Given the genotype matrix ${\boldsymbol{Z}}$(*n*  $ \times $  *p; n* individuals and *p* markers) and the corresponding vector of phenotype values ${\boldsymbol{y}}( {n \times 1} )$, the general mathematical statistical model for BLUP can be defined as the following standard linear regression formula:


\begin{eqnarray*}
{\boldsymbol{y}} = {\boldsymbol{\mu }} + {\boldsymbol{Zg}} + {\boldsymbol{\varepsilon }}
\end{eqnarray*}

where ${\boldsymbol{\mu }}$ represents the vector of overall mean observed phenotype values of $n$ individuals, ${\boldsymbol{Z}}$ is a matrix of SNP genotypes (e.g., [aa, Aa, AA] = [0, 1, 2] for biallelic SNPs), ${\boldsymbol{g}}\sim N( {0,\ {\boldsymbol{K}}\sigma _g^2} ){\boldsymbol{\ }}$ represents the vector of SNP effects, and ${\boldsymbol{\varepsilon }}\sim N( {0,I\sigma _\varepsilon ^2} )\ $ represents the vector of random residual effects. Here, $\sigma _\varepsilon ^2$ is the variance of residual effects, $\sigma _g^2$ is the variance of genetic effects, ${\boldsymbol{I}}$ is an identity matrix with *n* rows and *n* columns, ${\boldsymbol{K}}$ is a genomic similarity matrix (GSM) specifying the correlation structure of ${\boldsymbol{g}}$ and could be calculated by ${\boldsymbol{K}} = {\boldsymbol{ZZ^{\prime}}}/p$ with $p$ representing the number of SNPs, and ${\boldsymbol{Z^{\prime}}}$ represents the transpose of ${\boldsymbol{Z}}$. Finally, we implemented the BLUP model by setting “–reml-pred-rand –reml-est-fix –blup-snp” with GCTA software [89].

### rrBLUP model

The rrBLUP model is equivalent to BLUP in the context of mixed models [90], which could be defined by the following formula:


\begin{eqnarray*}
{\boldsymbol{y}} = {\boldsymbol{WGu}} + {\boldsymbol{\varepsilon }}
\end{eqnarray*}

where ${\boldsymbol{u}}\sim N( {0,{\boldsymbol{I}}\sigma _u^2} )$ represents the SNP effects vector, ${\boldsymbol{G}}$ represents the genotype matrix, ${\boldsymbol{W}}$ represents the designed matrix, ${\boldsymbol{\varepsilon }}$ represents the random residual effects, and ${\mathrm{\lambda }} = {\mathrm{\sigma }}_e^2/{\mathrm{\sigma }}_{\mathrm{u}}^2$ represents the ridge parameter. In contrast to ordinary regression, the number of markers cannot exceed the number of observations. Finally, we implemented the rrBLUP model with “mixed.solve” function in R package “rrBLUP” [18].

### MultiBLUP model

The basic statistical mathematical model underlying MultiBLUP is the same as BLUP, except for incorporating multiple random effects ${\boldsymbol{g}} = [ {{{\boldsymbol{g}}^1},{{\boldsymbol{g}}^2},\ldots,{{\boldsymbol{g}}^m}} ]$ for different classes of SNPs [33]. The predefined class ${\mathrm{c}}\,\in\,[ {{{\mathrm{c}}_1},{{\mathrm{c}}_2},\ldots,{{\mathrm{c}}_{\mathrm{m}}}} ]$ could be identified by the prior biological information. MultiBLUP extends the BLUP model to include the genomic relation matrix specified by ${\boldsymbol{K}} = [ {{{\boldsymbol{K}}^1},{{\boldsymbol{K}}^2},\ldots,{{\boldsymbol{K}}^m}} ]$ and the corresponding variances ${\mathrm{\sigma }} = [ {{\mathrm{\sigma }}_1^2,{\mathrm{\sigma }}_2^2,\ldots,{\mathrm{\sigma }}_{\mathrm{m}}^2} ]$:


\begin{eqnarray*}
{\boldsymbol{y}} = \ \mathop \sum \nolimits_{i = 1}^m \mathop \sum \nolimits_{j = 1}^{{R_i}} {\boldsymbol{Z}}_j^i{\boldsymbol{g}}_j^i + \ {\boldsymbol{\varepsilon }}
\end{eqnarray*}

where each ${{\boldsymbol{K}}^m} = {{\boldsymbol{Z}}^{\boldsymbol{m}}}{{\boldsymbol{Z}}^{\boldsymbol{m}}}^{\boldsymbol{^{\prime}}}/{p_m}\ $ is the modified form of a genotype matrix ${{\boldsymbol{Z}}^{\boldsymbol{m}}}$ corresponding to a class set of SNPs ${R_i}$ of size ${p_m}$, $g_j^i\sim N( {0,\sigma _i^2/{p_i}} )$. The estimation of variance parameters ${\mathrm{\sigma }}_1^2,\ldots,{\mathrm{\sigma }}_{\mathrm{m}}^2$ and ${\mathrm{\sigma }}_{\mathrm{\varepsilon }}^2$ could be achieved by maximizing the log-likelihood:


\begin{eqnarray*}
- \frac{n}{2}\log \left( {2\pi } \right) - \frac{1}{2}{\boldsymbol{y^{\prime}vy}} - \frac{1}{2}{\mathrm{log}}\left| {\boldsymbol{v}} \right|
\end{eqnarray*}

Here, ${\boldsymbol{v}} = \sigma _1^2{{\boldsymbol{K}}^1} + \ldots + \sigma _1^2{{\boldsymbol{K}}^m} + \sigma _\varepsilon ^2{\boldsymbol{I}}$. Finally, we implemented the MultiBLUP model by setting “–autosome –make-grm-alg 1 –make-grm –reml –mgrm –reml-pred-rand –reml-est-fix –blup-snp ” with GCTA software [89].

### Hierarchical Bayesian mixture (BayesR) model

BayesR is a Bayesian-based method for deriving the prediction equation that assumes SNP effects follow a series of normal distributions. The general mathematical statistical model for BayesR can be defined by the following formula:


\begin{eqnarray*}
{\boldsymbol{y}} = \mu + {\boldsymbol{X\beta }} + {\boldsymbol{e}}
\end{eqnarray*}

where $\mu $ is the intercept that represents the general mean, ${\boldsymbol{X}}( {n,p} )$ represents the numerical genotype matrix, ${\boldsymbol{\beta }}( {p,1} )$ represents the vector of SNP effects, and ${\boldsymbol{e}}( {n,1} )$ represents the vector of residuals with $e\sim N( {0,{\boldsymbol{I}}\sigma _e^2} )$. Briefly, the SNP effects are modeled by a mixture of 4 normal distributions with zero mean, and the variances are fixed specified by:


\begin{eqnarray*}
p\left( {{\beta _j}{\mathrm{|}}\pi ,\sigma _g^2} \right) &=& {\pi _1} \times N\left( {0,0 \times \sigma _g^2} \right) + {\pi _2} \times N\left( {0,{{10}^{ - 4}} \times \sigma _g^2} \right)\\
&& + {\pi _3} \times N\left( {0,{{10}^{ - 3}} \times \sigma _g^2} \right) + {\pi _4} \times N\left( {0,{{10}^{ - 2}} \times \sigma _g^2} \right)
\end{eqnarray*}

Here, $\sigma _g^2$ represents the total additive genetic variance, and the mixing proportions ${\boldsymbol{\pi }} = [ {{\pi _1},{\pi _2},{\pi _3},{\pi _4}} ]$ are drawn from a Dirichlet distribution with parameter $ = ( {1,1,1,1} )$. The constant allocation values $( {0,{{10}^{ - 4}},{{10}^{ - 3}},{{10}^{ - 2}}} )$ imply the SNPs are assigned to 4 different effect size classes: null, small, medium, and large, corresponding respectively to 0%, 0.01%, 0.1%, and 1% of $\sigma _g^2$. Finally, we implemented BayesR by setting “-burnin 5000 -numit 10000 -seed 0” with “bayesR” software [27].

### Bayesian genomic prediction with disjoint annotations (BayesRC) model

The central statistical linear model of BayesRC is the same as BayesR; the main difference between them is that BayesRC incorporates independent biological prior information to allocate each SNP to a specific class ${\boldsymbol{C}}$, given the constraint condition that $| {{{\boldsymbol{C}}_j}} | = 1$ for the *j*th SNP [29]. The SNP effects for each class $c\,\in\,[ {{c_1},{c_2},\ldots,{c_m}} ]$ are defined by the following formula:


\begin{eqnarray*}
p\left( {{\beta _j}{\mathrm{|}}\pi ,\sigma _g^2,{{\boldsymbol{C}}_j} = c} \right) &=& {\pi _{1,c}} \times N\left( {0,0 \times \sigma _g^2} \right) + {\pi _{2,c}}\\
&& \times N\left( {0,{{10}^{ - 4}} \times \sigma _g^2} \right) + {\pi _{3,c}} \times N\left( {0,{{10}^{ - 3}} \times \sigma _g^2} \right)\\
&& + {\pi _{4,c}} \times N\left( {0,{{10}^{ - 2}} \times \sigma _g^2} \right)
\end{eqnarray*}

where $\mathop \sum \limits_{k = 1}^4 {\pi _{k,c}} = 1$ for all $c$ with $m$ representing the number of independent classes. Here, ${\pi _c}$ also is drawn from a Dirichlet distribution with parameter $ = ( {1,1,1,1} )$. Finally, we implemented BayesR by setting “-burnin 5000 -numit 10000 -seed 0 -ncat 5” with “bayesRCO” software [32].

### Light gradient boosting machine (LightGBM) model

LightGBM is an ensemble model of gradient boosting decision trees (GBDT) [34]. For phenotype prediction from the genotype matrix, GBDT uses decision trees to learn a function from the input space of numerical genotype matrix ${\mathcal{X}^p}$ to the gradient space $\mathcal{H}$ [91]. For GBDT, for training dataset $O$ on a fixed node of the decision tree, the information variance gain of splitting feature $j$ at point $d$ for this node is defined by the following formula:


\begin{eqnarray*}
{V_{j|O}}\left( d \right) = \frac{1}{{{n_O}}}\left( {\frac{{{{\left( {\mathop \sum \nolimits_{{x_i} \in\, O:{x_{ij}} \le d} {h_i}} \right)}^2}}}{{n_{l|O}^j\left( d \right)}} + \frac{{{{\left( {\mathop \sum \nolimits_{{x_i} \in\, O:{x_{ij}} > d} {h_i}} \right)}^2}}}{{n_{r|O}^j\left( d \right)}}} \right)
\end{eqnarray*}

where ${n_O} = \sum I| {{x_i}\,\in\,O} |,{\mathrm{\ }}n_{l|O}^j( d ) = \sum I| {{x_i}\,\in\,O:{x_{ij}} \le d} |{\mathrm{\ }}and{\mathrm{\ }}n_{r|O}^j( d ) = \sum I| {{x_i}\,\in\,O:{x_{ij}} > d} |$, ${h_i}$ represent the negative gradients of the loss function with respect to the output of the model. Then the decision tree algorithm selects $d_j^{\mathrm{*}} = \textit{argma}{x_d}{V_j}( d )$ and calculates the largest gain ${V_j}( {d_j^{\mathrm{*}}} )$. Lastly, the data are split according to feature ${j^{\mathrm{*}}}$ at point ${d_{j{\mathrm{*}}}}$ into the left and right child nodes. Finally, we implemented the LightGBM model with the “lgb.train” function by setting “objective = regression, metric = l2, num_threads = 5, learning_rate= 0.1” in R package “lightgbm” [92].

### KAML linear mixed model

KAML is a flexible model that extends a linear mixed model by integrating pseudo quantitative trait nucleotides (pseudo-QTNs) as covariates and an optimized trait-specific random effect [36]. The general mathematical statistical model for KAML can be defined by the following formula:


\begin{eqnarray*}
{\boldsymbol{y}} = {\boldsymbol{X}}b + {\boldsymbol{Q}}q + {\boldsymbol{Z}}{\beta ^*} + {\boldsymbol{\varepsilon }}
\end{eqnarray*}

where $b$ is a vector of the fixed covariate effects with the corresponding coefficient matrix ${\boldsymbol{X}}$; ${\boldsymbol{Q}} = [ {{Q_1},{Q_2},\ldots,{Q_k}} ]$ represents the $k$ covariates that are derived from a multiple regression model-based selection procedure; ${\beta ^*}\sim N( {0,{{\boldsymbol{K}}_{\boldsymbol{w}}}\sigma _g^2} )$ is a vector of random effects representing the individual genetic values; $\varepsilon \sim N( {0,{\boldsymbol{I}}\sigma _e^2} )$ is a vector of residual effects; $\sigma _g^2$ and $\sigma _e^2$ are the genetic variance and residual variance, respectively; and ${{\boldsymbol{K}}_{\boldsymbol{w}}}$ is a SNP-weighted kinship (genomic relation matrix) that can be formulated as follows:


\begin{eqnarray*}
{K_{wij}} = \frac{1}{m}\mathop \sum \nolimits_{k = 1}^m \frac{{\left( {{M_{ik}} - 2{p_k}} \right){\xi _k}\left( {{M_{jk}} - 2{p_k}} \right)}}{{2{p_k}\left( {1 - {p_k}} \right)}}
\end{eqnarray*}

Here, ${\xi _k}$ is the weight of the *k*th SNP, $m$ is the number of SNPs, ${M_{ik}}$ is the numeric value of the genotype matrix of the *k*th SNP in the *i*th individual, and $p$ is the frequency of the coded allele. The weight ${\xi _k}$ can be derived from the following formula:


\begin{eqnarray*}
{\xi _k}|\left( {\alpha ,\gamma } \right)\sim \left\{ {\begin{array}{@{}*{1}{c}@{}} {\ 1;\ 1 - \gamma \ }\\ {1 + lo{g_\alpha }{P_{m\gamma }} - lo{g_\alpha }{P_k};\ \gamma } \end{array}} \right.
\end{eqnarray*}

Here, $P$ represents the ordered *P* values of all SNPs from the GWAS result, $\alpha $ is the base value of logarithmic function, and $\gamma $ is the percentage of top significant SNPs to be weighted. Finally, we implemented the KAML model by setting “bin.size=1000000,max.nQTN=TRUE,sample.num=2, crv.num=5, cpu=5” in R package “kaml.”

### Deep learning–based genomic selection model with comprehensive functional annotations (DeepAnnotation)

The DeepAnnotation model is a deep learning–based approach for genomic selection, which integrates multiple types of prior biological knowledge derived from various omics data. In this study, we assigned 7 layers to represent different types of prior knowledge. The basic mathematical framework for each layer is defined as follows:


\begin{eqnarray*}
{\boldsymbol{y}} = f\left( {{\boldsymbol{wx}} + {\boldsymbol{b}}} \right)
\end{eqnarray*}

where ${\boldsymbol{y}}$ represents a vector of the output values of each layer, $f( \cdot )$ represents the active function, ${\boldsymbol{w}}$ represents the weights of markers, ${\boldsymbol{x}}$ represents the vector of input values, and ${\boldsymbol{b}}$ represents the bias. The overall loss function was defined by the following formula:


\begin{eqnarray*}
\textit{loss} = \frac{1}{n}\sum {\left( {{y_{\textit{real}}} - {y_{\textit{predict}}}} \right)^2} + 0.00001 \times {\alpha _k}\sum |\left| w \right|{|_k}
\end{eqnarray*}

Here, $n$ represents the individual number, ${y_{\textit{real}}}$ represents the observed phenotype values, ${y_{\textit{predict}}}$ represents the predicted phenotype values, 0.00001 represents the binding penalty coefficient, ${\alpha _k}$ represents the regularizer rate, and $|| w |{|_k}$ represents the *k*th norm of all weights. Parameters in the DeepAnnotation were optimized by minimum $loss$ with the back-propagation algorithm [93]. A more detailed description of DeepAnnotation can be found in Supplementary File 1. Finally, we implemented the DeepAnnotation model on a GPU (NVIDIA RTX A6000) with the Python “tensorflow” package.

### Hyperparameters

DL model performance relies on the optimization of a large number of hyperparameters. A previous study suggested that the performance of deep learning models does not significantly improve with more than 100 hyperparameter sets [94]. Instead of testing 93,600 hyperparameter sets, we used 500 randomly sampled hyperparameter combinations to reduce computation time while maintaining reasonable performance. Specifically, the hyperparameters included learning rate, feature number, regularization algorithm and rate, batch normalization momentum, and dropout rate. The ranges of values used for each hyperparameter are detailed in Supplementary Table S1. Considering the acceptable running time on the selection of an optimal hyperparameter set, we did a 2-fold cross-validation experiment to select the best hyperparameter combination based on the PCC score from those 500 hyperparameter pools. The hyperparameter optimization took a total of 409 hours, 26 minutes, and 51 seconds (approximately 17 days).

### Genotype and phenotype data

For model training, we used a public dataset including whole-genome SNPs and 3 pork production traits (LMP at 100 kg, LMD at 100 kg, BF at 100 kg), as well as 4 other traits (total teat number [TTN], left teat number [LTN], right teat number [RTN], time spent to eat per day [TPD, min]) of 2,802 Duroc boars [81]. After imputing the missing SNPs with Beagle (version 5.1) software [95], over 11.6 million (11,633,164) SNPs were encoded with a [0, 1, 2] format, corresponding to [AA, Aa, aa], where A is the major allele. After filtering out samples with missing phenotype values, 1,940 samples remained. Among these 1,940 samples, 240 were randomly selected as an independent test set and never used during the training process. Therefore, the remaining 1,700 samples were used to search for the best hyperparameter combination on LMP, LMD, and BF through a 2-fold cross-validation experiment, respectively. Subsequently, the prediction performance of 1,700 training samples was thoroughly evaluated on the LMP trait through a 5-fold cross-validation experiment, and the prediction performance of 240 independent samples was evaluated from the model trained on those 1,700 samples. In addition, LMD and BF traits were also evaluated to support the results of prediction performance comparison. Finally, the PCCs of 240 independent samples were calculated on all 7 traits by rrBLUP to evaluate the performance of different functional aspects of SNPs.

### Comprehensive multiomics functional annotation data processing

#### Epigenome data

The published epigenomic data of processed ATAC-seq were download under Gene Expression Omnibus (GEO) accession number GSE143288, including 5 pig tissues (muscle, liver, fat, spleen, and heart) from 4 breeds, including Duroc, Enshi Black (ES), Large White (LW), and Meishan (MS) (Supplementary Table S4) [96]. We also downloaded the processed ATAC-seq (GSE158414), which includes 8 Yorkshire tissues (adipose, cerebellum, cortex, hypothalamus, liver, lung, muscle, spleen) (Supplementary Table S4) [97]. Furthermore, we merged the tissues from Large White and Yorkshire, named Large White Yorkshire (LWY). We used the “intersect” command of BEDTools (version v2.25.0; RRID:SCR_006646) to define consistent peak regions from biological replicates and the “merge” command to merge the consistent peak regions from the same tissue [98].

#### Transcriptome data

The sample information of transcriptomic data was download from the “Expression Section” of the ISwine website by using the “Duroc” keyword [96]. Then, we downloaded the raw sequence data of 177 samples with a label of “Duroc” or “duroc” in Cultivar items that had been deposited in the NCBI, with the Sequence Read Archive (SRA) under the corresponding accession numbers (Supplementary Table S4). The raw sequencing reads were processed with trim_galore (version v0.6.7; RRID:SCR_011847) to remove adapters and trim low-quality bases, followed by mapping to the reference genome Sscrofa11.1 assembly using HISAT2 (version v2.2.1; RRID:SCR_015530) [99] and gene expression quantification with featureCounts (version v2.0.1; RRID:SCR_012919) [100]. Finally, 31,908 genes from 177 samples with high-quality normalized transcripts per million expression levels were prepared for further analysis.

#### Transcripts annotation data

The gene annotation information in the GTF format of the pig reference genome Sscrofa11.1 was downloaded from NCBI. We used the “dplyr” package in R to extract the gene position, the “intersect” command of BEDTools to locate SNPs in gene regions, and “window -w 1000000” to annotate potential regulatory elements within 1 Mb of genes.

#### Gene functional annotation data

The GO annotation of the pig reference genome Sscrofa11.1 was downloaded from the “BioMart” section of the Ensembl web server. In total, there were 150,226 records covering 16,784 genes and 14,682 functional terms (referred to as terms). The pathway annotation of the pig reference genome Sscrofa11.1 was downloaded from the KEGG web server. In total, there were 35,926 records covering 6,532 genes and 335 pathways (referred to as terms). We merged all 186,152 records, covering 20,037 genes and 15,017 terms.

#### Conserved regulatory elements

To incorporate the conserved regulatory elements that are critical toward understanding the genetics of complex traits, we downloaded the functionally conserved annotation (including gene body, intergenic, and promoter coordinates) of pig from Kern et al. [97]. We used the “intersect” command of BEDTools (version v2.25.0) to locate those noncoding SNPs and genes within the conserved regulatory elements regions [98].

#### Comprehensive multiomics functional annotations construction

Previous studies showed that SNPs located in coding regions could influence complex traits by altering RNA secondary structure or affecting nearby gene expression, while SNPs in noncoding regions typically affect gene expression levels [101–104]. However, only a small subset (2 of 11,941) of protein-coding genes have convincing GO annotations with evidence code IDA and TAS [105]. Therefore, to model the biological impact of both coding and noncoding SNPs in genomic selection, we employed RNAfold and DeepSEA to derive sequence-based scores for RNA minimum free energy (MFE) and chromatin accessibility, respectively. Additionally, to predict gene functions not annotated in existing GO and KEGG databases, we used easyMF to complement the functional annotations of all genes.

#### Minimum free energy calculation

In order to comprehensively represent the RNA secondary structure surrounding each SNP, we used the “BSgenome” package in R to extract 1,100 bp of DNA sequence from the Sscrofa11.1 reference genome [64, 106]. This sequence was centered on the reference and alternative alleles of the SNP, extending ±550 bp around each SNP. The “RNAfold” program (ViennaRNA version 1.8.5) was then used with the parameters “-d2 -noLP” to calculate the MFE for each sequence. Finally, the MFE values were then normalized to a range of [0, 1].

#### Cis-regulatory SNPs annotation

To predict the *cis*-regulatory effects of SNPs, we used the DeepSEA model, which learns the relationship between the genomic sequence and chromatin accessibility [64]. The model’s accuracy in recognizing open chromatin regions was evaluated using the AUC (Supplementary Fig. S3). Following the instruction of DeepSEA, during model training, the genome was split into 200-bp bins, and a bin was labeled positive if it overlapped with an ATAC-seq peak by more than 100 bp. These positive bins were then extended by 500 bp both upstream and downstream. The extended sequences were one-hot encoded into a 1,000 × 4 binary matrix, with columns corresponding to A, G, C, and T. Finally, we trained the DeepSEA model, which takes the binary matrices as input and the chromatin accessibility as output. To annotate the *cis*-regulatory effects of SNPs, we also extracted the 1,000-bp sequences containing the reference and alternative alleles of each SNP and predicted the chromatin accessibility difference of each allele pair by the trained DeepSEA model.

#### Gene functions annotation

To predict the biological functions annotation of a gene, we employed the easyMF model, which learns the associations between gene expression levels and functional terms [63]. The intuition behind easyMF is that genes sharing similar expression patterns across different cellular contexts are more likely to function in the same biological processes. The AUC was used to evaluate the accuracy of easyMF in prioritizing gene functions. Specifically, we used easyMF to decompose the gene expression matrix (containing 31,908 genes and 177 samples) into 2 low-dimensional matrices: an amplitude matrix (AM; genes in rows and metagenes in columns) and a pattern matrix (PM; metagenes in rows and samples in columns). Then, easyMF calculated the *z*-scores for each functional term by assessing the distribution of AM coefficients between genes with and without the functional term categorization [60]. The PCC was computed between the gene weights in the AM and the *z*-scores of each functional term, which were used to predict gene functions. The final completion of gene function annotation was a gene-by-functional term matrix. To further refine the annotations, a second round of matrix decomposition was performed, generating “metaterms,” which are weighted combinations of functional terms. For each “metaterm,” the functional terms with dominant patterns represent its signatures (refer to easyMF [63] for details).

#### Disjoint SNP classes based on biological prior functional annotations

For BayesRC and MBLUP models, disjoint SNP classes need to be defined based on biological prior functional annotations. For a fair comparison, the biological prior functional annotations used here were consistent with those employed in DeepAnnotation. Markers (SNPs and genes) were categorized into 4 broad categories based on the principle from MacLeod et al. [29], including the following: (a) 23,492 variants predicted to cause a nonsynonymous coding change, (b) 632,556 variants except those in (a), (c) 4,111 genes (annotated in terms with AUC >0.9) located in conserved regulatory element regions, and (d) 823 signature genes for 31 used metaterms. Finally, 5 distinct SNP classes were defined based on those 4 broad categories, including (I) 4,185 SNPs from category (a) that were located within category (c), (II) 590 SNPs from category (a) that were located within category (d), (III) 280,399 SNPs from category (b) that were located within ±50 kb of category (c), (IV) 14,923 SNPs from category (b) that were located within ±50 kb of category (d), and (V) 35,5951 other SNPs that were not in (I), (II), (III) and (IV).

#### Relative efficiency calculation

The relative efficiency (RE) is calculated based on the expected genetic gain when individuals are selected by genomic prediction [107]:


\begin{eqnarray*}
RE\left( k \right) = \frac{{\left( {\mathop \sum \nolimits_{\alpha ^{\prime}} {y_i}} \right)/{N_{\alpha ^{\prime}}} - (\mathop \sum \nolimits_{\textit{Test}} {y_i})/{N_{\textit{Test}}}}}{{\left( {\mathop \sum \nolimits_\alpha {y_i}} \right)/{N_\alpha } - (\mathop \sum \nolimits_{\textit{Test}} {y_i})/{N_{\textit{Test}}}}}
\end{eqnarray*}

Here, $\alpha $ and $\alpha ^{\prime}$ are the groups of extreme individuals selected by the ranking of real observed or predicted phenotypic values, respectively; $k = {\mathrm{\ }}{N_{\alpha ^{\prime}}} = {\mathrm{\ }}{N_\alpha }$ are the numbers of individuals in each group; ${y_i}$ is the real observed phenotypic value of the *i*th individual; and $(\mathop \sum \limits_{\textit{Test}} {y_i})/{N_{\textit{Test}}}$ represents the mean of the test group.

#### Heritability calculation

To calculate the heritability, we first extracted the weights of all SNPs trained by each model through 5-fold cross-validation. The significance levels of these SNPs were determined using a meta-strategy with multiple testing correction implemented in the “RobustRankAggreg” R package. SNPs with an adjusted *P* value <0.05 were considered potential causal variants. For the real LMP trait, the heritability was estimated as the proportion of genetic variance explained by the predicted causal variants, calculated using the BLUP model implemented in GCTA software (“–reml-pred-rand –reml-est-fix –blup-snp”) [89].

#### Dual-luciferase reporter assay

The dual-luciferase reporter assay was conducted to assess the functional impact of the SNPs. The target fragment was inserted into the PGL4.23 vector in front of the promoter. During C2C12 cell culture and transfection, the culture medium consisted of 90% Dulbecco’s modified Eagle’s medium, 10% serum, and 1% penicillin/streptomycin. The cells were seeded in 24-well plates and transfected using jetPRIME transfection reagent. The transfection procedure was performed with 1.0 µg plasmid per well (target plasmid to internal reference plasmid ratio: 5:1) and 2 µL transfection reagent with 200 µL jetPRIME. The target fragment was inserted into the PGL4.23 carrier, which expresses firefly luciferase, while the internal reference plasmid expressed sea cucumber luciferase. The 2 plasmids were introduced into the cells at a 5:1 ratio. After 24 hours of incubation, the cells were harvested for further analysis. The cells were lysed, and the fluorescence values for firefly luciferase and sea cucumber luciferase were measured using their respective reaction substrates. The relative luciferase activity was calculated as the ratio of the firefly fluorescence to the sea cucumber fluorescence, providing an internal control for normalization. The 101-bp target sequences for the reference (wild-type, WT) and alternative (MUT) alleles of the SNPs were as follows: chr10:23833431 (WT: G): GCAAAGGGGCTGCCCTGGCATGACCCTCGTCTTTGGGGACACTGGGACAAGGGCAAGGACATTGGAAAAAGCCTGGCCTCTGCCCAGGAACTAACTGGGGC, and alternative allele A (MUT: A): GCAAAGGGGCTGCCCTGGCATGACCCTCGTCTTTGGGGACACTGGGACAAAGGCAAGGACATTGGAAAAAGCCTGGCCTCTGCCCAGGAACTAACTGGGGC. chr4:95182500 (WT: G): CGCAGCTGCAGAATTTCAGAGCTGAAAGAGACATTCAGAATTATTGAGTCGAATCTCCTCTCTTGGCAGATAAGAAATGAGACCAAGAACATGTACCTAAC, and alternative allele A (MUT: A): CGCAGCTGCAGAATTTCAGAGCTGAAAGAGACATTCAGAATTATTGAGTCAAATCTCCTCTCTTGGCAGATAAGAAATGAGACCAAGAACATGTACCTAAC. This method allowed us to assess the relative transcriptional activity of the WT and MUT alleles by comparing the luciferase signals.

## Availability of Source Code and Requirements

Source: GitHub repository [108]

Project name: DeepAnnotation

Project homepage: https://github.com/mawenlong2016/DeepAnnotation

Operating system: Linux

Programming language: Python 3.6

Other requirements: conda, matplotlib, scikit-learn, numpy, tensorflow-gpu, framework-reproducibility

License: GPL -3.0

biotoolsID: deepannotation

RRID:SCR_026630

Source: Zenodo repository [109]

Project name: DeepAnnotation: A novel interpretable deep learning–based genomic selection model that integrates comprehensive functional annotations

Link: https://doi.org/10.5281/zenodo.8410693

License: CC0

Source: DOME annotations [110]

Project name: DeepAnnotation: A novel interpretable deep learning–based genomic selection model that integrates comprehensive functional annotations

Link: https://dome.dsw.elixir-europe.org/wizard/projects/4acf509b-3d08-4f6f-b4a9-46239ca43c6d

## Abbreviations

AUC: area under the receiver operating characteristic curve; BayesR: hierarchical Bayesian mixture; BayesRC: Bayesian genomic prediction with disjoint annotations; BF: back fat thickness at 100 kg; BLUP: best linear unbiased prediction; BMP: bone morphogenetic protein; bp: base pair; CBP: complex biological process; CV: cross-validation; DL: deep learning; DNN: deep neural network; GEBV: genomic estimated breeding value; GP: genomic prediction; GS: genomic selection; GWAS: genome-wide association studies; KAML: kinship-adjusted multiple-loci linear mixed model; LightGBM: light gradient boosting machine; LMD: loin muscle depth at 100 kg; LMP: lean meat percentage at 100 kg; LWY: large white Yorkshire; MBLUP: best linear unbiased prediction with multiple random effects; MFE: minimum free energy; PCC: Pearson correlation coefficient; rrBLUP: ridge regression best linear unbiased prediction; WGS: whole-genome sequencing.

## Additional Files

**Supplementary File 1**. The algorithm, implementation, and interpretation of DeepAnnotation.

**Supplementary Fig. S1**. Fine mapping through the interpretable deep learning model.

**Supplementary Fig. S2**. Average PCC from different sets of SNPs with the rrBLUP model.

**Supplementary Fig. S3**. ROC curves of ATAC data learned from the DeepSEA model for different tissues of different species.

**Supplementary Table S1**. PCC scores of different hyperparameter combinations based on 2-fold cross-validation.

**Supplementary Table S2**. Statistical information of signatures (terms) for significant metaterms.

**Supplementary Table S3**. Statistical information of genes and noncoding SNPs with significant contributions of LMP via metaterm8.

**Supplementary Table S4**. Summary information of epigenomic and transcriptomic data.

giaf083_Supplemental_Files

giaf083_Authors_Response_To_Reviewer_Comments_original_submission

giaf083_Authors_Response_To_Reviewer_Comments_Revision_1

giaf083_Authors_Response_To_Reviewer_Comments_Revision_2

giaf083_GIGA-D-25-00002_original_submission

giaf083_GIGA-D-25-00002_Revision_1

giaf083_GIGA-D-25-00002_Revision_2

giaf083_GIGA-D-25-00002_Revision_3

giaf083_Reviewer_1_Report_Original_Submission Felix Heinrich -- 2/24/2025

giaf083_Reviewer_1_Report_Revision_1 Felix Heinrich -- 3/25/2025

giaf083_Reviewer_2_Report_Original_Submission Ruidong Xiang -- 2/27/2025

giaf083_Reviewer_2_Report_Revision_1 Ruidong Xiang -- 4/21/2025

giaf083_Reviewer_2_Report_Revision_2 Ruidong Xiang -- 6/10/2025

## Data Availability

The large-scale whole-genome sequencing dataset of the Duroc pig population can be directly downloaded from the *GigaScience* database, GigaDB [111]. The published epigenomic data of processed ATAC-seq can be directly downloaded under Gene Expression Omnibus (GEO), accession number GSE143288 [112]. The published epigenomic data of processed ATAC-seq of Yorkshire can be directly downloaded under GEO accession number GSE158414 [113]. The sample information of transcriptomic data can be directly obtained from the “Expression Section” of the ISwine website [114] by using the “Duroc” keyword and then directly downloaded under the corresponding accession numbers from the NCBI Sequence Read Archive [115]. The gene annotation information in GTF format of the pig reference genome Sscrofa11.1 can be directly downloaded from NCBI [116]. The GO annotation of the pig reference genome Sscrofa11.1 can be directly downloaded from the “BioMart” section of the Ensembl web server [117]. The pathway annotation of the pig reference genome Sscrofa11.1 can be directly downloaded from the KEGG web server [118]. The transformed genotype data containing 11,633,164 SNPs from 1,940 samples; the phenotype data containing 3 pork production traits from 1,940 samples; the comprehensive functional annotation data for Duroc prepared by RNAfold, DeepSEA, and easyMF models; and the 4 types of input data, as well as several additional data files for the training DeepAnnotation model, are openly available in the DOI-assigning repository Zenodo [109, 119]. Machine learning annotations for DeepAnnotation have been deposited in the DOME registry [110]. The source code for building the comprehensive functional annotations using RNAfold, DeepSEA, and easyMF is freely available through GitHub [120]. The source code of DeepAnnotation is freely available through GitHub [79] and Docker [80].
