# Supplementary material for: DeepAnnotation: A novel interpretable deep learning–based genomic selection model that integrates comprehensive functional annotations
Source: Gigascience. 2025 Aug 28;14:giaf083. doi: 10.1093/gigascience/giaf083 (PMC12392413; doi:10.1093/gigascience/giaf083)
Supplement: giaf083_Supplemental_Files [file giaf083_supplemental_files.zip › Supplementary File 1.docx]

**DeepAnnotation: A novel interpretable deep learning-based genomic selection model that integrates comprehensive functional annotations**

Wenlong Ma^1,2,†^, Weigang Zheng^1,2,4,†^, Shenghua Qin^1,2^, Chao Wang^1,2^, Bowen Lei^1,2^, and Yuwen Liu^1,2,3,*^

^1^Shenzhen Branch, Guangdong Laboratory for Lingnan Modern Agriculture, Key Laboratory of Livestock and Poultry Multi-Omics of MARA, Agricultural Genomics Institute at Shenzhen, Chinese Academy of Agricultural Sciences, Shenzhen 518124, China.

^2^Innovation Group of Pig Genome Design and Breeding, Research Centre for Animal Genome, Agricultural Genomics Institute at Shenzhen, Chinese Academy of Agricultural Sciences, Shenzhen 518124, China.

^3^Kunpeng Institute of Modern Agriculture at Foshan, Chinese Academy of Agricultural Sciences, Foshan 528226, China.

^4^Key Laboratory of Agricultural Animal Genetics, Breeding and Reproduction of Ministry of Education & Key Lab of Swine Genetics and Breeding of Ministry of Agriculture and Rural Affairs, Huazhong Agricultural University, Wuhan 430070, People’s Republic of China.

†Equal contribution.

*Correspondence address: Shenzhen Branch, Guangdong Laboratory for Lingnan Modern Agriculture, Key Laboratory of Livestock and Poultry Multi-Omics of MARA, Agricultural Genomics Institute at Shenzhen, Chinese Academy of Agricultural Sciences, Shenzhen, China; E-mail: liuyuwen@caas.cn

**DeepAnnotation algorithm**

In DeepAnnotation framework, layers are available built from biological knowledge, such as genotype, epigenomic features, protein secondary structure data, transcriptomic profiles, gene function annotations, regulatory module metaterms. Information from these resources is used to define adjusted transcriptional regulatory functional annotations, shaping an interpretable neural network, allowing the evaluation of millions of genetic markers belonging to a specific co-regulatory module together. The dual dense neural network is applied in each visible layer to directly quantify the attribute of each genetic marker to final phenotype, besides convolutional neural network is also supported.

The use of prior knowledge restricts the network layout, for example, the number of neurons and the number of layers is determined by the prior knowledge embedded in the network. In DeepAnnotation, we used 7 layers represent different types of these prior knowledge driven from different omics data, the biological systems multi-omics from the genome to final phenome has been detailly described by Ritchie et al. (2015) [1]:

Layer 1: visible to receive the genome annotations: Basic genotype data with a coding scheme of [0,1,2] or [-1,0,1] to represent Ref/Ref, Ref/Alt or Alt/Ref, Alt/Alt, besides other formats (such as [-1,1] representing the presence or absence status of an allele) are also supported.

We divided the SNPs into non-coding and coding schemes represent regulatory variants and functional variants. The outputs of input variants were defined as:

$y=f(wx+b)$ (1)

Where $x$ represents the genotype value of each variant, $w$ represents the weight of the variant, $b$ represents the bias. To reduce the overfitting of wide-range of weights on model, we used kernel regularizer to constraint them, the penalty was defined as:

$$\left\{ \begin{aligned} L1 norm: \alpha_{1}\sum_{i=0}^{n} |{|w||}_{1} \\ L2 norm: \alpha_{2}\sum_{i=0}^{n} |{|w||}_{2} \\ L1_{L2}norm: \alpha_{1}\sum_{i=0}^{n} |{|w||}_{1}+\alpha_{2}\sum_{i=0}^{n} |{|w||}_{2} \end{aligned} \right.$$

The stronger the weight is the more it contributes to the final prediction of the network [2]. In DeepAnnotation, we used these weights represent the relative contribution of genetic markers to final phenotype. Totally, in Layer 1, $y_{non-coding}$ and $y_{coding}$ represent the output of regulatory and functional variants, respectively.

Layer 2: visible to receive the epigenomic and secondary structure annotations: chromatin accessibility was quantified by DeepSEA model for non-coding SNPs, minimum free energy was quantified by RNAfold model for coding SNPs. We combined the outputs of regulatory variants from genome and epigenome annotations, the outputs of functional variants from genome and secondary structure annotations, respectively, by the ‘concatenate’ function defined in tensorflow package (version 2.2.0):

$$y= tf.keras.layers.concatenate([y_{genome},y_{epigenome or secondary structure}])$$

Where $y_{regulatory}$ represents the output of adjusted regulatory variants, $y_{functional}$ represents the output of adjusted functional variants. For a fair interpretation of the weights, we proceeded batch normalization on $y_{regulatory}$ and $y_{functional}$, following by ‘ReLU’ activation and dropout operation. The batch normalization was done by ‘batch_normalization’ function with epsilon set to 1e-05, the ‘ReLU’ was defined by the following formula:

$$\text{ReLU}\left( x \right)=\left\{ \begin{aligned} x, where x>0 \\ 0, where x\leq0 \end{aligned} \right.$$

Layer 3: visible to receive the transcriptomic and gene function annotations: expression patterns were extracted by easyMF and further quantified the functional properties of genes annotated in GO (http://geneontology.org/) and KEGG (http://www.genome.jp/kegg/). The outputs of gene functions annotations ($y_{gene}$) were also defined by Equation (1). It should be noted that, in this layer, the main features were focus on gene functions, thus, we combined the outputs of them with those defined in regulatory variants and functional variants by the following formula:

$$y= tf.keras.layers.concatenate([y_{gene},y_{regulatoy},y_{functional}])$$

Then, the $y_{function}$ was proceed with batch normalization, ReLU activation, and dropout.

Layer 4: visible to receive the regulatory modules annotations: co-regulatory metaterms were extracted by matrix factorization algorithm. The outputs of co-regulatory metaterms annotations ($y_{metaterm}$) were also defined by Equation (1). Then, we combined the outputs of $y_{metaterm}$with $y_{functions}$ by the following formula:

$$y= tf.keras.layers.concatenate([y_{metaterm},y_{function})$$

Then, the $y_{high-order}$ was proceed with batch normalization, ReLU activation, and dropout.

Layer 5 and Layer 6: these 2 layers were invisible, and were designed to summarize those aforementioned transcriptional regulatory functional annotations based on the inherent feature aggregation of deep neural network (DNN). We directly used the Equation (1) to pass the outputs following by batch normalization, ReLU activation, and dropout.

Layer 7: visible to receive the final output defined by all above layers corresponding to phenotype. We used linear regression algorithm by the Equation (1) with only kernel regularizer to adjust the weights.

**DeepAnnotation implementation**

The hyperparameters combinations were optimized based on a 2-fold cross-validation. In this experiment, 1700 training samples were randomly portioned into 2 groups with approximately equal size. In each circle, 425 samples were used to train the model, 425 samples were used to valid the performance of the model, the model training was finished when the validation loss was not decreased within the following 600 epochs. The loss function was defined by the following formula:

$$loss=\frac{1}{n}\sum{(y_{real}-y_{predict})}^{2}+0.00001\times\alpha_{k}\sum|{|w||}_{k}$$

Then, the phenotypes of the rest 850 samples were predicted using the optimized model. Finally, the Pearson correlation coefficient of on hyperparameter combination was calculated based on the predicted and real phenotypes of all 1700 samples.

**DeepAnnotation interpretation**

We retrained the DeepAnnotation using all prior biological functional annotations to ensure to obtain the weights of all genetic markers. We used ‘saver.restore’ function defined in tensorflow package to exemplify retrieve the trained weights at 382 training epochs by all 1700 training samples of LMP trait. Totally, 65706 coding SNPs (we conserved only those located in gene region), 590342 non-coding SNPs (we conserved those located in Duroc ATAC peak region or located in conserved regulatory elements regions), 4111 genes (annotated in terms with AUC>0.9 that located in conserved regulatory elements regions), 14996 terms and 31 metaterms were trained in our DeepAnnotation.


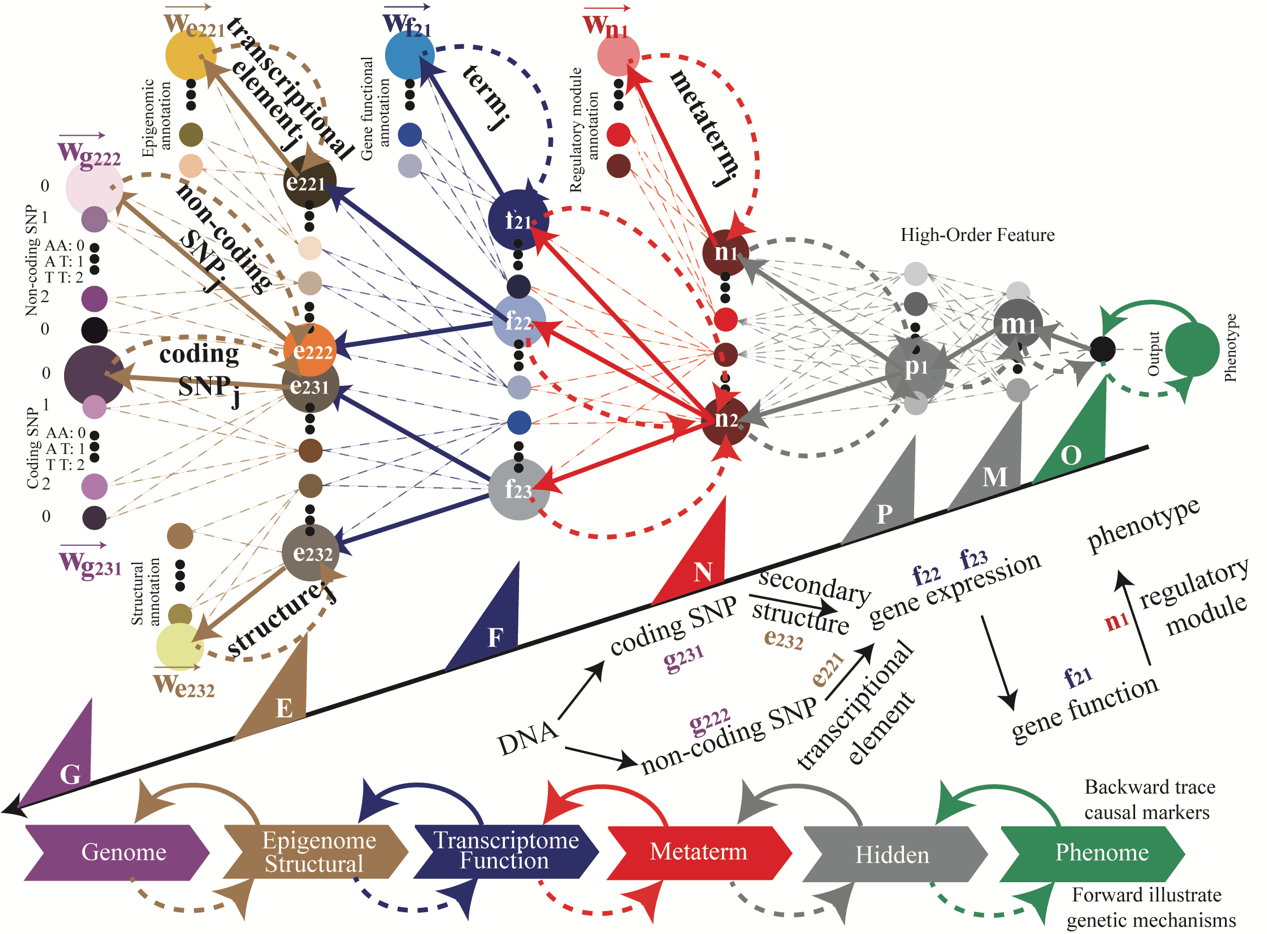


**Fig. 1 Fine mapping through interpretable deep learning model.**

Starting from the top node O, we extracted the weights $w_{M\to O}$ from the sixth layer M (Fig. 1). We found the maximum node in $w_{M\to O}$ by the following script in R:

$which.max(abs(w))$ (2)

The most significant node $m_{1}= which.max(abs(w_{M\to O}))$ of M contribute to O could be obtained by Equation (2), and we found the most significant node $p_{1}= which.max(abs(w_{P\to m_{1}}))$ of P contribute to m1. It should be noted that the inputs of p1 defined by $y_{high-order}$driven from two parts: $y_{metaterm}$ and $y_{function}$, thus, we identified node $n_{1}= which.max(abs(w_{N\to p_{1}}))$ and $n_{2}= which.max(abs(w_{N\to p_{1}}))$ for $y_{metaterm}$ and $y_{function}$ of fourth layer N. The weights of $\vec{n_{1}}$ contained the contribution of co-regulatory modules annotation with 14996 terms from 31 metaterms, and further the relative contribution of each metaterm was calculated by the rank of mean values of 14996 terms by the following formula:

$${rank}_{metaterm}=rank(\frac{1}{k}\sum_{i=1}^{k} abs(w_{{n1}_{i}}),ties.method="min")$$

Where, $k$ represents the number of metaterms, $\vec{w_{n1}}$ represents the weight of $n_{1}$ node, $rank(\cdot)$ was utilized by ‘rank’ function in R.

Based on the same strategy, we could obtain all the ranks of causal genetic markers with significant contributions to final phenotype via the same co-regulatory module, defined by the following formula:

$$\left\{ \begin{aligned} m_{1}= which.max(abs\left( w_{M\to O} \right)) \\ p_{1}= which.max(abs(w_{P\to m_{1}})) \\ n_{1}= which.max(abs(w_{N_{metaterm}\to p_{1}})) \\ n_{2}= which.max(abs(w_{N_{function}\to p_{1}})) \\ {rank}_{metaterm}=rank(\frac{1}{14996}\sum_{i=1}^{14996} abs(w_{{n1}_{i}}),ties.method="min") \\ {rank}_{term}=rank(\frac{1}{31}\sum_{i=1}^{31} abs(w_{{n1}_{i}}),ties.method="min") \\ f_{21}= which.max(abs(w_{F_{gene}\to n_{2}})) \\ {rank}_{gene}=rank(\frac{1}{4111}\sum_{i=1}^{4111} abs(w_{{f21}_{i}}),ties.method="min") \\ f_{22}= which.max(abs(w_{F_{regulatoy}\to n_{2}})) \\ f_{23}= which.max(abs(w_{F_{functional}\to n_{2}})) \\ e_{221}= which.max(abs(w_{E_{epigenome}\to f_{22}})) \\ {rank}_{regulatory element}=rank(abs(w_{e221}),ties.method="min") \\ e_{222}= which.max(abs(w_{G_{non-coding}\to f_{22}})) \\ {rank}_{non-coding SNP}=rank(abs(w_{g222}),ties.method="min") \\ e_{231}= which.max(abs(w_{G_{coding}\to f_{23}})) \\ {rank}_{cding SNP}=rank(abs(w_{g231}),ties.method="min") \\ e_{232}= which.max(abs(w_{G_{secondary structure}\to f_{23}})) \\ {rank}_{secondary structure}=rank(abs(w_{g232}),ties.method="min") \end{aligned} \right.$$

We utilized 5 CV experiment strategy to train the model, and used ensemble algorithm to calculate the significant levels by ‘aggregateRanks’ function with multiple testing correction in R [3].

Finally, we could back forward trace the causal markers from O->M->P->N->F->E->G, and forward illustrate genetic mechanism from G->E->F->N->P->M->O with two genetic information flows:

i: non-coding SNP $g222$ acted as regulatory element, it regulates the expression of gene $e_{222}$, functionalizing as term $f_{21}$, participating in regulatory module $n_{1}$, contributing to final phenotype.

ii: mutation occurs at coding SNP $g232$ changed the secondary structure of gene $e_{232}$, altering its expression and its function $f_{21}$, participating in regulatory module $n_{1}$, contributing to final phenotype.

**Reference**

1. Ritchie MD, Holzinger ER, Li R, et al. Methods of integrating data to uncover genotype-phenotype interactions. Nat Rev Genet. 2015;16:85-97.

2. van Hilten A, Kushner SA, Kayser M, et al. GenNet framework: interpretable deep learning for predicting phenotypes from genetic data. Commun Biol. 2021;4:1094.

3. Kolde R, Laur S, Adler P, et al. Robust rank aggregation for gene list integration and meta-analysis. Bioinformatics. 2012;28:573-580.
