# Supplementary figures and images for: DeepAnnotation: A novel interpretable deep learning–based genomic selection model that integrates comprehensive functional annotations
Source: Gigascience. 2025 Aug 28;14:giaf083. doi: 10.1093/gigascience/giaf083 (PMC12392413; doi:10.1093/gigascience/giaf083)

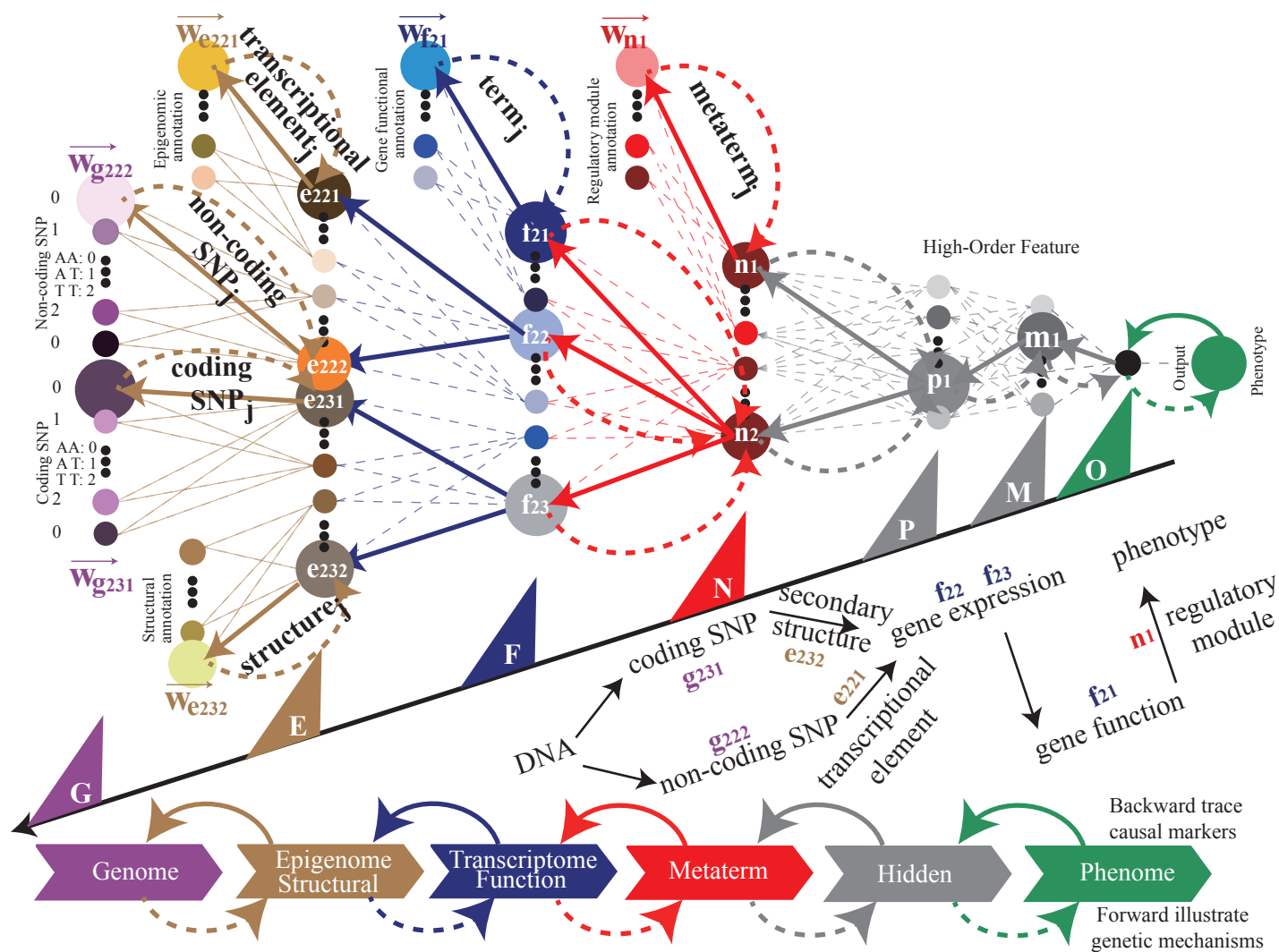

Supplement: giaf083_Supplemental_Files [file giaf083_supplemental_files.zip › Supplementary Figure S1.pdf]

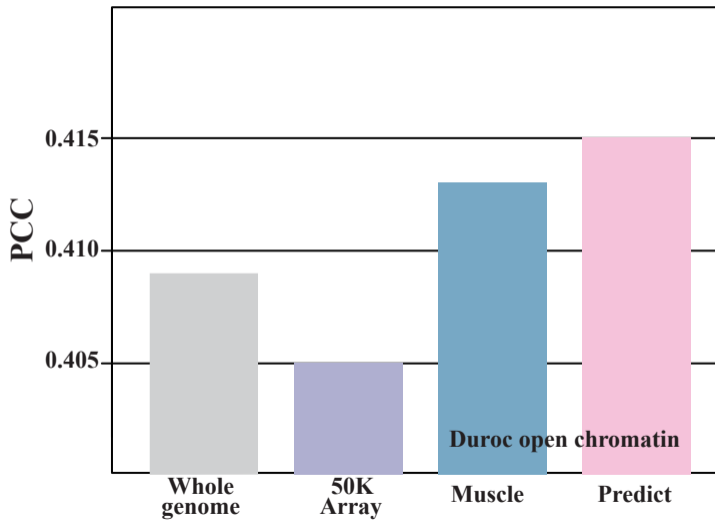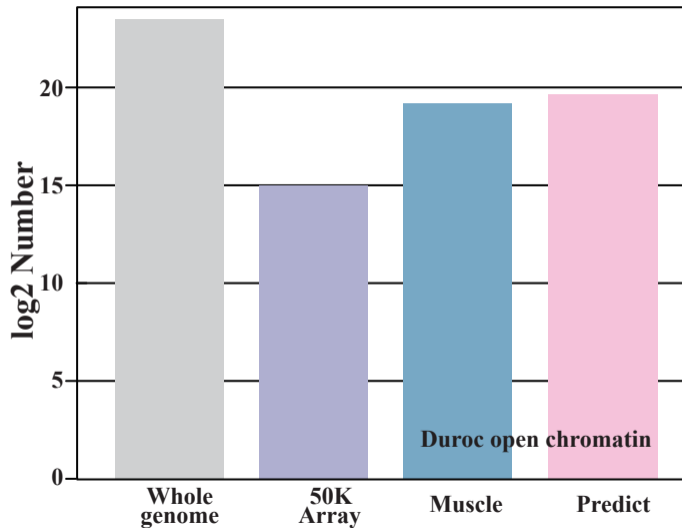

Supplement: giaf083_Supplemental_Files [file giaf083_supplemental_files.zip › Supplementary Figure S2.pdf]

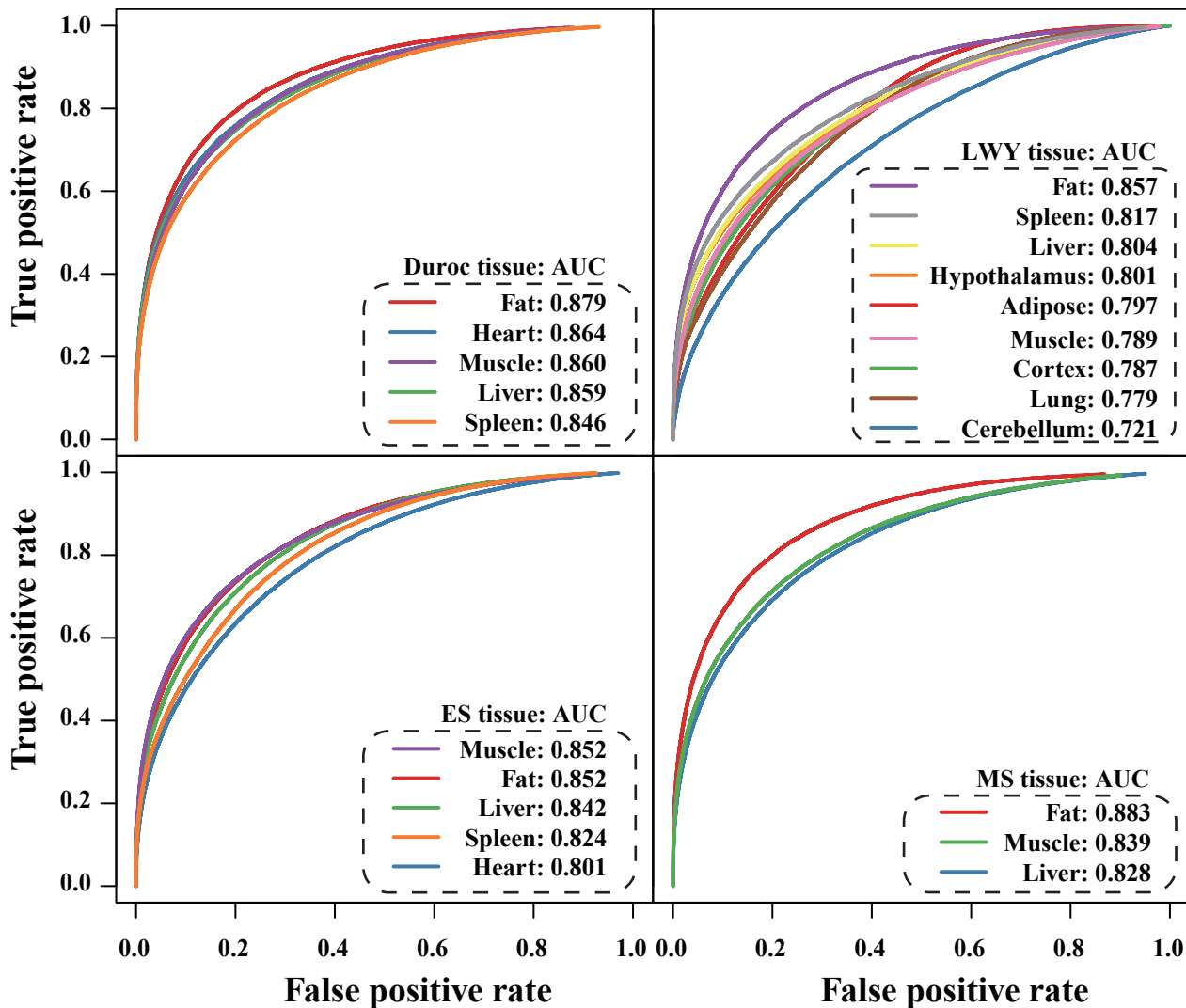

Supplement: giaf083_Supplemental_Files [file giaf083_supplemental_files.zip › Supplementary Figure S3.pdf]
