# Supplementary material for: DeepAnnotation: A novel interpretable deep learning–based genomic selection model that integrates comprehensive functional annotations
Source: Gigascience. 2025 Aug 28;14:giaf083. doi: 10.1093/gigascience/giaf083 (PMC12392413; doi:10.1093/gigascience/giaf083)
Supplement: giaf083_GIGA-D-25-00002_Revision_3 [file giaf083_giga-d-25-00002_revision_3.pdf]

GigaScience

DeepAnnotation: A novel interpretable deep learning-based genomic selection model that integrates comprehensive functional annotations

--Manuscript Draft--

Manuscript Number:	GIGA-D-25-00002R3
Full Title:	DeepAnnotation: A novel interpretable deep learning-based genomic selection model that integrates comprehensive functional annotations
Article Type:	Technical Note
Funding Information:	National Key R&D Program of China (2021YFF1000600) — Dr. Yuwen Liu
Abstract:	Background: Genomic selection, which leverages genomic information to predict the breeding value of individuals, has dramatically accelerated the improvement of economically important traits. The growing availability of multi-omics data in agricultural species offers an unprecedented opportunity to enrich this process with prior biological knowledge. However, fully harnessing these rich data sources for accurate phenotype prediction in genomic selection remains in its early stages. Results: In this study, we present DeepAnnotation, a novel interpretable genomic selection model designed for phenotype prediction by integrating comprehensive multi-omics functional annotations using deep learning. To capture the complex information flow from genotype to phenotype, DeepAnnotation aligns multi-omics biological annotations with sequential network layers in a deep learning architecture, mirroring the natural regulatory cascade from genotype to intermediate molecular phenotypes—such as cis-regulatory elements, genes, and gene modules—and ultimately to phenotypes of economic traits. Comparing against seven classical models (rrBLUP, LightGBM, KAML, BLUP, BayesR, MBLUP, and BayesRC), DeepAnnotation demonstrated significantly superior prediction accuracy (PCC increased by 6.4% to 120.0%) and computational efficiency for three pork production traits (LMP, LMD, and BF) using a dataset of 1,700 training Duroc boars and 240 independent validation individuals, each genotyped for 11,633,164 SNPs, particularly in identifying top-performing individuals. Furthermore, the interpretability embedded within our framework enables the identification of potential causal SNPs and the exploration of their mediated molecular mechanisms underlying trait variation. Conclusions: DeepAnnotation is an open-source, interpretable deep learning approach for phenotype prediction, leveraging comprehensive multi-omics functional annotations. Freely accessible via GitHub and Docker, it provides a valuable tool for researchers and practitioners in genomic selection.
Corresponding Author:	Yuwen Liu<br>Chinese Academy of Agricultural Sciences Agricultural Genomes Institute at Shenzhen<br>Shenzhen, CHINA
Corresponding Author Secondary Information:	
Corresponding Author's Institution:	Chinese Academy of Agricultural Sciences Agricultural Genomes Institute at Shenzhen
Corresponding Author's Secondary Institution:	
First Author:	Wenlong Ma
First Author Secondary Information:	
Order of Authors:	Wenlong Ma
	Weigang Zheng
	Shenghua Qin
	Chao Wang
	Bowen Lei
	Yuwen Liu

Powered by Editorial Manager® and ProduXion Manager® from Aries Systems Corporation

**Order of Authors Secondary Information:**	
**Response to Reviewers:**	We would like to express our sincere gratitude to the editor and reviewers for their constructive and insightful comments. Their feedback has been invaluable in enhancing the quality of our manuscript. All authors have thoroughly discussed each comment and carefully revised the manuscript to meet the high standards of your esteemed journal. Below, we provide a detailed, point-by-point response to the reviewers' comments, along with a summary of the corresponding modifications made in the revised manuscript.<br>Following the editor's comments, we have registered DeepAnnotation in bio.tools (biotoolsID: deepannotation) and SciCrunch.org (RRID: SCR_026630). Additionally, all previous comments from the editor have been incorporated into the revised manuscript.<br>Since our responses to the reviewers' comments include figures and tables, we have provided the point-by-point responses as supplementary materials (attached as pointby- point response to reviewers' comments.docx). We kindly request the reviewers to review our revisions by referring to this attachment.
**Additional Information:**	
**Question**	**Response**
Are you submitting this manuscript to a special series or article collection?	No
**Experimental design and statistics**<br><br>Full details of the experimental design and statistical methods used should be given in the Methods section, as detailed in our Minimum Standards Reporting Checklist. Information essential to interpreting the data presented should be made available in the figure legends.<br><br>Have you included all the information requested in your manuscript?	Yes
**Resources**<br><br>A description of all resources used, including antibodies, cell lines, animals and software tools, with enough information to allow them to be uniquely identified, should be included in the Methods section. Authors are strongly encouraged to cite Research Resource Identifiers (RRIDs) for antibodies, model organisms and tools, where possible.<br><br>Have you included the information requested as detailed in our Minimum Standards Reporting Checklist?	Yes

Powered by Editorial Manager® and ProduXion Manager® from Aries Systems Corporation

**Availability of data and materials**<br><br>All datasets and code on which the conclusions of the paper rely must be either included in your submission or deposited in publicly available repositories (where available and ethically appropriate), referencing such data using a unique identifier in the references and in the “Availability of Data and Materials” section of your manuscript.<br><br>Have you have met the above requirement as detailed in our Minimum Standards Reporting Checklist?	Yes
GigaScience has policies and guidelines in place for the use of generative AI-writing tools such as ChatGPT. If you have used such writing tools to assist with writing the manuscript this must be declared and cited in the text. Authors should not list AI-writing tools and other AI-assisted technologies as an author or co-author and should acknowledge that they are fully responsible for text generated or refined by AI-writing tools.<p><br><br>A summary of use (particularly in the introduction or among methods) needs to be included at the end of the paper, and the outputs should also be included as a supplementary file hosted in GigaDB or other open repositories. Please read our guidelines for more information. <p><br><br>By submitting to GigaScience, you are aware of the journal's AI-writing tools policy, and if you have declared use of such tools below, you have acknowledged this where appropriate in your manuscript and have made a summary of use and outputs available. <p><br><b>AI-assisted writing tools have been used in the preparation of this manuscript?	No

Manuscript
Click here to access/download;Manuscript;250623-DeepAnnotation revised manuscript submit-250623.docx

DeepAnnotation: A novel interpretable deep learning-based genomic selection model that integrates comprehensive functional annotations

Wenlong Ma[1,2,†], Weigang Zheng[1,2,4,†], Shenghua Qin[1,2], Chao Wang[1,2], Bowen Lei[1,2], and Yuwen Liu[1,2,3,*]

[1]Shenzhen Branch, Guangdong Laboratory for Lingnan Modern Agriculture, Key Laboratory of Livestock and Poultry Multi-Omics of MARA, Agricultural Genomics Institute at Shenzhen, Chinese Academy of Agricultural Sciences, Shenzhen 518124, China.

[2]Innovation Group of Pig Genome Design and Breeding, Research Centre for Animal Genome, Agricultural Genomics Institute at Shenzhen, Chinese Academy of Agricultural Sciences, Shenzhen 518124, China.

[3]Kunpeng Institute of Modern Agriculture at Foshan, Chinese Academy of Agricultural Sciences, Foshan 528226, China.

[4]Key Laboratory of Agricultural Animal Genetics, Breeding and Reproduction of Ministry of Education & Key Lab of Swine Genetics and Breeding of Ministry of Agriculture and Rural Affairs, Huazhong Agricultural University, Wuhan 430070, People’s Republic of China.

[†]Equal contribution.

[*]Correspondence address: Shenzhen Branch, Guangdong Laboratory for Lingnan Modern Agriculture, Key Laboratory of Livestock and Poultry Multi-Omics of MARA, Agricultural Genomics Institute at Shenzhen, Chinese Academy of Agricultural Sciences, Shenzhen, China; E-mail: liuyuwen@caas.cn

ORCID iDs: Wenlong Ma [0000-0002-7655-8612]; Yuwen Liu [0009-0001-0470-2355];

Abstract

Background: Genomic selection, which leverages genomic information to predict the breeding value of individuals, has dramatically accelerated the improvement of economically important traits. The growing availability of multi-omics data in agricultural species offers an unprecedented opportunity to enrich this process with prior biological knowledge. However, fully harnessing these rich data sources for accurate phenotype prediction in genomic selection remains in its early stages. Results: In this study, we present DeepAnnotation, a novel interpretable genomic selection model designed for phenotype prediction by integrating comprehensive multi-omics functional annotations using deep learning. To capture the complex information flow from genotype to phenotype, DeepAnnotation aligns multi-omics biological annotations with sequential network layers in a deep learning architecture, mirroring the natural regulatory cascade from genotype to intermediate molecular phenotypes—such as cis-regulatory elements, genes, and gene modules—and ultimately to phenotypes of economic traits. Comparing against seven classical models (rrBLUP, LightGBM, KAML, BLUP, BayesR, MBLUP, and BayesRC), DeepAnnotation demonstrated significantly superior prediction accuracy (PCC increased by 6.4% to 120.0%) and computational efficiency for three pork production traits (LMP, LMD, and BF) using a dataset of 1,700 training Duroc boars and 240 independent validation individuals, each genotyped for 11,633,164 SNPs, particularly in identifying top-performing individuals. Furthermore, the interpretability embedded within our framework enables the identification of potential causal SNPs and the exploration of their mediated molecular mechanisms underlying trait variation. Conclusions: DeepAnnotation is an open-source, interpretable deep learning approach for phenotype prediction, leveraging comprehensive multi-omics functional annotations. Freely accessible via GitHub and Docker, it provides a valuable tool for researchers and practitioners in genomic selection.

Keywords: Genomic selection, Deep learning, Multi-omics functional annotations, Intermediate molecular phenotypes, Interpretability, Causal SNPs

Background

Throughout domestication, agricultural species have undergone a deliberate and sustained process of selective breeding aimed at enhancing desirable traits. This elegantly organized practice has led to significant genetic advancements in livestock, poultry, crops, and aquaculture over successive generations [1-4]. In recent years, genomic selection (GS), which uses genomic information to predict economic traits, has substantially revolutionized breeding programs [5]. The core principle of GS, also known as genomic prediction (GP), involves predicting the genomic estimated breeding values (GEBVs) of individuals using phenotype prediction models based on genome-wide DNA markers, genotyped via microarrays or high-throughput next-generation sequencing [6-8]. This shift from traditional phenotype-based selection to genotype-based selection has dramatically accelerated genetic gains by increasing precision and expediting decision-making [9-11]. It has enabled the creation of core germplasm resources and accelerated the breeding programs across various agricultural species [8, 12].

One of the main challenges in applying GS to breeding programs is the "$p >> n$" problem, where the number of genotypic markers (p) is often much larger than the population size (n). For instance, hundreds of thousands, or even millions, of SNPs must be analyzed in relatively small populations [13]. To address this challenge, the best linear unbiased prediction (BLUP) method was proposed,

assuming that SNP effects were drawn from a normal distribution with mean zero and constant non-zero variance [14, 15]. BLUP has been widely used for complex trait prediction, significantly advancing biological breeding [5, 16]. Another prominent BLUP-based GS tool is the ridge regression best linear unbiased prediction (rrBLUP) model, which applies ridge regression and is equivalent to BLUP when genetic covariance between lines is proportional to their similarity in genotype space [17, 18]. rrBLUP performed well in phenotype prediction, particularly when the heritability is high, where additive genetic effects dominate and could be well fitted through linear algorithms [13, 19-22]. However, experimental evidence showed that a large proportion of SNPs may have no effects on a specific trait, while others may exhibit small or large effects [23, 24], suggesting that the non-zero SNP effects estimated by BLUP-based model may overlook important aspects of genetic variance [25]. To address this limitation, the Bayesian-based mixture model called BayesR was introduced, assuming SNP effects follow a mixture of normal distributions, including a point mass at zero [26]. BayesR has proven to be a powerful and flexible tool for genomic prediction, offering insights into the genetic architecture of complex traits [27, 28]. Benefiting from its high scalability, BayesR could be extended to incorporate prior biological information, as demonstrated by the state-of-the-art BayesRC model [29]. In BayesRC, the genome is divided into disjoint categories, each with potentially different proportions of SNP effects drawn from a mixture of normal distributions, significantly improving both genomic prediction accuracy and the power of genomic mapping [30-32]. Moreover, as an extension of BLUP model, MultiBLUP (hereafter called MBLUP) successfully integrates prior biological information, offering better prediction performance and computational efficiency than alternative methods by utilizing multiple genomic relation matrixes (GRMs) [33].

Both BLUP and Bayesian-based models rely on linear algorithms, which may not fully capture the complexity of traits involving non-additive effects [34, 35]. To better model these complex genetic architectures, machine learning-based approaches have been developed. One notable example is the light gradient boosting machine (LightGBM), which uses a tree-based ensemble algorithm and requires no prior knowledge of genetic effects. LightGBM has outperformed traditional models in certain trait predictions [34]. Other models, such as the kinship-adjusted multiple-loci linear mixed model (KAML), incorporate prior biological knowledge to improve accuracy. KAML enhances prediction efficiency by leveraging weighted kinship information from genome-wide association studies (GWAS) [36].

To capture the complex, nonlinear genetic architecture underlying complex traits and incorporate prior biological information, deep learning (DL) has become one of the most promising methods in genomic selection [37-40]. Recent advances in functional genomics have enabled the integration of multi-omics data, enhancing the interpretability of DL models [41-44]. The underlying intuition is that in living organisms, genetic information is transmitted from genotype to final phenotype through multiple levels of intermediate biological processes, including epigenetic modification, transcription, and translation [45]. By structuring DL models to reflect these biological processes, researchers can create biologically meaningful architectures that enhance interpretability. Integrating multi-omics data enables backward tracing of DL neurons to identify specific biological entities—such as cis-regulatory elements and gene networks—that drive trait formation. This, in turn, helps prioritize potential causal variants and could improve GS performance [46-51]. While

DL models integrating multi-omics data have been employed in predicting polygenic risk scores of human traits [47, 52-54], their application in agricultural species remains limited, mainly due to the financial and logistical challenges of acquiring multi-omics data for individual animals or plants. To address this, models that do not require individual-level multi-omics data have been developed [55-57]. However, these studies often rely on pre-existing functional annotations, such as GWAS or pathway data. Notably, functional annotation resources for agricultural species are relatively limited compared to those available for humans, highlighting the potential of computational methods to complement functional annotations using existing data. Another obstacle is the lack of a comprehensive understanding of the transmission flow from genetic variants to gene networks. Despite ongoing advancements in experimental technologies, the biological links between causal variants and their target genes remain difficult to validate under strict conditions. Several previous studies have contributed to expanding the comprehensiveness of functional annotations [58-60] and the regulatory networks of hub genes [61, 62].

Here, we introduce DeepAnnotation, a novel, interpretable DL-based genomic selection model that integrates comprehensive species- and tissue-specific functional annotations—without requiring individual-level data—to predict phenotypes. Unlike traditional "black box" DL models, DeepAnnotation aligns multi-omics biological annotations with sequential network layers, reflecting the flow of genetic information from DNA. This design offers advantages in both phenotype prediction accuracy and model interpretability. To demonstrate its effectiveness, we applied DeepAnnotation to predict pork production traits, where it outperformed established methods—including rrBLUP, KAML, LightGBM, BLUP, BayesR, MBLUP, and BayesRC—in

terms of prediction accuracy, computational efficiency, and top-performing individual selection. Additionally, DeepAnnotation is highly interpretable, providing valuable insights into the potential molecular mechanisms underlying complex traits. DeepAnnotation is freely available via GitHub and Docker, offering a powerful tool for data-driven breeding of complex traits.

Analyses

Interpretable deep learning genomic selection model

To integrate functional annotations into phenotype prediction, we developed a novel genomic selection model, DeepAnnotation (Fig. 1). This interpretable deep learning (DL) framework incorporates prior biological knowledge, specifically the natural flow of regulatory information from genotype to intermediate molecular phenotypes. Unlike traditional models that rely on low-density microarray data as genotype input, DeepAnnotation utilizes whole-genome sequencing data, which captures the full spectrum of SNPs present within a population. This key distinction enables a comprehensive use of both coding and non-coding functional annotations during model training.

The DeepAnnotation pipeline consists of three primary steps. First, we constructed comprehensive functional annotations by assigning cis-regulatory elements to their target genes and predicting their potential biological functions. To address gaps in gene function knowledge, we integrated transcriptomic data and applied the easyMF method to enhance and refine these gene function annotations [63]. Through matrix factorization, we identified regulatory module metaterms, which represent groups of genes that cooperate in coordinated biological processes. Second, we employed DeepSEA to predict chromatin accessibility changes resulting from non-coding cis-regulatory SNPs

[64] and used RNAfold (version 1.8.5) to predict RNA secondary structure alterations induced by coding SNPs [65]. These tools provided valuable insights into the impact of SNPs on regulatory regions as well as transcript and protein abundance. Finally, we integrated functional network annotations and SNP impact predictions into a deep neural network (DNN), forming the DeepAnnotation model. By leveraging functional annotations, DeepAnnotation significantly improves the accuracy and performance of genomic selection. Additionally, its biologically meaningful architecture enables us to trace genetic variations back to their phenotypic consequences, unraveling the complex genetic mechanisms underlying trait variation (Supplementary File 1).

The detailed architecture of the DeepAnnotation model consists of 7 layers, each dedicated to processing distinct types of omics data (Fig. 1 and Supplementary File 1). These layers encompass genotype data, epigenomic features, RNA secondary structure data, transcriptomic profiles, gene function annotations, regulatory module metaterms, and additional high-order features, represented by two hidden layers. Beginning with genotype data as the foundational layer, subsequent layers are added to enrich the model's understanding of intermediate biological processes between genotype and phenotype. These subsequent layers consist of various elements that enhance our understanding of genetic regulation. First, the chromatin accessibility and RNA secondary structure layers provide critical insights into how non-coding and coding SNPs influence gene regulation. Next, the gene function annotation layer elucidates the functional roles of individual genes, while the regulatory module metaterms layer captures higher-order regulatory interactions, identifying coordinated patterns of gene expression across multiple genes and regions. The two hidden layers aggregate the information from previous layers, allowing the model to summarize and integrate complex

functional annotations. Finally, the top layer predicts the phenotype, consolidating the model's ability to both predict complex traits and provide biological insights.

To optimize hyperparameters within the constraints of available computational resources, we employed a 2-fold cross-validation (CV) experiment to determine the optimal hyperparameters settings (Methods) (Supplementary Table S1). To evaluate the final prediction performance, we conducted a 5-fold cross-validation using these optimal hyperparameters, ensuring a robust assessment of model accuracy and generalizability (Methods).

Prediction performance of DeepAnnotation

In preparing functional annotation data for training DeepAnnotation, we utilized RNAfold, DeepSEA, and easyMF models. These tools allowed us to enrich functional annotations beyond genotype, encompassing aspects such as RNA secondary structure, chromatin accessibility, gene function, and gene networks (metaterm regulatory modules). Specifically, the input data for training the DeepAnnotation model was organized into four distinct components (Methods):

1. Genotype data: 590,342 potential non-coding cis-regulatory SNPs within open chromatin regions and 65,706 coding SNPs.
2. SNP functional impacts: Predicted by DeepSEA for non-coding SNPs and RNAfold for coding SNPs to assess their potential biological effects.
3. Gene annotations: Derived from 4,111 annotated genes located in conserved regions, encompassing 227 GO and KEGG terms, all with AUC value exceeding 0.9, as determined by easyMF.

4. Metaterm regulatory modules: 31 metaterms extracted from a pool of 27,797 genes, excluding the 4,111 genes from (3), spread across 14,996 terms using easyMF.

To identify the optimal hyperparameter combinations based on these functional annotations, we employed a rigorous evaluation process using Pearson correlation coefficient (PCC) scores between predicted and observed lean meat percentage at 100 kg (LMP) trait measurements within a 2-fold CV framework (Methods). We calculated the mean and median PCCs from a pool of 500 hyperparameter combinations, employing a voting strategy to determine the best hyperparameters. This strategy considered the mean and median PCCs obtained from models utilizing different levels of input annotation:

Level 1 (Genotype): Only genotype data.

Level 2 (SNPAnnotation): genotype + SNP functional impact.

Level 3 (Function): genotype + SNP functional impact + gene annotation.

Level 4 (Network): genotype + SNP functional impact + gene annotation + metaterm regulatory module.

For example, the best learning rates based on mean PCC were 0.1, 0.1, 0.001, and 0.1 for Level 1, 2, 3, and 4, respectively, while the best median PCC-based rates were 0.1, 0.1, 0.01, and 0.1 for Level 1, 2, 3, and 4, respectively (Fig. 2A). The voting results indicated a preference for a learning rate of 0.1 with 6 votes for this value, while 1 vote for 0.01 and 0.001, as well as 0 vote for 0.0001 and 0.00001 (Fig. 2A). In cases where multiple parameter values received the same number of votes, we used performance-based selection to determine the most favorable parameter. For instance, both

momentum values of 0.9 and 0.95 received 4 votes, but 0.9 yielded a higher average PCC (0.187 vs 0.139) and was chosen as the optimal value. By employing this strategy, the final optimal hyperparameter settings for DeepAnnotation were: learning rate of 0.1, base feature unit of 110, regularizer norm of L2, regularizer rate of 0.01, momentum of 0.9, dropout rate of 0.1, and a feature unit ratio of 1:2:4:5:6:7 (Fig. 2A and Supplementary Table S1).

We then compared the prediction performance of DeepAnnotation with other established models—rrBLUP, KAML, LightGBM, BLUP, BayesR, MBLUP, and BayesRC—using a rigorous 5-fold CV approach. For models that predict phenotypes directly from genotype without biological prior functional information, the overall PCC scores from all CV results were: 0.408 (rrBLUP), 0.288 (LightGBM), 0.393 (KAML), 0.398 (BLUP), 0.398 (BayesR) and 0.234 (Genotype, Level 1 for DeepAnnotation with only genotype data) (Fig. 2B). For models incorporating biological prior functional information, the PCC scores were: 0.392 (MBLUP), 0.383 (BayesRC), 0.434 (SNPAnnotation, Level 2), 0.459 (Function, Level 3), and 0.481 (Network, Level 4) (Fig. 2B). These results demonstrate that integrating functional annotations significantly improves prediction performance. DeepAnnotation showed a relative improvement of 6.4% to 67.3% over other models, depending on level of functional information used (Fig. 2B). The same conclusion could be reached under the distribution of 5 CV results: averaged PCC scores of 0.408, 0.293, 0.394, 0.405, 0.397, and 0.254 for rrBLUP, LightGBM, KAML, BLUP, BayesR, and Genotype without biological prior functional information. Averaged PCC scores of 0.400, 0.382, 0.447, 0.473, and 0.494 for MBLUP, BayesRC, SNPAnnotation, Function, and Network with biological prior functional information (Fig. 2B). Notably, DeepAnnotation with functional annotations outperformed all other models with

statistical significance (*P*-value < 0.05, paired t-test based on 5 CV results), except for SNPAnnotation (Level 2) when compared to rrBLUP (*P*-value=0.067) and BLUP (*P*-value=0.06) models.

To further investigate the advantage of DeepAnnotation, we analyzed the relative efficiency (RE) values between predicted and observed phenotypes for top-ranked individuals (top 1% to top 10%) (Fig. 2C). DeepAnnotation consistently outperformed other models in identifying the highest-ranking individuals (paired t-test, *P*-value < 0.05) with biological prior functional information. While DeepAnnotation (does not account for hyperparameter optimization) trained faster than Bayesian-based models (*P*-value < 0.05), it was slightly slower than BLUP-based models which are compiled on C frameworks (Fig. 2D). However, the training times for rrBLUP, LightGBM, KAML, and DeepAnnotation were similar, taking only a few hours. Overall, these findings highlight DeepAnnotation's flexibility and efficiency, incorporating multi-omics data to progressively improve phenotype prediction accuracy, particularly in identifying top-ranking candidates.

To assess the robustness of DeepAnnotation, we evaluated its performance on an independent test set of LMP (Fig. 3) (Methods). Using the Network model trained with all functional annotations, we tracked the training losses at each step, and then calculated the mean loss of each training process. We determined the optimal training step by counting the number of steps after which the loss was not going down anymore. For example, during optimization, we observed that the minimal training loss remained constant at 8.799624 starting from Step 603 and continued for the subsequent 103 steps (Fig. 3A). Therefore, a 5-fold CV experiment was then conducted again with 603 training steps

to predict phenotypes, and RE values for top-ranked individuals were averaged to provide a robust performance assessment (Fig. 3B). As anticipated, DeepAnnotation exhibited superior performance compared to other models, as evidenced by its significant (paired t-test, P-value < 0.05) higher RE scores on top 1~20 ranked samples (Fig. 3B), demonstrating its capability in accurately identifying superior individuals. Taken together, these findings highlight the broad utility and great robustness of DeepAnnotation in accurately identifying individuals with exceptional performance.

Biological interpretability of DeepAnnotation

To demonstrate the biological interpretability of DeepAnnotation, which means providing insights into the genetic underpinnings of LMP traits, offering a proof-of-concept regarding the model's interpretability. Focusing on the optimal validation status of Network model (Fig. 3A), we exemplified to exploit the proposed backward tracing strategy (Fig. 4A, Supplementary Fig. S1 and Supplementary File 1), by extracting the 5 weights of all nodes trained at training Step 382 (Fig. 3A) with all functional annotations through 5-fold CV experiment and calculating their significance using a meta-strategy with multiple testing correction via the 'RobustRankAggreg' R package [66]. A total of 8 metaterms, 4,264 terms, 950 genes, 5,290 cis-regulatory elements, 4,804 non-coding SNPs, 567 RNA secondary structures, and 484 coding SNPs showed significant (adjusted P-value < 1.0e-02) contributions to LMP.

Among the significant metaterms, metaterm 8 (adjusted P-value = 1.8e-04) appeared to be acting as a critical co-regulatory module regulating skeletal muscle development through epigenetic regulation. The signature functional term for this metaterm was GO:0006346 (adjusted P-value =

0.049) (Fig. 4B and Supplementary Table S2). Previous studies have highlighted the importance of DNA methylation as a key epigenetic modification in skeletal muscle development [67, 68], aligning with the biological process denoted by GO:0006346 (BP domain, DNA methylation-dependent heterochromatin assembly).

Leveraging the comprehensive functional annotation resource and node weights estimated during the training process of DeepAnnotation, we prioritized potential causal markers within metaterm 8. From pig GO database, only HDAC1 (ENSSSCG00000003613, histone deacetylase 1, *P*-value = 2.7e-04) was identified as a potential causal gene annotated in GO:0006346. Histone deacetylases (HDACs) play crucial roles in regulating skeletal muscle metabolism, motor adaptation, and exercise capacity [69, 70]. Previous study has shown that HDAC1 is sufficient to activate FoxO and induce skeletal muscle fiber atrophy [70], pointing to its key regulatory role in skeletal muscle development. Further functional annotation using easyMF predicted 49 candidate genes with similar functions to HDAC1, with significant functional similarity (*P*-value = 9.2e-22) compared to other genes (Fig. 4C and Supplementary Table S3).

Among these 49 genes, 6 genes were identified to significantly (*P*-value < 0.01) contribute to LMP through a back-tracing strategy (Supplementary Table S3). To identify potential causal SNPs that regulate these 6 genes within +/-1Mb regions, we calculated their significant levels based on the back-tracing strategy. A total of three non-coding SNPs were identified as statistically significant (*P*-value < 0.05) and proposed as cis-regulatory variants influencing gene expression related to skeletal muscle development (Supplementary Table S3). However, the SNP chr4:95309469 was not

predicted to reside within a peak region by the DeepSEA model. The predicted ATAC signals for this variant, with a threshold of 0.147, were 0.117 for reference allele and 0.106 for alternative allele, failing to meet the significance criteria. Consequently, the remaining two non-coding SNPs were prioritized as potential critical cis-regulatory variants with stronger evidence for functional relevance. The first SNP, chr10:23833431 (adjusted *P*-value = 5.6e-03), is located 903,403bp upstream of KDM5B (ENSSSCG00000010928, lysine demethylase 5B, adjusted *P*-value = 3.8e-06), a gene known to play a key role in skeletal muscle differentiation [71]. This SNP was located in an ATAC-Seq peak region of skeletal muscle tissue with a predicted score of 0.564 from DeepSEA model (threshold was 0.147), suggesting its regulatory role in KDM5B expression. Studies have indicated that KDM5B was highly expressed in human skeletal muscle tissue and regulated myoblast differentiation and muscle development [71-73]. Moreover, in a mouse strain with KMD5B knockout, increased phosphorylation of proteins involved in insulin signaling was observed in skeletal muscles, suggesting a role for KDM5B in regulating muscle metabolism [74, 75]. Furthermore, studies of purebred and Duroc-crossbred Iberian pigs, which exhibited significant differences in muscle growth, indicated that KDM5B may regulate gene expression in muscle [76]. These findings suggested a potential role for KDM5B in skeletal muscle development. Additionally, dual-luciferase reporter assays confirmed that chr10:23833431 was located in an enhancer (student t-test, *P*-value<0.001) (Fig. 4D), and its two alleles displayed differential enhancer activity (student t-test, *P*-value = 0.003687) (Fig. 4D).

The second SNP, chr4:95182500 (adjusted *P*-value = 3.6e-02), was located 38,367bp upstream of UBE2Q1 (ENSSSCG00000006544, ubiquitin conjugating enzyme E2 Q1, adjusted *P*-value = 4.9e-

04). This SNP resides within an ATAC-Seq peak in skeletal muscle tissue with a predicted score of 0.168 from DeepSEA model (threshold was 0.147), suggesting a potential regulatory role in UBE2Q1 expression. Previous studies have found that UBE2Q1 expression was upregulated in the muscles of male mice with spinal and bulbar muscular atrophy [77], suggesting its involvement in muscle atrophy. Further studies revealed that UBE2Q1 interacts with miR-27a, which inhibits the fast myofiber phenotype [78], suggesting the vital role of UBE2Q1 in skeletal muscle development. Dual-luciferase reporter assays results further validated that chr4:95182500 was located in an enhancer (student t-test, *P*-value<0.001), with its two alleles exhibiting differential enhancer activity (student t-test, *P*-value = 0.0006329) (Fig. 4D).

Taken together, through the back-tracing approach of DeepAnnotation, we proposed two potential molecular mechanisms involving non-coding cis-regulatory SNPs that may influence pig LMP. This discovery emphasizes the significant potential of DeepAnnotation to elucidate the flow of genetic information from genotype to phenotype by integrating diverse functional annotations (Fig. 4D).

Discussion

We introduced DeepAnnotation, an innovative framework that predicts phenotypes from genotypes by integrating a broad range of functional annotations. To the best of our knowledge, DeepAnnotation is the first interpretable deep learning approach leveraging comprehensive multi-omics functional annotation in livestock. Beyond accelerating the selection of exceptional individuals in genetic improvement, this method also provides lights on the genetic basis underlying critical economic traits. DeepAnnotation is now publicly freely available through GitHub [79] and

Docker [80] repository, extending its capabilities to facilitate big-data-driven breeding of complex traits.

With significant advancements in high-density genotyping platforms, the number of genomic loci tested for each individual has surged from tens of thousands to tens of millions of SNPs. Functional annotation of these genomic loci has benefited from the continuous development of high-throughput experimental methods and sophisticated computational algorithms. Together, these technological breakthroughs hold great promise for improving genomic selection prediction accuracy by accurately incorporating biologically functional variants into prediction models. To explore this potential, we conducted an experiment to predict phenotypes using different sets of SNPs (Supplementary Fig. S2) using rrBLUP model:

1. 11,633,164 SNPs (whole genome) from a published pig population genetics dataset [81].
2. 32,451 SNPs from the GeneSeek porcine 50K SNP array.
3. 296,537 SNPs located in Duroc Muscle ATAC-Seq peak regions.
4. 900,965 SNPs predicted to be located in skeletal muscle open chromatin regions by DeepSEA.

The results showed that predicting phenotypes from whole-genome SNPs was slightly more accurate than using only array SNPs (mean PCC: 0.409 vs 0.405). This suggests that valuable information exists within the whole genome that is not captured by array-based approaches. However, using a more comprehensive set of SNPs also introduces the challenge of incorporating potentially irrelevant information, which could contribute noise during model training. To mitigate this, functional annotations, such as the chromatin accessibility, can be used to prioritize cis-

regulatory variants. This approach improved prediction performance to a mean PCC of 0.413 (Supplementary Fig. S2). The growing adoption of functional genomics in livestock genetics has led to the incorporation of various omics data into refined databases or web servers, such as FAANG [82], ISwine [46], GWAS Atlas [83], IAnimal [84] and PigBiobank [85]. The integration of such rich data will undoubtedly strengthen genomic selection accuracy for a wide range of farm animals.

However, functional genomics data remains incomplete, and in cases where data such as open chromatin regions are not readily available, computational models like DeepSEA offer an effective strategy to complement these gaps. This is reflected by the increase in prediction PCC to 0.415 when DeepSEA-predicted SNPs were included (Supplementary Fig. S2). Similarly, the incorporation of gene function based on co-expression patterns was instrumental, especially since only 0.017% (2 of 11,941 protein-coding genes annotated with evidence code IDA and TAS) of pig protein-coding genes have robust GO annotations. By using easyMF model, we were able to complement gene functions, greatly enhancing the construction of multi-level functional annotations within DeepAnnotation.

Ongoing debates persist regarding the performance of genomic selection models, comparing traditional statistical algorithms with newer DL-based algorithms [11, 39, 86]. Furthermore, the relative advantages of employing single genomic data versus multi-omics data remain an active topic of discussion [37, 51, 87]. In livestock, the development of complex traits involves intricate biological regulatory processes, where genetic information flows from DNA through intermediate molecular phenotypes before manifesting as economically relevant traits [88]. Capturing this

nonlinear information flow through intermediate molecular phenotypes can potentially outperform linear statistical models that rely solely on genotype data [51]. Our findings align with this, as the prediction performance of DeepAnnotation gradually improved, significantly outperforming other genomic selection models (Fig. 2B-2C). Notably, DeepAnnotation demonstrated superior performance in selecting top-ranked individuals (top 1% and top 10%) (Fig. 3B-3C), highlighting the advantage of integrating deep learning with multi-omics data. This integration enables the capture of intricate genetic interactions that might be overlooked by relying solely on single omics (genotype) and linear models.

To further validate the superior performance of DeepAnnotation, we optimized hyperparameters for two additional pork production traits: loin muscle depth at 100 kg (LMD) and back fat thickness at 100 kg (BF) (Fig. 5A and Supplementary Table S1). Our analyses consistently showed the advantage of DeepAnnotation in prediction accuracy compared with other models (Fig. 5B-5C). The relative improvement in prediction accuracy was substantial, ranging from 19.5% to 120.0% for LMD (Fig. 5B) and 33.1% to 71.0% for BF (Fig. 5C) based on the overall PCC scores when taking all CV results together, depending on the level of functional information used. These results underscore the importance of considering different combinations of hyperparameters for optimizing performance in diverse scenarios.

Beyond the superior prediction performance, we also focused on the biological interpretability of DeepAnnotation model, a crucial aspect in deep learning application [34, 41]. Unlike the traditional ‘black box’ nature of neural networks, DeepAnnotation’s layers were designed with biologically

interpretable identities to mimic the flow of genetic information (Fig. 1). This architecture facilitates the identification of trait-relevant SNPs, genes and pathways through backpropagation from the phenotype layer to the genotype layer, enabling the proposal of potential molecular mechanisms influencing traits. This capability was demonstrated by fine-mapping two potential causal non-coding SNPs implicated in LMP formation, whose cis-regulatory functions were experimentally validated (Fig. 4D), further underscoring the model's interpretability.

Identifying causal variants remains a significant challenge. To further evaluate the statistically significant variants identified by DeepAnnotation, we estimated both the heritability and genomic prediction accuracy for the real LMP trait. We compared the Network and Genotype models, representing DeepAnnotation with and without functional annotations, respectively. During 5-fold cross-validation on 1,700 training samples, validation losses were monitored to determine the optimal training epoch, which occurred at epoch 282 for the Genotype model (Fig. 6A). For comparison, we selected rrBLUP, BLUP, and BayesR as baseline models based on their balance between performance and runtime (Fig. 2B and 2D). The heritability explained by statistically significant SNPs (*P*-value < 0.05) was 0.378, 0.383, 0.387, 0.428, 0.487, 0.495, and 0.503 for Genotype, BLUP, Network, BayesR, rrBLUP, metaG (a meta-analysis combining rrBLUP, BLUP, BayesR, and Genotype), and metaN (combining Network with other models), respectively (Fig. 6B). While DeepAnnotation alone performed slightly worse than the baseline models, its integration achieved higher heritability with metaN performed the best, highlighting the complementarity between DeepAnnotation and BLUP- or Bayesian-based approaches, and emphasizing the importance of accurate functional annotations. Moreover, the 11,084 potential causal variants (set I)

identified by DeepAnnotation demonstrated significantly higher GWAS power compared to the remaining SNPs (t-test, *P*-value = 3.89e-4). In terms of genomic prediction accuracy (measured by PCC) (Fig. 6C), DeepAnnotation prioritized SNPs (with *P*-value < 0.01 from the backtracking strategy) outperformed those selected solely from GWAS at stringent thresholds (*P*-value < 1.0e-6 [set III], 1.0e-5 [set IV], 1.0e-4 [set V]), as well as randomly selected SNPs (set VI). Although the SNPs located within 1Mb (set II) of the DeepAnnotation identified variants achieved the highest prediction accuracy, DeepAnnotation demonstrated greater enrichment of informative SNPs—calculated as the ratio of prediction accuracy (PCC) to SNP set size—than set II, set IV, set V, and set VI, but slightly less than the highly stringent GWAS set III (Fig. 6C). These findings indicated that, although the current backward tracing strategy (Fig. 4A, Supplementary Fig. S1 and Supplementary File 1) may miss certain key loci, DeepAnnotation achieved a higher enrichment of informative SNPs than standard SNP selection strategies, with the exception of the most stringent GWAS threshold, underscoring the potential of DeepAnnotation to uncover novel genetic insights. Future work should focus on improving the backward tracing strategy to identify additional informative SNPs, genes, and gene modules, thereby further elucidating the genetic architecture of LMP and other economically important traits.

Despite the superior performance and the broad application of DeepAnnotation, several limitations in current study warrant consideration. First, the dataset used for training (1,700 samples) and independent validation (240 samples) in this study, while informative, is relatively small for deep learning applications. Deep learning models typically require large-scale datasets to achieve robust training and validation, particularly when the number of SNPs far exceeds the number of samples.

The limited sample size in this study may hinder the accurate estimation of molecular marker effects and the generalizability of the model, as well as the causal variants identification. Future work should explore the performance of DeepAnnotation using significantly larger training and external validation datasets to better assess its scalability and robustness, as well as the performance of causal variants fine-mapping. Second, the influence of linkage disequilibrium (LD) was not accounted for when identifying potential causal SNPs. In this study, potential causal markers were detected through a back-tracing strategy combined with a meta-strategy and multiple testing correlation, without further adjustments for LD. Although dual-luciferase reporter assays provided experimental evidence supporting the cis-regulatory function of the identified variants, this approach alone is insufficient to fully validate causality. Future studies should incorporate LD adjustments and employ direct gene knockout experiment to further investigate the functional impact of the identified SNPs and confirm their causal roles.

Potential implications

We introduce DeepAnnotation, a novel genomic selection approach, which is publicly available through GitHub and Docker image. This framework leverages comprehensive multi-omics functional annotations to predict phenotypes. Comparative evaluations against seven well-established models, namely rrBLUP, LightGBM, KAML, BLUP, BayesR, MBLUP, and BayesRC, highlighted DeepAnnotation's superior performance with computational efficiency. The interpretable architecture of DeepAnnotation addresses the typical 'black box' nature of deep learning models. By providing insight into the underlying genetic mechanisms, it facilitates the fine-mapping of potential causal SNPs and uncovers crucial information about the genetic basis of

complex traits. This interpretability is particularly valuable for understanding the biological processes that contribute to economically important traits in livestock. It can aid in the identification of key genetic markers and regulatory mechanisms, accelerating the development of breeding strategies and improving the efficiency of livestock breeding programs.

Methods

Genomic selection models

Best linear unbiased prediction (BLUP) model

Given the genotype matrix $\boldsymbol{Z}(n \times p$; n individuals and p markers) and the corresponding vector of phenotype values $\boldsymbol{y}(n \times 1)$**,** the general mathematical statistical model for BLUP can be defined as the following standard linear regression formula:

$$\boldsymbol{y} = \boldsymbol{\mu} + \boldsymbol{Z}\boldsymbol{g} + \boldsymbol{\varepsilon}$$

Where, $\boldsymbol{\mu}$ represents the vector of overall mean observed phenotype values of n individuals, $\boldsymbol{Z}$ is a matrix of SNP genotypes (e.g., [aa, Aa, AA] = [0,1,2] for biallelic single nucleotide polymorphisms), $\boldsymbol{g} \sim N(0, \boldsymbol{K}\sigma_g^2)$ represents the vector of SNP effects, $\boldsymbol{\varepsilon} \sim N(0, I\sigma_\varepsilon^2)$ **r**epresents the vector of random residual effects. Here, σ_ε^2 is the variance of residual effects, σ_g^2 is the variance of genetic effects, $\boldsymbol{I}$ is an identity matrix with n rows and n columns, $\boldsymbol{K}$ is a genomic similarity matrix (GSM) specifying the correlation structure of $\boldsymbol{g}$ and could be calculated by $\boldsymbol{K} = \boldsymbol{Z}\boldsymbol{Z}'/p$ with p represents the number of SNPs, $\boldsymbol{Z}'$ represents the transpose of $\boldsymbol{Z}$**.** Finally, we implemented BLUP model by setting '--reml-pred-rand --reml-est-fix --blup-snp' with GCTA software [89].

Ridge regression best linear unbiased prediction (rrBLUP) model

The rrBLUP model is equivalent to BLUP in the context of mixed models [90], which could be defined by the following formula:

$$\boldsymbol{y} = \boldsymbol{WGu} + \boldsymbol{\varepsilon}$$

Where, $\boldsymbol{u} \sim N(0, \boldsymbol{I}\sigma_u^2)$ represents the SNP effects vector, $\boldsymbol{G}$ represents the genotype matrix, $\boldsymbol{W}$ represents the designed matrix, $\boldsymbol{\varepsilon}$ represents the random residual effects, $\lambda = \sigma_e^2/\sigma_{\mathrm{u}}^2$ represents the ridge parameter. Compared with ordinary regression, for which the number of markers cannot exceed the number of observations. Finally, we implemented rrBLUP model with 'mixed.solve' function in R package 'rrBLUP' [18].

Best linear unbiased prediction with multiple random effects (MultiBLUP) model

The basic statistical mathematical model underlying MultiBLUP is the same with BLUP, except for incorporating multiple random effects $\boldsymbol{g} = [\boldsymbol{g}^1, \boldsymbol{g}^2, \dots, \boldsymbol{g}^m]$ for different classes of SNPs [33]. The pre-defined class $\mathrm{c} \in [\mathrm{c}_1, \mathrm{c}_2, \dots, \mathrm{c}_\mathrm{m}]$ could be identified by the prior biological information. MultiBLUP extends BLUP model to include the genomic relation matrix specified by $\boldsymbol{K} = [\boldsymbol{K}^1, \boldsymbol{K}^2, \dots, \boldsymbol{K}^m]$ and the corresponding variances $\sigma = [\sigma_1^2, \sigma_2^2, \dots, \sigma_\mathrm{m}^2]$:

$$\boldsymbol{y} = \sum_{i=1}^{m} \sum_{j=1}^{R_i} \boldsymbol{Z}_j^i \boldsymbol{g}_j^i + \boldsymbol{\varepsilon}$$

Where, each $\boldsymbol{K}^m = \boldsymbol{Z}^{\boldsymbol{m}}\boldsymbol{Z}^{\boldsymbol{m}\prime}/p_m$ is the modified form of a genotype matrix $\boldsymbol{Z}^{\boldsymbol{m}}$ corresponding to a class set of SNPs R_i of size p_m, $g_j^i \sim N(0, \sigma_i^2/p_i)$. The estimation of variance parameters $\sigma_1^2, \dots, \sigma_\mathrm{m}^2$ and σ_ε^2 could be achieved by maximizing the log likelihood:

$$-\frac{n}{2}\log(2\pi) - \frac{1}{2}\boldsymbol{y}'\boldsymbol{v}\boldsymbol{y} - \frac{\boldsymbol{1}}{\boldsymbol{2}}\log|\boldsymbol{v}|$$

Here, $\boldsymbol{v} = \sigma_1^2\boldsymbol{K}^1 + \cdots + \sigma_1^2\boldsymbol{K}^m + \sigma_\varepsilon^2\boldsymbol{I}$**.** Finally, we implemented MultiBLUP model by setting '--

autosome --make-grm-alg 1 --make-grm --reml –mgrm --reml-pred-rand --reml-est-fix --blup-snp ' with GCTA software [89].

Hierarchical Bayesian mixture (BayesR) model

BayesR is a Bayesian-based method for deriving the prediction equation that assumes SNP effects follow a series of normal distributions. The general mathematical statistical model for BayesR can be defined by the following formula:

$$\boldsymbol{y} = \mu + \boldsymbol{X\beta} + \boldsymbol{e}$$

Where, μ is the intercept that represents the general mean, $\boldsymbol{X}(n,p)$ represents the numerical genotype matrix, $\boldsymbol{\beta}(p,1)$ represents the vector of SNP effects, $\boldsymbol{e}(n,1)$ represents the vector of residuals with $e \sim N(0, \boldsymbol{I}\sigma_e^2)$**.** Briefly, the SNP effects are modeled by a mixture of four normal distributions with zero mean and the variances are fixed specified by:

$$p(\beta_j | \pi, \sigma_g^2) = \pi_1 \times N(0{,}0 \times \sigma_g^2) + \pi_2 \times N(0, 10^{-4} \times \sigma_g^2) + \pi_3 \times N(0, 10^{-3} \times \sigma_g^2) + \pi_4 \times N(0, 10^{-2} \times \sigma_g^2)$$

Here, σ_g^2 represents the total additive genetic variance, the mixing proportions $\boldsymbol{\pi} = [\pi_1, \pi_2, \pi_3, \pi_4]$ are drawn from a Dirichlet distribution with parameter $= (1{,}1{,}1{,}1)$. The constant allocations values $(0, 10^{-4}, 10^{-3}, 10^{-2})$ imply the SNPs are assigned to four different effect size classes: null, small, medium and large, corresponding respectively to 0%, 0.01%, 0.1%, and 1% of σ_g^2**.** Finally, we implemented BayesR by setting '-burnin 5000 -numit 10000 -seed 0' with 'bayesR' software [27].

Bayesian genomic prediction with disjoint annotations (BayesRC) model

The central statistical linear model of BayesRC is the same with BayesR, the main difference between them is that BayesRC incorporates an independent biological prior information to allocate each SNP to a specific class $\boldsymbol{C}$, given the constraint condition that $|\boldsymbol{C}_j| = 1$ for the j-th SNP [29]. The SNP effects for each class $c \in [c_1, c_2, \dots, c_m]$ are defined by the following formula:

$$p(\beta_j | \pi, \sigma_g^2, \boldsymbol{C}_j = c) = \pi_{1,c} \times N(0, 0 \times \sigma_g^2) + \pi_{2,c} \times N(0, 10^{-4} \times \sigma_g^2)$$

$$+\pi_{3,c} \times N(0, 10^{-3} \times \sigma_g^2) + \pi_{4,c} \times N(0, 10^{-2} \times \sigma_g^2)$$

Where, $\sum_{k=1}^{4} \pi_{k,c} = 1$ for all c with m represents the number of independent classes. Here, π_c also drawn from a Dirichlet distribution with parameter $= (1,1,1,1)$. Finally, we implemented BayesR by setting '-burnin 5000 -numit 10000 -seed 0 -ncat 5' with 'bayesRCO' software [32].

Light gradient boosting machine (LightGBM) model

LightGBM is an ensemble model of gradient boosting decision trees (GBDT) [34]. For phenotype prediction from genotype matrix, GBDT uses decision trees to learn a function from the input space of numerical genotype matrix $\mathcal{X}^p$ to the gradient space $\mathcal{H}$ [91]. For GBDT, for training dataset O on a fixed node of the decision tree, the information variance gain of splitting feature j at point d for this node is defined by the following formula:

$$V_{j|O}(d) = \frac{1}{n_O}\left(\frac{(\sum_{x_i \in O: x_{ij} \le d} h_i)^2}{n_{l|O}^j(d)} + \frac{(\sum_{x_i \in O: x_{ij} > d} h_i)^2}{n_{r|O}^j(d)}\right)$$

Where, $n_O = \sum I|x_i \in O|, n_{l|O}^j(d) = \sum I|x_i \in O: x_{ij} \le d| \ \ and \ \ n_{r|O}^j(d) = \sum I|x_i \in O: x_{ij} > d|$, h_i represent the negative gradients of the loss function with respect to the output of the model. Then the decision tree algorithm selects $d_j^* = argmax_d V_j(d)$ and calculates the largest gain $V_j(d_j^*)$. Lastly, the data are split according feature j^* at point d_{j*} into the left and right child nodes. Finally, we implemented LightGBM model with 'lgb.train' function by setting 'objective = regression,

metric = l2, num_threads = 5, learning_rate= 0.1' in R package 'lightgbm' [92].

Kinship-adjusted-multiple-loci (KAML) linear mixed model

KAML is a flexible model that extends linear mixed model by integrating pseudo QTNs as covariates and an optimized trait-specific random effect [36]. The general mathematical statistical model for KAML can be defined by the following formula:

$$\boldsymbol{y} = \boldsymbol{X}b + \boldsymbol{Q}q + \boldsymbol{Z}\beta^{*} + \boldsymbol{\varepsilon}$$

Where, b is a vector of the fixed covariates effects with the corresponding coefficient matrix $\boldsymbol{X}$, $\boldsymbol{Q} = [Q_1, Q_2, \dots, Q_k]$ represents the k covariates that are derived from a multiple regression model-based selection procedure, $\beta^{*} \sim N(0, \boldsymbol{K_w}\sigma_g^2)$ is a vector of random effects representing the individual genetic values, $\varepsilon \sim N(0, \boldsymbol{I}\sigma_e^2)$ is a vector of residual effects, σ_g^2 and σ_e^2 are the genetic variance and residual variance, $\boldsymbol{K_w}$ is a SNP-weighted kinship (genomic relation matrix) which can be formulated as follows:

$$K_{wij} = \frac{1}{m}\sum_{k=1}^{m} \frac{(M_{ik} - 2p_k)\xi_k(M_{jk} - 2p_k)}{2p_k(1 - p_k)}$$

Here, ξ_k is the weight of ***k***-th SNP, m is the number of SNP, M_{ik} is the numeric value of genotype matrix of ***k***-th SNP in ***i***-th individual, p is the frequency of the coded allele. The weight ξ_k could be derived from the following formula:

$$\xi_k|(\alpha, \gamma) \sim \begin{cases} 1; \ 1 - \gamma \\ 1 + log_{\alpha}P_{m\gamma} - log_{\alpha}P_k; \ \gamma \end{cases}$$

Here, P represents the ordered ***P***-values of all SNPs from GWAS result, α is the base value of logarithmic function, and γ is the percentage of top significant SNPs to be weighted. Finally, we implemented KAML model by setting 'bin.size=1000000,max.nQTN=TRUE,sample.num=2, crv.num=5, cpu=5' in R package 'kaml'.

Deep learning based genomic selection model with comprehensive functional annotations (DeepAnnotation)

The DeepAnnotation model is a deep learning-based approach for genomic selection, which integrates multiple types of prior biological knowledge derived from various omics data. In this study, we assigned 7 layers to represent different types of prior knowledge. The basic mathematical framework for each layer is defined as follows:

$$\boldsymbol{y} = f(\boldsymbol{wx} + \boldsymbol{b})$$

Where, $\boldsymbol{y}$ represents vector the output values of each layer, $f(\cdot)$ represents the active function, $\boldsymbol{w}$ represents the weights of markers, $\boldsymbol{x}$ represent the vector of input values, $\boldsymbol{b}$ represents the bias. The overall loss function was defined by the following formula:

$$loss = \frac{1}{n}\sum(y_{real} - y_{predict})^2 + 0.00001 \times \alpha_k \sum ||w||_k$$

Here, n represent the individual number, y_{real} represents the observed phenotype values, $y_{predict}$ represents the predicted phenotype values, 0.00001 represents the binding penalty coefficient, α_k represents the regularizer rate, $||w||_k$ represent the $\boldsymbol{k}$-th norm of all weights. Parameters in the DeepAnnotation were optimized by minimum $loss$ with the back propagation algorithm [93]. More detailed description of DeepAnnotation can be found at Supplementary File 1. Finally, we implemented DeepAnnotation model on a GPU (NVIDIA RTX A6000) with Python 'tensorflow' package.

Hyperparameters

DL model performance relies on the optimization of a large number of hyperparameters. A previous

study suggested that the performance of deep learning models does not significantly improve with more than 100 hyperparameter sets [94]. Instead of testing 93,600 hyperparameter sets, we used 500 randomly sampled hyperparameter combinations to reduce computation time while maintaining reasonable performance. Specifically, the hyperparameters included: learning rate, feature number, regularization algorithm and rate, batch normalization momentum, and dropout rate. The ranges of values used for each hyperparameter are detailed in Supplementary Table S1. Consideration the acceptable running time on the selection of optimal hyperparameter set, we did a 2-fold cross-validation experiment to select the best hyperparameter combination based on the Pearson correlation coefficient (PCC) score from those 500 hyperparameter pools. The hyperparameter optimization took a total of 409 hours, 26 minutes, and 51 seconds (approximately 17 days).

Genotype and phenotype data

For model training, we used a public dataset including whole-genome SNPs and three pork production traits (lean meat percentage at 100 kg [LMP], loin muscle depth at 100 kg [LMD], back fat thickness at 100 kg [BF]) as well as four other traits (total teat number [TTN], left teat number [LTN], right teat number [RTN], time spent to eat per day [TPD, min]) records of 2,802 Duroc boars [81]. After imputing the missing SNPs with Beagle (version 5.1) software [95], over 11.6 million (11,633,164) SNPs encoded with a [0, 1, 2] format corresponding to [AA, Aa, aa], where A is the major allele. After filtering out samples with missing phenotype values, 1,940 samples were remained. Among these 1,940 samples, 240 samples were randomly selected as independent test set and never used during the training process. Therefore, the remaining 1,700 samples were used to search for the best hyperparameters combination on LMP, LMD and BF through a 2-fold cross-

validation experiment, respectively. Subsequently, the prediction performance of 1,700 training samples was thoroughly exemplified evaluated on LMP trait through a 5-fold cross-validation experiment, and the prediction performance of 240 independent samples was evaluated from the model trained on those 1,700 samples. In addition, LMD and BF traits were also evaluated to support the results of prediction performance comparison. Finally, the PCCs of 240 independent samples were calculated on all seven traits by rrBLUP to evaluate the performance of different functional aspects of SNPs.

Comprehensive multi-omics functional annotation data processing

Epigenome data

The published epigenomic data of processed ATAC-seq was download under Gene Expression Omnibus (GEO) accession number GSE143288, including five pig tissues (muscle, liver, fat, spleen, and heart) from four breeds including Duroc, Enshi Black (ES), Large White (LW), and Meishan (MS) (Supplementary Table S4) [96]. We also downloaded the processed ATAC-seq (GSE158414) includes eight Yorkshire tissues (adipose, cerebellum, cortex, hypothalamus, liver, lung, muscle, spleen) (Supplementary Table S4) [97]. Furthermore, we merged the tissues from Large White and Yorkshire named Large White Yorkshire (LWY). We used ‘intersect’ command of BEDTools (version v2.25.0; RRID:SCR_006646) to define consistent peak regions from biological replicates, and used ‘merge’ command to merge the consistent peak regions from same tissue [98].

Transcriptome data

The sample information of transcriptomic data was download from ‘Expression Section’ of ISwine

website by searching ‘Duroc’ keyword [96]. Then, we downloaded the raw sequence data of 177 samples with a label of ‘Duroc’ or ‘duroc’ in Cultivar items that had been deposited in the NCBI. Sequence Read Archive (SRA) under the corresponding accession numbers (Supplementary Table S4). The raw sequencing reads were processed with trim_galore (version v0.6.7; RRID:SCR_011847) to remove adapters and trim low-quality bases, followed by mapping to reference genome Sscrofa11.1 assembly using HISAT2 (version v2.2.1; RRID:SCR_015530) [99], and gene expression quantification with featureCounts (version v2.0.1; RRID:SCR_012919) [100]. Finally, 31,908 genes from 177 samples with high-quality normalized transcripts per million expression levels were prepared for further analysis.

Transcripts annotation data

The gene annotation information in GTF format of pig reference genome Sscrofa11.1 was download from NCBI. We used ‘dplyr’ package in R to extract the gene position, and ‘intersect’ command of BEDTools to locate SNP in gene regions, and ‘window -w 1000000’ to annotate potential regulatory elements in within 1Mb of genes.

Gene functional annotation data

The GO annotation of pig reference genome Sscrofa11.1 was download from ‘BioMart’ section of Ensembl web server. Totally, there were 150,226 records covering 16,784 genes and 14,682 functional terms (referred to as terms). The pathway annotation of pig reference genome Sscrofa11.1 was downloaded from KEGG web server. Totally, there were 35,926 records covering 6,532 genes and 335 pathways (referred to as terms). We merged all these 186,152 records together covering

20,037 genes and 15,017 terms.

Conserved regulatory elements

To incorporate the conserved regulatory elements that are critical important towards understanding the genetics of complex traits, we download the functionally conserved annotation (including gene body, intergenic, and promoter coordinates) of pig from Kern's study [97]. We used 'intersect' command of BEDTools (version v2.25.0) to located those non-coding SNPs and genes that are within the conserved regulatory elements regions [98].

Comprehensive multi-omics functional annotations construction

Previous studies showed that SNPs located in coding regions could influence complex traits by altering RNA secondary structure or affecting nearby gene expression, while SNPs in non-coding regions through typically affect gene expression levels [101-104]. However, only a small subset (2 out of 11,941) of protein-coding genes have convincing GO annotations with evidence code IDA and TAS [105]. Therefore, to model the biological impact of both coding and non-coding SNPs in genomic selection, we employed RNAfold and DeepSEA to derive sequence-based scores for RNA minimum free energy (MFE) and chromatin accessibility, respectively. Additionally, to predict gene functions not annotated in existing GO and KEGG databases, we used easyMF to complement the functional annotations of all genes.

Minimum free energy calculation

In order to comprehensively represent the RNA secondary structure surrounding each SNP, we used

‘BSgenome’ package in R to extract 1,100 base pair (bp) of DNA sequence from the Sscrofa11.1 reference genome [64, 106]. This sequence was centered on the reference and alternative alleles of the SNP, extending +/-550 bp around each SNP. The ‘RNAfold’ program (ViennaRNA version 1.8.5) was then used with the parameters ‘-d2 -noLP’ to calculate the minimum free energy (MFE) for each sequence. Finally, the MFE values were then normalized to a range of [0, 1].

Cis-regulatory SNPs annotation

To predict the cis-regulatory effects of SNPs, we used the DeepSEA model, which learns the relationship between genomic sequence and chromatin accessibility [64]. The model’s accuracy in recognizing open chromatin regions was evaluated using the receiver operating characteristic curve (AUC) (Supplementary Fig. S3). Following the instruction of DeepSEA, during model training, the genome was split into 200-bp bins, and a bin was labeled as positive if it overlapped with an ATAC-Seq peak by more than 100 bp. These positive bins were then extended by 500 bp both upstream and downstream. The extended sequences were one-hot encoded into a 1000 × 4 binary matrix, with columns corresponding to A, G, C and T. Finally, we trained the DeepSEA model which takes the binary matrices as input and the chromatin accessibility as output. To annotate the cis-regulatory effects of SNPs, we also extracted the 1,000-bp sequences containing the reference and alternative alleles of each SNP, and predicted the chromatin accessibility difference of each allele pair by the trained DeepSEA model.

Gene functions annotation

To predict the biological functions annotation of gene, we employed the easyMF model, which

learns the associations between gene expression levels and functional terms [63]. The intuition behind easyMF is that genes sharing similar expression patterns across different cellular contexts are more likely to function in the same biological processes. The AUC was used to evaluate the accuracy of easyMF in prioritizing gene functions. Specifically, we used easyMF to decompose the gene expression matrix (containing 31,908 genes and 177 samples) into two low-dimensional matrices: an amplitude matrix (AM; genes in rows and metagenes in columns) and a pattern matrix (PM; metagenes in rows and samples in columns). Then, easyMF calculated the z-scores for each functional term by assessing the distribution of AM coefficients between genes with and without the functional term categorization [60]. The PCC was computed between the gene weights in the AM and the z-scores of each functional term, which were used to predict gene functions. The final completion of gene functions annotation was a gene-by-functional term matrix. To further refine the annotations, a second round of matrix decomposition was performed, generating “metaterms”, which are weighted combinations of functional terms. For each “metaterms”, the functional terms with dominant patterns represent its signatures (Refer to easyMF [63] for details).

Disjoint SNP classes based on biological prior functional annotations

For BayesRC and MBLUP models, disjoint SNP classes need to be defined based on biological prior functional annotations. For a fair comparison, the biological prior functional annotations used here were consistent with those employed in DeepAnnotation. Markers (SNPs and genes) were categorized into four broad categories based on the principle from MacLeod’s study [29], including: a) comprised 23,492 variants predicted to cause a non-synonymous coding change. b) comprised 632,556 variants except those in a). c) 4,111 genes (annotated in terms with AUC > 0.9) located in

conserved regulatory element regions. d) 823 signature genes for 31 used metaterms. Finally, five distinct SNP classes were defined based on those four broad categories, including: I) 4,185 SNPs from category a) that located within category c). II) 590 SNPs from category a) that located within category d). III) 280,399 SNPs from category b) located within +/-50kb category c). IV) 14,923 SNPs from category b) located within +/-50kb category d). V) 35,5951 other SNPs that were not in I), II), III) and IV).

Relative efficiency calculation

The relative efficiency (RE) is calculated based on the expected genetic gain when individuals are selected by genomic prediction [107] :

$$RE(k) = \frac{(\sum_{\alpha'} y_i)/N_{\alpha'} - (\sum_{Test} y_i)\,/N_{Test}}{(\sum_{\alpha} y_i)/N_{\alpha} - (\sum_{Test} y_i)\,/N_{Test}}$$

Here, α and α' are the groups of extreme individuals selected by the ranking of real observed or predicted phenotypic values, respectively; $k = N_{\alpha'} = N_{\alpha}$ are the numbers of individuals in each group; y_i is the real observed phenotypic value of i_{th} individual; and $(\sum_{Test} y_i)\,/N_{Test}$ represents the mean of the test group.

Heritability calculation

To calculate the heritability, we first extracted the weights of all SNPs trained by each model through 5-fold cross-validation. The significant levels of these SNPs were determined using a meta-strategy with multiple testing correction implemented in the 'RobustRankAggreg' R package. SNPs with an adjusted P-value < 0.05 were considered potential causal variants. For the real LMP trait, the heritability was estimated as the proportion of genetic variance explained by the predicted causal

variants, calculated using the BLUP model implemented in GCTA software ('--reml-pred-rand --reml-est-fix --blup-snp') [89].

Dual-luciferase reporter assay

The dual-luciferase reporter assay was conducted to assess the functional impact of the SNPs. The target fragment was inserted into the PGL4.23 vector in front of the promoter. During C2C12 cell culture and transfection, the culture medium consisted of 90% DMEM, 10% serum, and 1% penicillin/streptomycin. The cells were seeded in 24-well plates and transfected using jetPRIME transfection reagent. The transfection procedure was performed with 1.0 ug of plasmid per well (target plasmid to internal reference plasmid ratio: 5:1) and 2 ul transfection reagent with 200 ul jetPRIME. The target fragment was inserted into the PGL4.23 carrier, which expresses firefly luciferase, while the internal reference plasmid expressed sea cucumber luciferase. The two plasmids were introduced into the cells at a 5:1 ratio. After 24 hours of incubation, the cells were harvested for further analysis. The cells were lysed, and the fluorescence values for firefly luciferase and sea cucumber luciferase were measured using their respective reaction substrates. The relative luciferase activity was calculated as the ratio of the firefly fluorescence to the sea cucumber fluorescence, providing an internal control for normalization. The 101 bp target sequences for the reference (wild-type, WT) and alternative (MUT) alleles of the SNPs were as follows: chr10:23833431 (WT: G): GCAAAGGGGCTGCCCTGGCATGACCCTCGTCTTTGGGGACACTGGGACAAGGGCAAGGACATTGGAAAAAGCCTGGCCTCTGCCCAGGAACTAACTGGGGC, and alternative allele A (MUT: A): GCAAAGGGGCTGCCCTGGCATGACCCTCGTCTTTGGGGACACTGGGACAAAGGCAAGGACATTGGAAAAAGCCTGGCCTCTGCCCAGGAACT

AACTGGGGC. chr4:95182500 (WT: G): CGCAGCTGCAGAATTTCAGAGCTGAAAGAGACATTCAGAATTATTGAGTCGAATCTCCTCTCTTGGCAGATAAGAAATGAGACCAAGAACATGTACCTAAC, and alternative allele A (MUT: A): CGCAGCTGCAGAATTTCAGAGCTGAAAGAGACATTCAGAATTATTGAGTCAAATCTCCTCTCTTGGCAGATAAGAAATGAGACCAAGAACATGTACCTAAC. This method allowed us to assess the relative transcriptional activity of the WT and MUT alleles by comparing the luciferase signals.

Availability of source code and requirements

Source: GitHub repository [108]

Project name: DeepAnnotation

Project home page: https://github.com/mawenlong2016/DeepAnnotation

Operating system: Linux

Programming language: Python 3.6

Other requirements: conda, matplotlib, scikit-learn, numpy, tensorflow-gpu, framework-reproducibility

License: GPL -3.0

biotoolsID: deepannotation

RRID: SCR_026630

Source: Zenodo repository [109]

Project name: DeepAnnotation: A novel interpretable deep learning-based genomic selection model that integrates comprehensive functional annotations

Link: https://doi.org/10.5281/zenodo.8410693

License: CC0

Source: DOME annotations [110]

Project name: DeepAnnotation: A novel interpretable deep learning-based genomic selection model that integrates comprehensive functional annotations

Link: https://dome.dsw.elixir-europe.org/wizard/projects/4acf509b-3d08-4f6f-b4a9-46239ca43c6d

Data Availability

The large-scale whole-genome sequencing dataset of Duroc pig population can be directly downloaded from GigaDB [111]; The published epigenomic data of processed ATAC-seq can be directly downloaded under Gene Expression Omnibus (GEO) accession number GSE143288 [112]; The published epigenomic data of processed ATAC-seq of Yorkshire could be directly downloaded under GEO accession number GSE158414 [113]; The sample information of transcriptomic data can be directly obtained from 'Expression Section' of ISwine website [114] by searching 'Duroc' keyword, and then directly downloaded under the corresponding accession numbers from the NCBI Sequence Read Archive [115]; The gene annotation information in GTF format of pig reference genome Sscrofa11.1 can be directly downloaded from NCBI [116]; The GO annotation of pig reference genome Sscrofa11.1 can be directly downloaded from 'BioMart' section of Ensembl web server [117]; The pathway annotation of pig reference genome Sscrofa11.1 can be directly downloaded from KEGG web server [118]; The transformed genotype data containing 11,633,164 SNPs from 1,940 samples, the phenotype data containing three pork production traits from 1,940

samples, the comprehensive functional annotation data for Duroc prepared by RNAfold, DeepSEA, easyMF models, and the four types of input data, as well as several additional data files for training DeepAnnotation model are openly available in the DOI-assigning repository Zenodo [109, 119]. Machine learning annotations for DeepAnnotation have been deposited in the DOME registry [110]. The source code for building the comprehensive functional annotations using RNAfold, DeepSEA and easyMF is freely available through GitHub [120]. The source code of DeepAnnotation is freely available through GitHub [79] and Docker [80].

Abbreviations

DL: deep learning; GS: genomic selection; GP: genomic prediction; GEBV: genomic estimated breeding value; BLUP: best linear unbiased prediction; rrBLUP: ridge regression best linear unbiased prediction; MBLUP: best linear unbiased prediction with multiple random effects; BayesR: hierarchical Bayesian mixture; BayesRC: Bayesian genomic prediction with disjoint annotations; LightGBM: light gradient boosting machine; KAML: kinship-adjusted-multiple-loci linear mixed model; GWAS: genome-wide association studies; DNN: deep neural network; WGS: whole-genome sequencing; MFE: minimum free energy; bp: base pair; AUC: area under receiver operating characteristic curve; CBP: complex biological process; PCC: Pearson correlation coefficient; LMP: lean meat percentage at 100 kg; CV: cross-validation; BMP: bone morphogenetic protein; LMD: loin muscle depth at 100 kg; BF: back fat thickness at 100 kg; LWY: large white Yorkshire

Ethics approval and consent to participate

Not applicable.

Competing interests

The authors declare that they have no competing interests.

Funding

This work was supported by the Ministry of Science and Technology of the People´s Republic of China, National Key R&D Program of China, 2021YFF1000600 to Y Liu.

Author's Contributions

W.M. and Y.L. designed and supervised the project. W.M. and W.Z. performed phenotype prediction analysis. W.M. developed the DeepAnnotation software. W.Z., C.W. and B.L. processed multi-omics data. W.M. and Y.L. wrote the manuscript. S.Q., performed the dual-luciferase reporter assay experiment. All author(s) read and approved the final manuscript.

Figure legends

Figure 1: Schematic diagram of DeepAnnotation. The DeepAnnotation pipeline consist of three steps: Step1, assign coding and non-coding SNPs to cis-regulatory elements and genes; Step2, prepare functional annotations; Step3, construct a deep neural network that integrated comprehensive functional annotations for genomic selection. For each sample, the first layer receives genotype (a vector of coding and non-coding SNPs) as input. Besides direct features transfer along with the deep neural network (DNN), DeepAnnotation parallelly receives additional multi-level functional annotations: cis-regulatory effects (predicted chromatin accessibility of non-

coding SNPs) and RNA secondary structure effects (predicted minimum free energies of coding SNPs) were parallelly added at the second layer, gene function effects (predicted functional scores of genes for each functional term) at the third layer, regulatory module effects (calculated weights of functional terms for each metaterm) at the fourth layer. Subsequently, two hidden layers are used for summarizing the features extracted from the aforementioned layers. Finally, the last layer outputs the predicted phenotypes.

Figure 2: Prediction performance of DeepAnnotation through cross-validation compared with rrBLUP, LightGBM, KAML, BLUP, BayesR, MultiBLUP, BayesRC, and DeepAnnotation on LMP trait. (**A**) Pearson correlation coefficient scores of different hyperparameters combinations based on a 2-fold cross-validation (CV) experiment from DeepAnnotation using different types of functional annotation data. (**B**) PCC evaluation of different models based on 5-fold cross-validation experiment. In the "CV together" approach, each testing fold from the 5-fold cross-validation were consolidated before metric computation. On the other hand, in the "CV separate" approach, metrics were computed separately for each testing fraction of the 5-fold cross-validation. The paired t-test *P*-values of DeepAnnotation compared with other models were displayed on the black boxes with coral represents significance with *P*-value < 0.05. (**C**) Relative efficiency (RE) values between the predicted and observed phenotypic values of top-ranked samples from top 1% to top 10%. The paired t-test *P*-values were displayed on the black boxes with coral represents significance with *P*-value < 0.05. (**D**) Elapsed training time of different models for 5 CVs. Each column of vertical points represents one CV. For DeepAnnotation, the training times are not including the hyperparameter optimization.

Figure 3: Prediction performance of DeepAnnotation for independent test dataset of LMP. (**A**)

Training and validation status of DeepAnnotation for all functional annotations of each epoch based on the 5-fold cross-validation. (**B**) The distribution of RE scores of top 1~20 ranked samples under the distribution of 5 CV results.

Figure 4: Interpretability of DeepAnnotation allows for the fine-mapping of potential causal SNPs. (**A**) Schematic diagram of how DeepAnnotation exploited backward tracing to identify potential causal SNPs and their mediated causal genes and gene regulatory modules. (**B**) Metaterm 8 acts as a co-regulatory module that regulates skeletal muscle development through epigenetic regulation. (**C**) Potential causal genes in metaterm 8 and their functional properties compared with others. (**D**) DeepAnnotation pinpoints two experimentally validated non-coding enhancer-modulating SNPs and proposes how that might influence pork production traits through regulating the expression of genes relevant to skeletal muscle growth and development. The first SNP, chr10:23833431, is predicted to regulate the expression of KDM5B, while the second SNP, chr4:95182500, may influence the expression of NUBE2Q1. Both genes are implicated in myoblast differentiation and function, playing critical roles within the regulatory module governing skeletal muscle cell differentiation. These regulatory effects may ultimately influence skeletal muscle development and contribute to variations in the LMP trait. Supporting evidence from dual-luciferase reporter assays is shown in the bar charts on the left and right.

Figure 5: Prediction performance of DeepAnnotation in terms of robustness. (**A**) Optimized hyperparameters of DeepAnnotation on LMP, LMD, BF traits. PCC evaluation of different models based on 5-fold cross-validation experiment for (**B**) LMD and (**C**) BF. In the "CV together" approach, each testing fold from the 5-fold cross-validation were consolidated before metric computation. On the other hand, in the "CV separate" approach, metrics were computed separately for each testing

fraction of the 5-fold cross-validation. The paired t-test P-values of DeepAnnotation compared with other models were displayed on the black boxes with coral represents significance with P-value < 0.05.

Figure 6: Evaluation of statistically significant variants. (**A**) Training and validation performance of the Genotype model across epochs, based on 5-fold cross-validation. (**B**) Estimated heritability of significant SNPs (P-value < 0.05) identified by different models for the LMP trait. (**C**) Genomic prediction accuracy (measured by Pearson correlation coefficient [PCC] using the rrBLUP model), SNPs number and Enrichment of informative SNPs across different SNP sets: I) 11,084 potential causal variants identified by DeepAnnotation (including 10,041 non-coding variants or cis-regulatory elements and 1,043 coding or RNA secondary structure-related variants with P-value < 0.01 from the backtracking strategy). II) 644,964 SNPs located within 1Mb of the 11,084 DeepAnnotation predicted causal variants. III) 4,850 SNPs with GWAS P-value < 1.0e-6. IV) 11,083 SNPs with GWAS P-value < 1.0e-5. V) 24,833 SNPs with GWAS P-value < 1.0e-4. VI) Ten sets of 11,084 SNPs randomly selected across the genome (mean PCC reported).

Additional Files

Supplementary File 1: The algorithm, implementation, and interpretation of DeepAnnotation.

Supplementary Figure S1: Fine mapping through interpretable deep learning model.

Supplementary Figure S2: Average PCC from different sets of SNPs with rrBLUP model.

Supplementary Figure S3: Receiver operating characteristic (ROC) curves of ATAC data learned from DeepSEA model for different tissues of different species.

Supplementary Table S1: PCC scores of different hyperparameters combination based on 2-fold

cross-validation.

Supplementary Table S2: Statistical information of signatures (terms) for significant metaterms.

Supplementary Table S3: Statistical information of genes and non-coding SNPs with significant contributions of LMP via metaterm8.

Supplementary Table S4: Summary information of epigenomic and transcriptomic data.

Reference

1. Hayes BJ, Lewin HA, Goddard ME. The future of livestock breeding: genomic selection for efficiency, reduced emissions intensity, and adaptation. Trends Genet 2013;29:206-214.
2. Yáñez JM, Xu P, Carvalheiro R, et al. Genomics applied to livestock and aquaculture breeding. Evol Appl 2022;15:517-522.
3. Jonas E, de Koning DJ. Does genomic selection have a future in plant breeding? Trends Biotechnol 2013;31:497-504.
4. Wan W, Qin Y, Shi G, et al. Genetic improvement of aquaculture performance for tetraploid Pacific oysters, Crassostrea gigas: a case study of four consecutive generations of selective breeding. Aquaculture 2023;563.
5. Meuwissen TH, Hayes BJ, Goddard ME. Prediction of total genetic value using genome-wide dense marker maps. Genetics 2001;157:1819-1829.
6. Ribaut JM, Ragot M. Marker-assisted selection to improve drought adaptation in maize: the backcross approach, perspectives, limitations, and alternatives. J Exp Bot 2007;58:351-360.
7. Desta ZA, Ortiz R. Genomic selection: genome-wide prediction in plant improvement. Trends Plant Sci 2014;19:592-601.

8. Hu J, Chen B, Zhao J, et al. Genomic selection and genetic architecture of agronomic traits during modern rapeseed breeding. Nat Genet 2022;54:694-704.

9. Georges M, Charlier C, Hayes B. Harnessing genomic information for livestock improvement. Nat Rev Genet 2019;20:135-156.

10. Crossa J, Pérez-Rodríguez P, Cuevas J, et al. Genomic selection in plant breeding: methods, models, and perspectives. Trends Plant Sci 2017;22:961-975.

11. Song H, Dong T, Yan X, et al. Genomic selection and its research progress in aquaculture breeding. Rev Aquac 2022;15:274-291.

12. Hickey JM, Chiurugwi T, Mackay I, et al. Genomic prediction unifies animal and plant breeding programs to form platforms for biological discovery. Nat Genet 2017;49:1297-1303.

13. Jannink JL, Lorenz AJ, Iwata H. Genomic selection in plant breeding: from theory to practice. Brief Funct Genomics 2010;9:166-177.

14. Henderson CR. Estimation of genetic parameters. Ann Math Stat 1950;21:309–310.

15. Thompson R. Estimation of quantitative genetic parameters. Proc Biol Sci 2008;275:679-686.

16. Goddard ME, Hayes BJ. Genomic selection. J Anim Breed Genet 2007;124:323-330.

17. Xu Y, Crouch JH. Marker‐assisted selection in plant breeding: from publications to practice. Crop Science 2008;48:391-407.

18. Endelman JB. Ridge regression and other kernels for genomic selection with R package rrBLUP. Plant Genome 2011;4:250-255.

19. Barry CS, Walker VM, Cheesman R, et al. How to estimate heritability: a guide for genetic epidemiologists. Int J Epidemiol 2023;52: 624-632.

20. Kaler AS, Purcell LC, Beissinger T, et al. Genomic prediction models for traits differing in

heritability for soybean, rice, and maize. BMC Plant Biol 2022;22:87.

21. Clark SA, Hickey JM, Daetwyler HD, et al. The importance of information on relatives for the prediction of genomic breeding values and the implications for the makeup of reference data sets in livestock breeding schemes. Genet Sel Evol 2012;44:4.

22. Luan T, Woolliams JA, Lien S, et al. The accuracy of genomic selection in Norwegian red cattle assessed by cross-validation. Genetics 2009;183:1119-1126.

23. Zhang H, Yin L, Wang M, et al. Factors affecting the accuracy of genomic selection for agricultural economic traits in maize, cattle, and pig populations. Front Genet 2019;10:189.

24. Daetwyler HD, Pong-Wong R, Villanueva B, et al. The impact of genetic architecture on genome-wide evaluation methods. Genetics 2010;185:1021-1031.

25. Breen EJ, MacLeod IM, Ho PN, et al. BayesR3 enables fast MCMC blocked processing for largescale multi-trait genomic prediction and QTN mapping analysis. Commun Biol 2022;5:661.

26. Erbe M, Hayes BJ, Matukumalli LK, et al. Improving accuracy of genomic predictions within and between dairy cattle breeds with imputed high-density single nucleotide polymorphism panels. J Dairy Sci 2012;95:4114-4129.

27. Moser G, Lee SH, Hayes BJ, et al. Simultaneous discovery, estimation and prediction analysis of complex traits using a bayesian mixture model. PLoS Genet 2015;11:e1004969.

28. Mollandin F, Rau A, Croiseau P. An evaluation of the predictive performance and mapping power of the BayesR model for genomic prediction. G3 (Bethesda) 2021;11.

29. MacLeod IM, Bowman PJ, Vander Jagt CJ, et al. Exploiting biological priors and sequence variants enhances QTL discovery and genomic prediction of complex traits. BMC Genomics

2016;17:144.

30. Xiang R, Breen EJ, Prowse-Wilkins CP, et al. Bayesian genome-wide analysis of cattle traits using variants with functional and evolutionary significance. Animal Production Science 2021;61:1818-1827.

31. Xiang R, MacLeod IM, Daetwyler HD, et al. Genome-wide fine-mapping identifies pleiotropic and functional variants that predict many traits across global cattle populations. Nat Commun 2021;12:860.

32. Mollandin F, Gilbert H, Croiseau P, et al. Accounting for overlapping annotations in genomic prediction models of complex traits. BMC Bioinformatics 2022;23:365.

33. Speed D, Balding DJ. MultiBLUP: improved SNP-based prediction for complex traits. Genome Res 2014;24:1550-1557.

34. Yan J, Xu Y, Cheng Q, et al. LightGBM: accelerated genomically designed crop breeding through ensemble learning. Genome Biol 2021;22:271.

35. Howard R, Carriquiry AL, Beavis WD. Parametric and nonparametric statistical methods for genomic selection of traits with additive and epistatic genetic architectures. G3 (Bethesda) 2014;4:1027-1046.

36. Yin L, Zhang H, Zhou X, et al. KAML: improving genomic prediction accuracy of complex traits using machine learning determined parameters. Genome Biol 2020;21:146.

37. Wang K, Abid MA, Rasheed A, et al. DNNGP, a deep neural network-based method for genomic prediction using multi-omics data in plants. Mol Plant 2023;16:279-293.

38. Ma W, Qiu Z, Song J, et al. A deep convolutional neural network approach for predicting phenotypes from genotypes. Planta 2018;248:1307-1318.

39. Weckwerth W, Ghatak A, Bellaire A, et al. PANOMICS meets germplasm.Plant Biotechnol J 2020;18:1507-1525.

40. Montesinos-López OA, Montesinos-López A, Pérez-Rodríguez P, et al. A review of deep learning applications for genomic selection. BMC Genomics 2021;22:19.

41. van Hilten A, Kushner SA, Kayser M, et al. GenNet framework: interpretable deep learning for predicting phenotypes from genetic data. Commun Biol 2021;4:1094.

42. Hanczar B, Zehraoui F, Issa T, et al. Biological interpretation of deep neural network for phenotype prediction based on gene expression. BMC Bioinformatics 2020;21:501.

43. Tanaka H, Kreisberg JF, Ideker T. Genetic dissection of complex traits using hierarchical biological knowledge. PLoS Comput Biol 2021;17:e1009373.

44. Kang M, Ko E, Mersha TB. A roadmap for multi-omics data integration using deep learning. Brief Bioinform 2022;23.

45. Ritchie MD, Holzinger ER, Li R, et al. Methods of integrating data to uncover genotype-phenotype interactions. Nat Rev Genet 2015;16:85-97.

46. Fu Y, Xu J, Tang Z, et al. A gene prioritization method based on a swine multi-omics knowledgebase and a deep learning model. Commun Biol 2020;3:502.

47. Wang D, Liu S, Warrell J, et al. Comprehensive functional genomic resource and integrative model for the human brain. Science 2018;362.

48. Amariuta T, Ishigaki K, Sugishita H, et al. Improving the trans-ancestry portability of polygenic risk scores by prioritizing variants in predicted cell-type-specific regulatory elements. Nat Genet 2020;52:1346-1354.

49. Azodi CB, Pardo J, VanBuren R, et al. Transcriptome-based prediction of complex traits in

maize. Plant Cell 2020;32:139-151.

50. Zhao T, Zeng J, Cheng H. Extend mixed models to multilayer neural networks for genomic prediction including intermediate omics data. Genetics 2022;221:iyac034.

51. Ye S, Li J, Zhang Z. Multi-omics-data-assisted genomic feature markers preselection improves the accuracy of genomic prediction. J Anim Sci Biotechnol 2020;11:109.

52. Elmarakeby HA, Hwang J, Arafeh R, et al. Biologically informed deep neural network for prostate cancer discovery. Nature 2021;598:348-352.

53. Sammut SJ, Crispin-Ortuzar M, Chin SF, et al. Multi-omic machine learning predictor of breast cancer therapy response. Nature 2022;601:623-629.

54. Garg M, Karpinski M, Matelska D, et al. Disease prediction with multi-omics and biomarkers empowers case-control genetic discoveries in the UK Biobank. Nat Genet 2024;56:1821-1831.

55. Hari Dass SA, McCracken K, Pokhvisneva I, et al. A biologically-informed polygenic score identifies endophenotypes and clinical conditions associated with the insulin receptor function on specific brain regions. EBioMedicine 2019;42:188-202.

56. Yengo L, Vedantam S, Marouli E, et al. A saturated map of common genetic variants associated with human height. Nature 2022;610:704-712.

57. Mathieson I. The omnigenic model and polygenic prediction of complex traits. Am J Hum Genet 2021;108:1558-1563.

58. Meuleman W, Muratov A, Rynes E, et al. Index and biological spectrum of human DNase I hypersensitive sites. Nature 2020;584:244-251.

59. Lu C, Zaucha J, Gam R, et al. Hypothesis-free phenotype prediction within a genetics-first framework. Nat Commun 2023;14:919.

60. Fehrmann RS, Karjalainen JM, Krajewska M, et al. Gene expression analysis identifies global gene dosage sensitivity in cancer. Nat Genet 2015;47:115-125.

61. Wang C, Chen C, Lei B, et al. Constructing eRNA-mediated gene regulatory networks to explore the genetic basis of muscle and fat-relevant traits in pigs. Genetics Selection Evolution 2024; 56:28.

62. Jiang Z, Chen C, Xu Z, et al. SIGNET: transcriptome-wide causal inference for gene regulatory networks. Sci Rep 2023;13:19371.

63. Ma W, Chen S, Qi Y, et al. easyMF: a web platform for matrix factorization-based gene discovery from large-scale transcriptome data. Interdiscip Sci 2022;14:746-758.

64. Zhou J, Troyanskaya OG. Predicting effects of noncoding variants with deep learning-based sequence model. Nat Methods 2015;12:931-934.

65. Lorenz R, Bernhart SH, Honer Zu Siederdissen C, et al. ViennaRNA Package 2.0. Algorithms Mol Biol 2011;6:26.

66. Kolde R, Laur S, Adler P, et al. Robust rank aggregation for gene list integration and meta-analysis. Bioinformatics 2012;28:573-580.

67. Zheng M, Xiao S, Guo T, et al. DNA methylomic homogeneity and heterogeneity in muscles and testes throughout pig adulthood. Aging (Albany NY) 2020;12:25412-25431.

68. Yang Y, Fan X, Yan J, et al. A comprehensive epigenome atlas reveals DNA methylation regulating skeletal muscle development. Nucleic Acids Res 2021;49:1313-1329.

69. Gujral P, Mahajan V, Lissaman AC, et al. Histone acetylation and the role of histone deacetylases in normal cyclic endometrium. Reprod Biol Endocrinol 2020;18:84.

70. Tian H, Liu S, Ren J, et al. Role of histone deacetylases in skeletal muscle physiology and

systemic energy homeostasis: implications for metabolic diseases and therapy. Front Physiol 2020;11:949.

71. Rojas A, Aguilar R, Henriquez B, et al. Epigenetic control of the bone-master Runx2 gene during osteoblast-lineage commitment by the histone demethylase JARID1B/KDM5B*. J Biol Chem 2015;290:28329-28342.

72. Li C-Y, Wang W, Leung C-H, et al. KDM5 family as therapeutic targets in breast cancer: pathogenesis and therapeutic opportunities and challenges. Mol Cancer 2024;23:109.

73. Yoo J, Kim GW, Jeon YH, et al. Drawing a line between histone demethylase KDM5A and KDM5B: their roles in development and tumorigenesis. Exp Mol Med 2022;54:2107-2117.

74. Backe MB, Jin C, Andreone L, et al. The lysine demethylase KDM5B regulates islet function and glucose homeostasis. J Diabetes Res 2019;2019:5451038.

75. Turcotte LP, Fisher JS. Skeletal muscle insulin resistance: roles of fatty acid metabolism and exercise. Phys Ther 2008;88:1279-1296.

76. Ayuso M, Fernandez A, Nunez Y, et al. Comparative analysis of muscle transcriptome between pig genotypes identifies genes and regulatory mechanisms associated to growth, fatness and metabolism. PLoS One 2015;10:e0145162.

77. Rusmini P, Polanco MJ, Cristofani R, et al. Aberrant autophagic response in the muscle of a knock-in mouse model of spinal and bulbar muscular atrophy. Sci Rep 2015;5:15174.

78. Corso D, Chemello F, Alessio E, et al. MyoData: an expression knowledgebase at single cell/nucleus level for the discovery of coding-noncoding RNA functional interactions in skeletal muscle. Comput Struct Biotechnol J 2021;19:4142-4155.

79. https://github.com/mawenlong2016/DeepAnnotation. Accessed: 2 January 2024.

80. https://hub.docker.com/r/wenlong2023/deepannotation. Accessed: 2 January 2024.

81. Yang R, Guo X, Zhu D, et al. Accelerated deciphering of the genetic architecture of agricultural economic traits in pigs using a low-coverage whole-genome sequencing strategy. Gigascience 2021;10: giab048.

82. Andersson L, Archibald AL, Bottema CD, et al. Coordinated international action to accelerate genome-to-phenome with FAANG, the Functional Annotation of Animal Genomes project. Genome Biol 2015;16:57.

83. Tian D, Wang P, Tang B, et al. GWAS Atlas: a curated resource of genome-wide variant-trait associations in plants and animals. Nucleic Acids Res 2020;48:D927-D932.

84. Fu Y, Liu H, Dou J, et al. IAnimal: a cross-species omics knowledgebase for animals. Nucleic Acids Res 2023;51:D1312-D1324.

85. Zeng H, Zhang W, Lin Q, et al. PigBiobank: a valuable resource for understanding genetic and biological mechanisms of diverse complex traits in pigs. Nucleic Acids Res 2024;52:D980-D989.

86. Wang C, Zhang J, Veldsman WP, et al. A comprehensive investigation of statistical and machine learning approaches for predicting complex human diseases on genomic variants. Brief Bioinform 2023;24.

87. Wang X, Wen Y. A penalized linear mixed model with generalized method of moments for prediction analysis on high-dimensional multi-omics data. Brief Bioinform 2022;23.

88. Reel PS, Reel S, Pearson E, et al. Using machine learning approaches for multi-omics data analysis: a review. Biotechnol Adv 2021;49:107739.

89. Yang J, Lee SH, Goddard ME, et al. GCTA: a tool for genome-wide complex trait analysis.

Am J Hum Genet 2011;88:76-82.

90. Whittaker JC, Thompson R, Denham MC. Marker-assisted selection using ridge regression. Genet Res 2000;75:249-252.

91. Friedman JH. Greedy function approximation: a gradient boosting machine. Ann Statist 2001;29.

92. Ke G, Meng Q, Finley T, et al. Lightgbm: a highly efficient gradient boosting decision tree. Adv Neural Inf Process Syst 2017;30.

93. Rumelhart DE, Hinton GE, Williams RJ. Learning representations by back-propagating errors. Nature 1986;323:533-536.

94. Lee T, Sung MK, Lee S, et al. Convolutional neural network model to predict causal risk factors that share complex regulatory features. Nucleic Acids Res 2019;47:e146.

95. Browning BL, Zhou Y, Browning SR. A one-penny imputed genome from next-generation reference panels. Am J Hum Genet 2018;103:338-348.

96. Zhao Y, Hou Y, Xu Y, et al. A compendium and comparative epigenomics analysis of cis-regulatory elements in the pig genome. Nat Commun 2021;12:2217.

97. Kern C, Wang Y, Xu X, et al. Functional annotations of three domestic animal genomes provide vital resources for comparative and agricultural research. Nat Commun 2021;12:1821.

98. Quinlan AR, Hall IM. BEDTools: a flexible suite of utilities for comparing genomic features. Bioinformatics 2010;26:841-842.

99. Kim D, Langmead B, Salzberg SL. HISAT: a fast spliced aligner with low memory requirements. Nat Methods 2015;12:357-360.

100. Liao Y, Smyth GK, Shi W. featureCounts: an efficient general purpose program for assigning

sequence reads to genomic features. Bioinformatics 2014;30:923-930.

101. Shameer K, Tripathi LP, Kalari KR, et al. Interpreting functional effects of coding variants: challenges in proteome-scale prediction, annotation and assessment. Brief Bioinform 2016;17:841-862.

102. Rojano E, Seoane P, Ranea JAG, et al. Regulatory variants: from detection to predicting impact. Brief Bioinform 2019;20:1639-1654.

103. Yu MK, Kramer M, Dutkowski J, et al. Translation of genotype to phenotype by a hierarchy of cell subsystems. Cell Syst 2016;2:77-88.

104. Xiang R, Fang L, Liu S, et al. Gene expression and RNA splicing explain large proportions of the heritability for complex traits in cattle. Cell Genom 2023;3:100385.

105. Lee T, Yang S, Kim E, et al. AraNet v2: an improved database of co-functional gene networks for the study of *Arabidopsis thaliana* and 27 other nonmodel plant species. Nucleic Acids Res 2015;43:D996-1002.

106. Wadkins RM. Targeting DNA secondary structures. Curr Med Chem 2000;7:1-15.

107. González-Camacho JM, Ornella L, Pérez-Rodríguez P et al. Applications of machine learning methods to genomic selection in breeding wheat for rust resistance. Plant Genome 2018;11.

108. https://doi.org/10.48546/workflowhub.workflow.1732.1. Accessed: 15 June 2025.

109. Ma W, Zheng W, Qin S, et al. DeepAnnotation: a novel interpretable deep learning-based genomic selection model that integrates comprehensive functional annotations [Data set]. Zenodo. https://doi.org/10.5281/zenodo.8410693.

110. Ma W, Zheng W, Qin S, et al. DeepAnnotation: a novel interpretable deep learning-based genomic selection model that integrates comprehensive functional annotations [DOME-ML

Annotations]. DOME-ML Registry, https://registry.dome-ml.org/review/fgsm6yazie.

111. https://doi.org/10.5524/100894. Accessed: 24 September 2021.

112. https://www.ncbi.nlm.nih.gov/geo/query/acc.cgi?acc=GSE143288. Accessed: 26 April 2021.

113. https://www.ncbi.nlm.nih.gov/geo/query/acc.cgi?acc=GSE158414. Accessed: 13 May 2024.

114. http://iswine.iomics.pro/. Accessed: 20 February 2022.

115. https://www.ncbi.nlm.nih.gov/sra. Accessed: 22 February 2022.

116. http://ftp.ensembl.org/pub/release-105/gtf/sus_scrofa/Sus_scrofa.Sscrofa11.1.105.gtf.gz. Accessed: 1 March 2022.

117. http://useast.ensembl.org/biomart/martview/05b5f89cff700cd5d5c2e0455be125a5. Accessed: 7 April 2022.

118.https://www.genome.jp/kegg-bin/download_htext?htext=ssc00001.keg&format=htext&filedir=. Accessed: 11 April 2022.

119. https://doi.org/10.5281/zenodo.15661212. Accessed: 15 June 2025.

120.https://github.com/mawenlong2016/DeepAnnotation/blob/main/code_annotations/Script_for_building_the_comprehensive_functional_annotations.sh. Accessed: 22 Mar 2025.

Figure 1

Click here to access/download;Figure;Figure 1.pdf

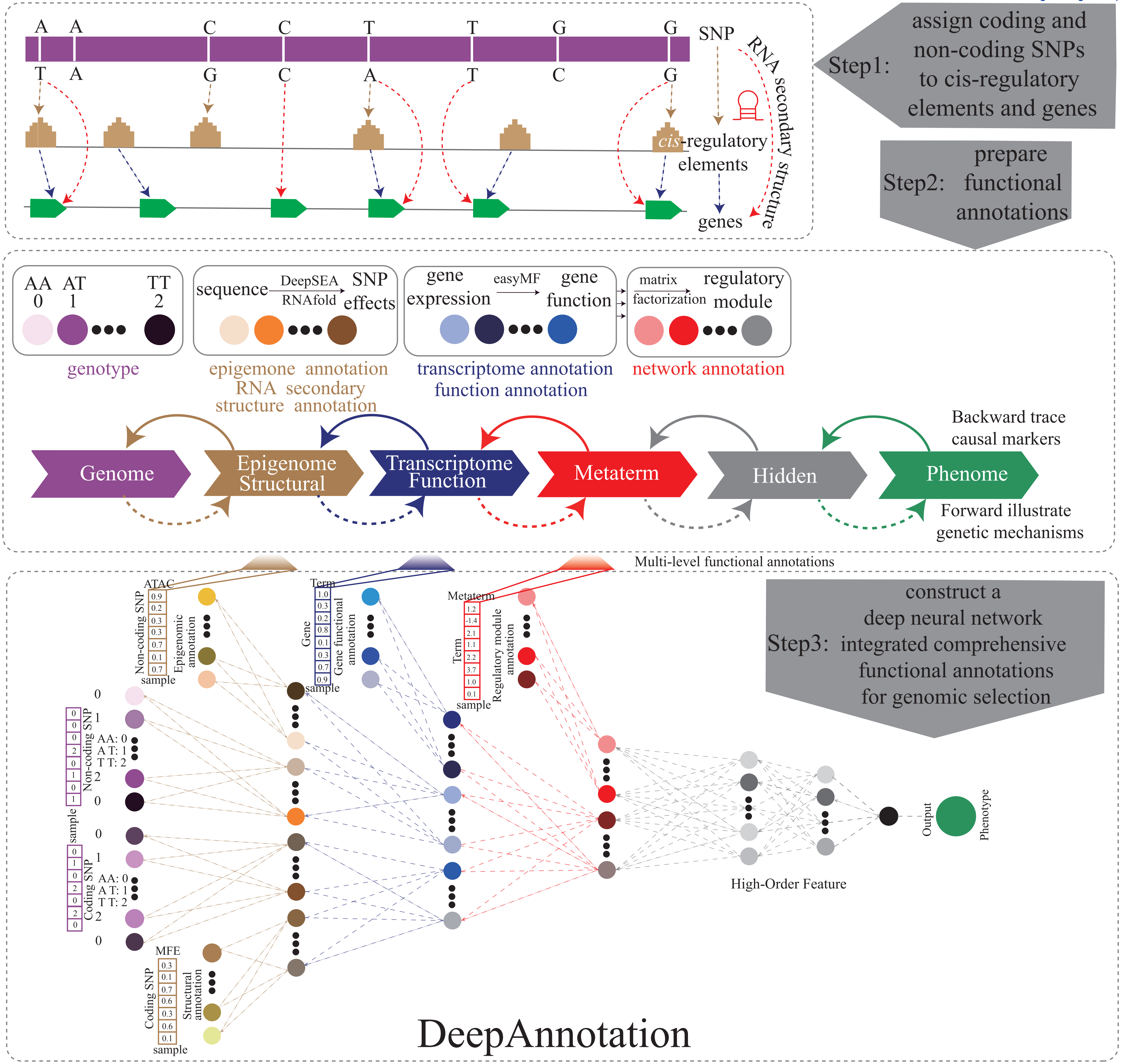

Figure 2

Click here to access/download;Figure;Figure 2.pdf

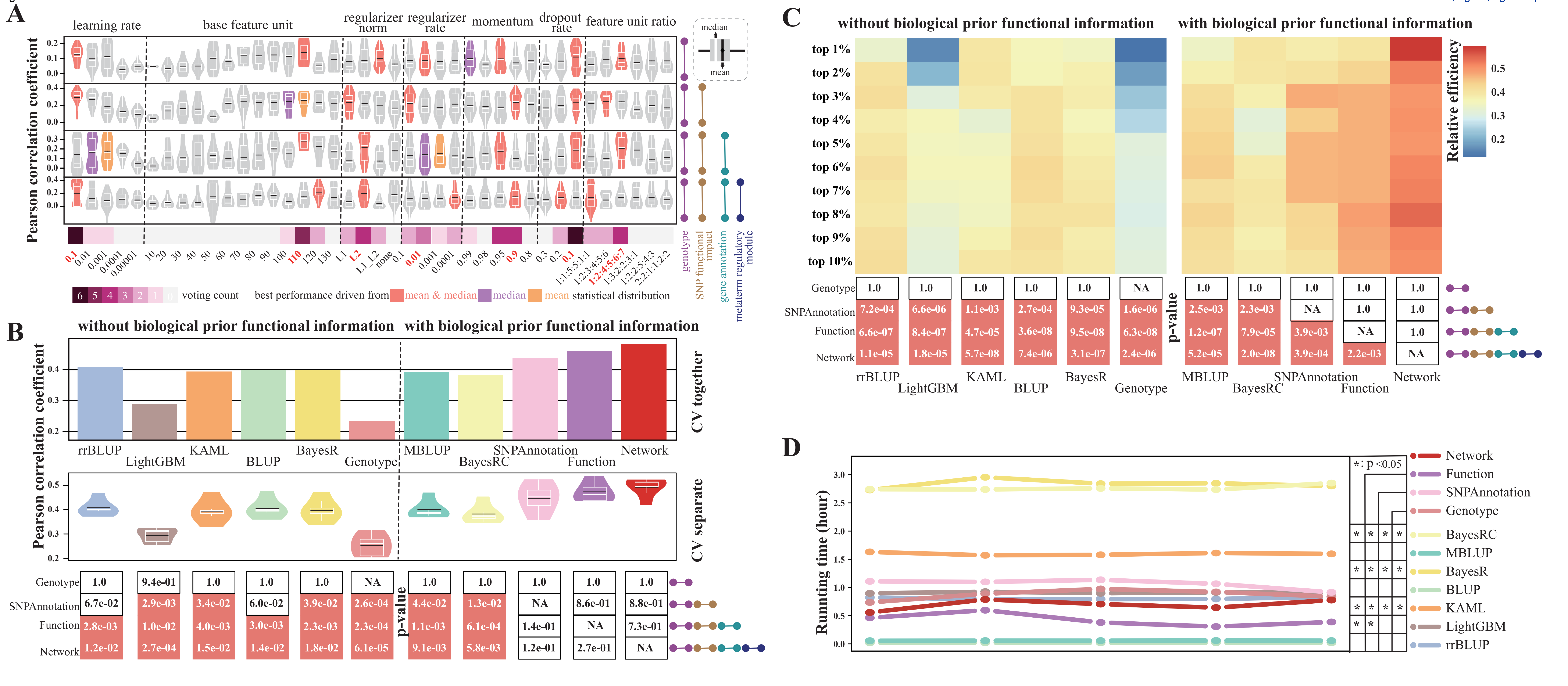

Figure 3

Click here to access/download;Figure;Figure 3.pdf

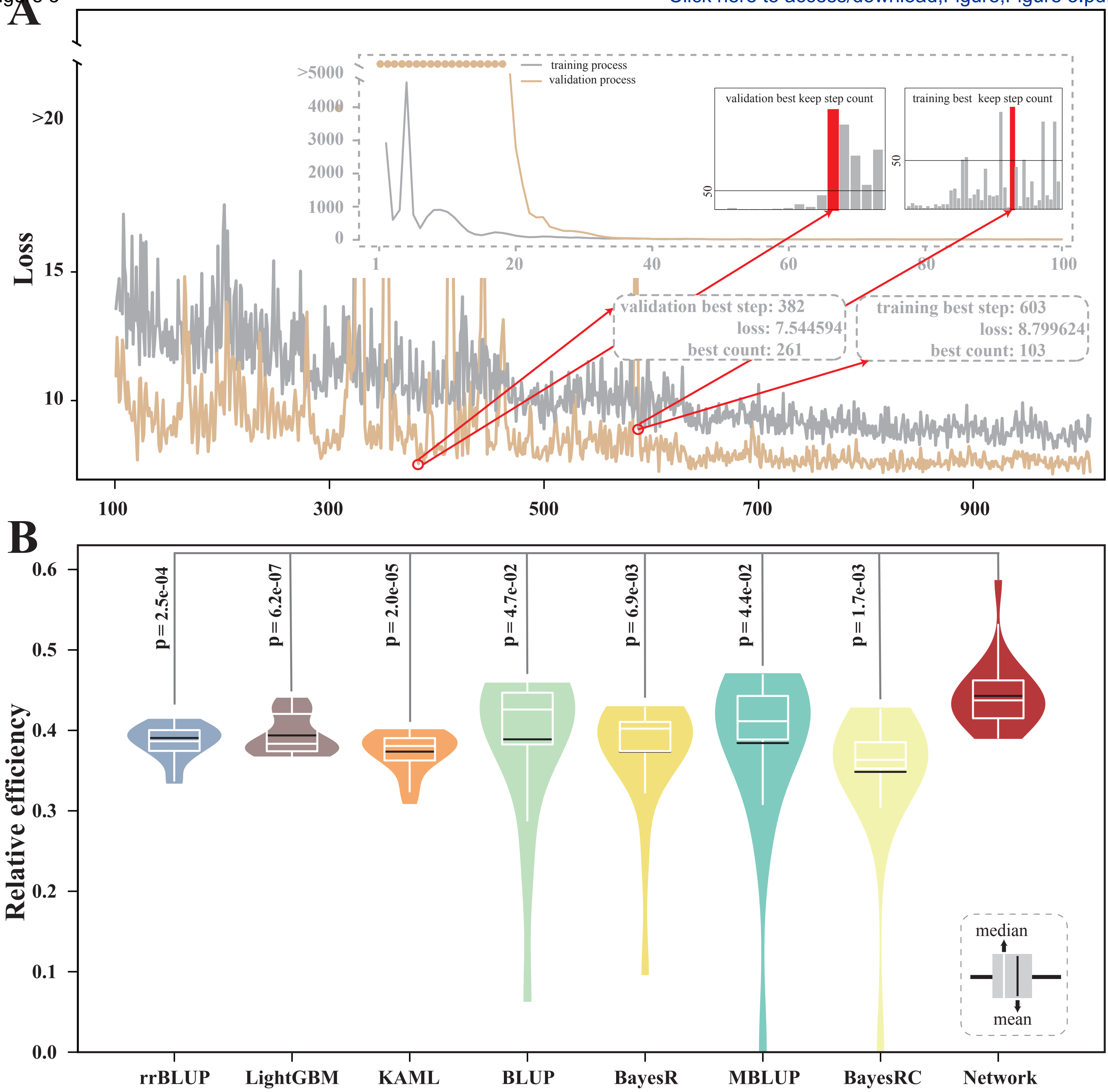

Figure 4

Click here to access/download;Figure;Figure 4.pdf

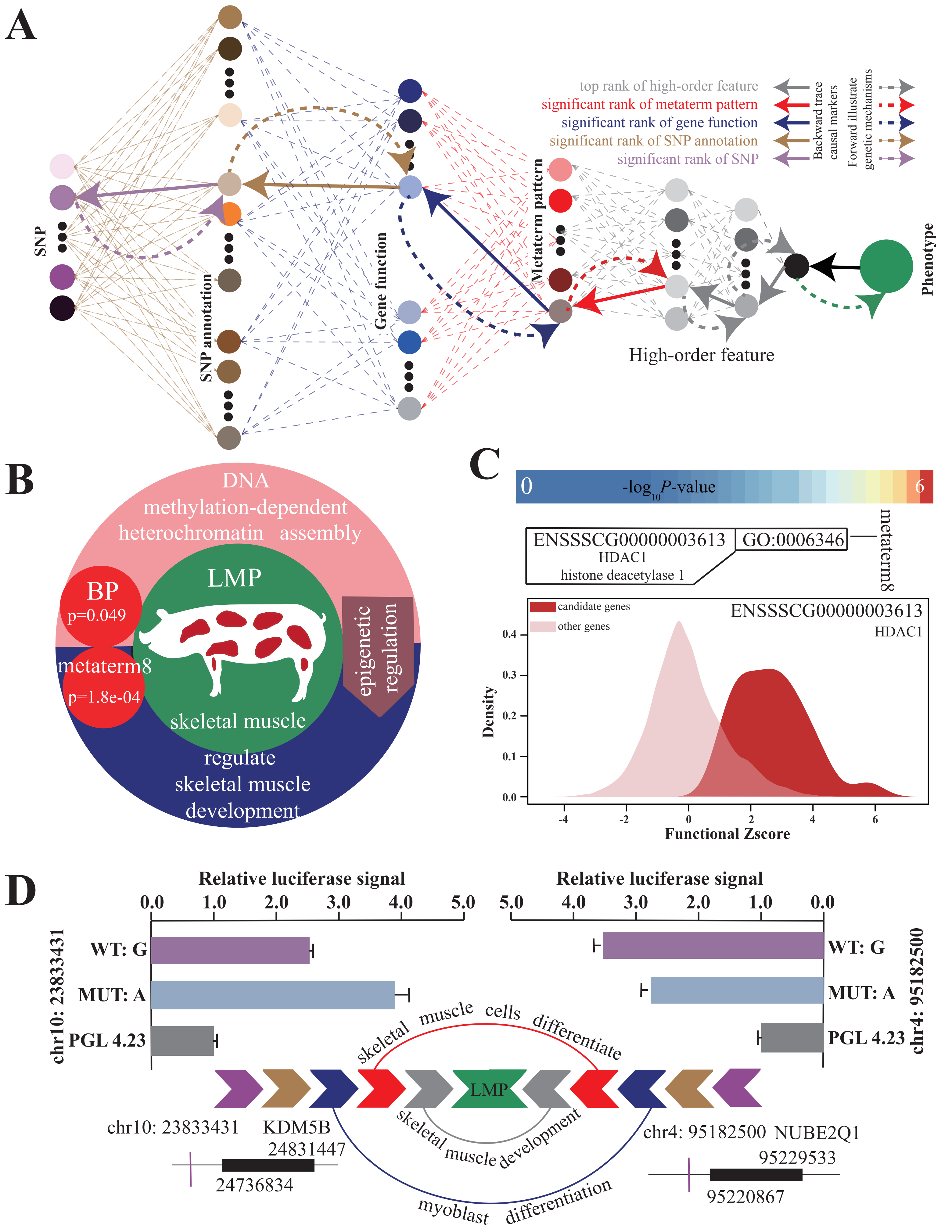

Figure 5

Click here to access/download;Figure;Figure 5.pdf

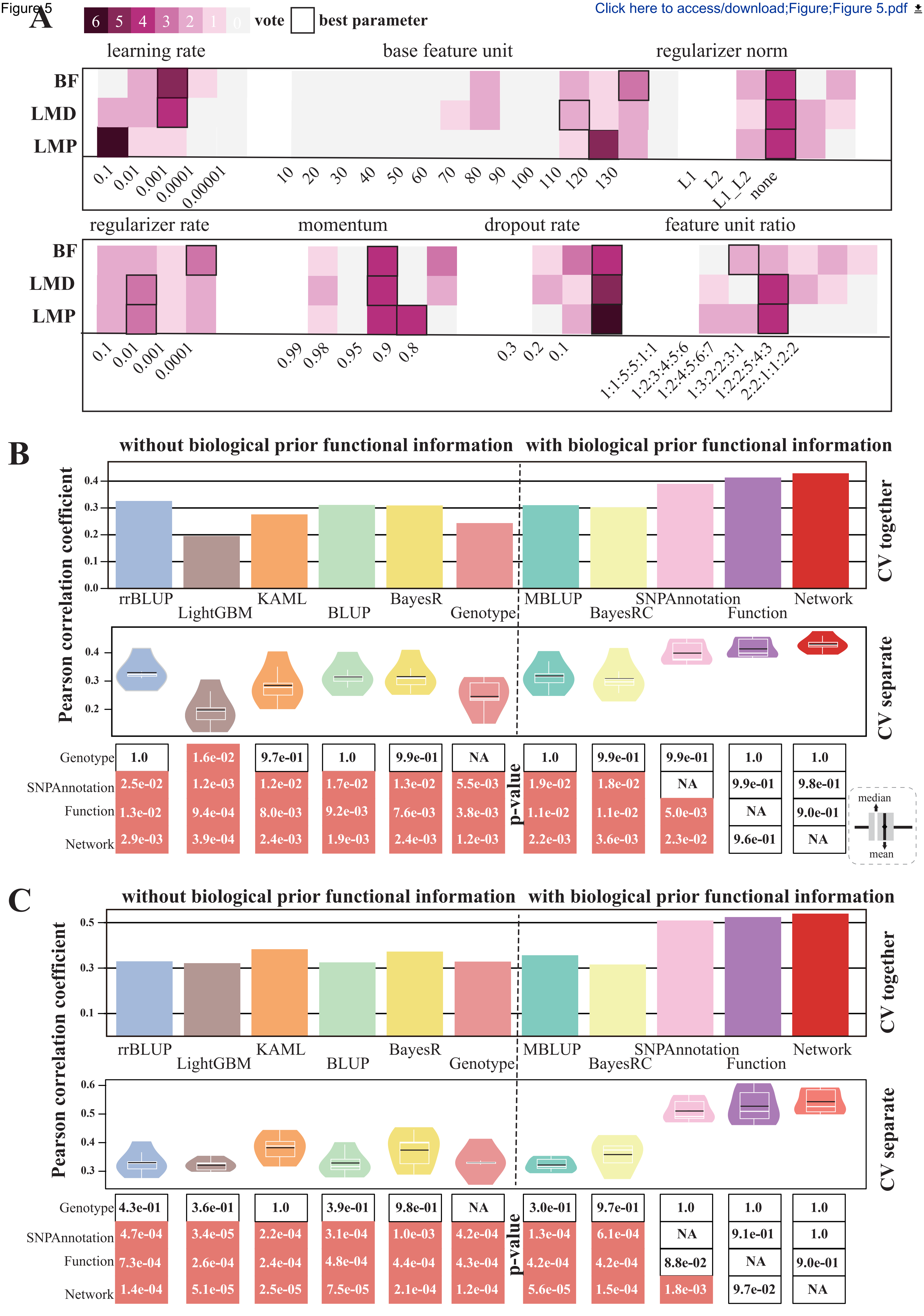

B p-value

	rrBLUP	LightGBM	KAML	BLUP	BayesR	Genotype	MBLUP	BayesRC	SNPAnnotation	Function	Network
Genotype	1.0	1.6e-02	9.7e-01	1.0	9.9e-01	NA	1.0	9.9e-01	9.9e-01	1.0	1.0
SNPAnnotation	2.5e-02	1.2e-03	1.2e-02	1.7e-02	1.3e-02	5.5e-03	1.9e-02	1.8e-02	NA	9.9e-01	9.8e-01
Function	1.3e-02	9.4e-04	8.0e-03	9.2e-03	7.6e-03	3.8e-03	1.1e-02	1.1e-02	5.0e-03	NA	9.0e-01
Network	2.9e-03	3.9e-04	2.4e-03	1.9e-03	2.4e-03	1.2e-03	2.2e-03	3.6e-03	2.3e-02	9.6e-01	NA

C p-value

	rrBLUP	LightGBM	KAML	BLUP	BayesR	Genotype	MBLUP	BayesRC	SNPAnnotation	Function	Network
Genotype	4.3e-01	3.6e-01	1.0	3.9e-01	9.8e-01	NA	3.0e-01	9.7e-01	1.0	1.0	1.0
SNPAnnotation	4.7e-04	3.4e-05	2.2e-04	3.1e-04	1.0e-03	4.2e-04	1.3e-04	6.1e-04	NA	9.1e-01	1.0
Function	7.3e-04	2.6e-04	2.4e-04	4.8e-04	4.4e-04	4.3e-04	4.2e-04	4.2e-04	8.8e-02	NA	9.0e-01
Network	1.4e-04	5.1e-05	2.5e-05	7.5e-05	2.1e-04	1.2e-04	5.6e-05	1.5e-04	1.8e-03	9.7e-02	NA

Figure 6

Click here to access/download;Figure;Figure 6.pdf

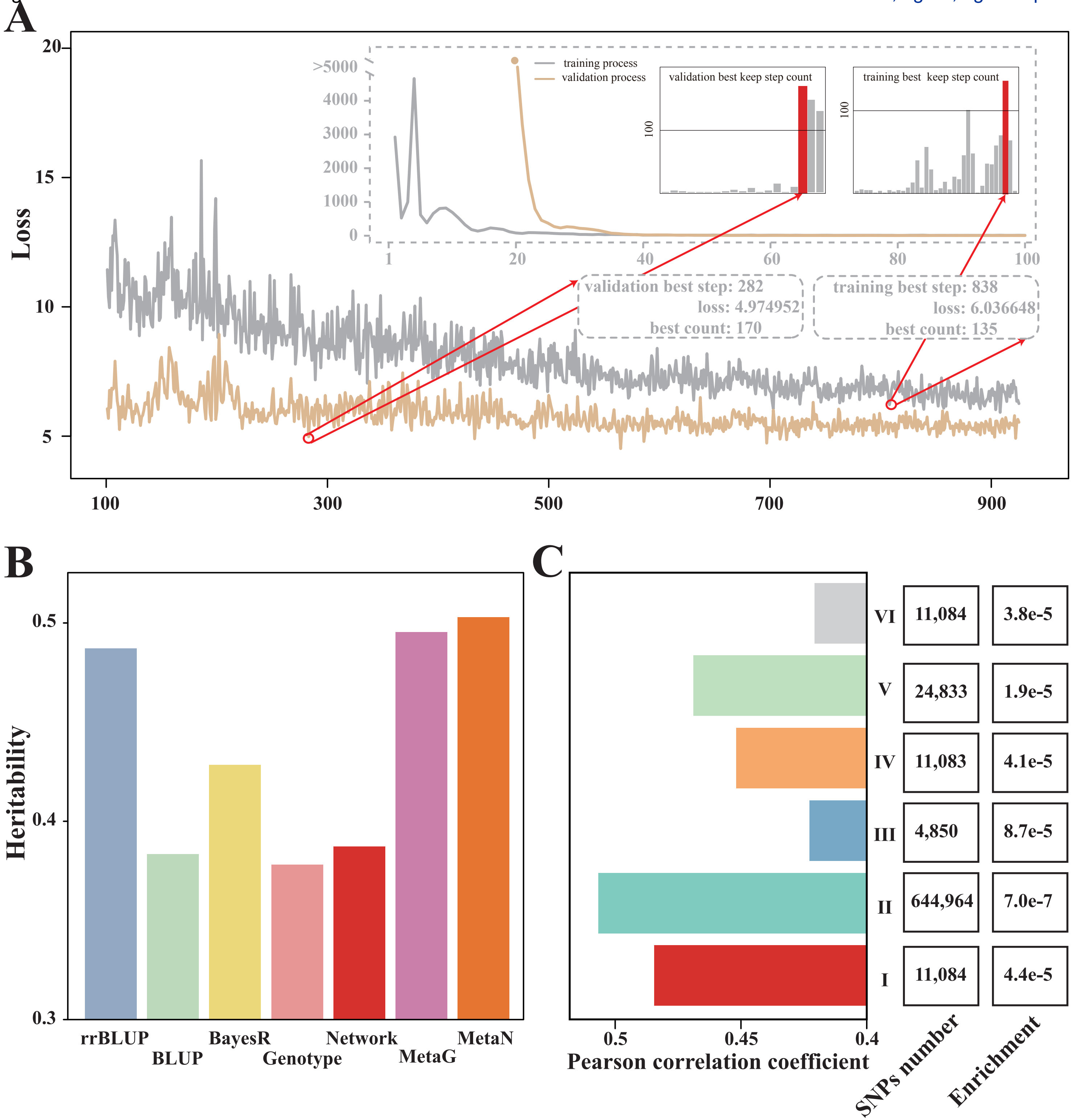

Supplementary Figure S1

Click here to access/download
Supplementary Material
Supplementary Figure S1.pdf

Supplementary Figure S2

Click here to access/download
Supplementary Material
Supplementary Figure S2.pdf

Supplementary Figure S3

Click here to access/download

Supplementary Material

Supplementary Figure S3.pdf

Supplementary Table S1

Click here to access/download
Supplementary Material
Supplementary Table S1.xlsx

Supplementary Table S2

Click here to access/download
Supplementary Material
Supplementary Table S2.xlsx

Supplementary Table S3

Click here to access/download
Supplementary Material
Supplementary Table S3.xlsx

Supplementary Table S4

Click here to access/download
Supplementary Material
Supplementary Table S4.xlsx

Supplementary File 1

Click here to access/download
Supplementary Material
Supplementary File 1.docx

Click here to access/download
Supplementary Material
point-by-point response to reviewers comments.docx
