# Supplementary material for: DeepAnnotation: A novel interpretable deep learning–based genomic selection model that integrates comprehensive functional annotations
Source: Gigascience. 2025 Aug 28;14:giaf083. doi: 10.1093/gigascience/giaf083 (PMC12392413; doi:10.1093/gigascience/giaf083)
Supplement: giaf083_Authors_Response_To_Reviewer_Comments_original_submission [file giaf083_authors_response_to_reviewer_comments_original_submission.pdf]

Author's Response To Reviewer Comments

We would like to express our sincere gratitude to the editor and the reviewers for their constructive and insightful comments. Their feedback has been invaluable in improving the quality of our manuscript. All authors have carefully discussed each comment and have diligently revised the manuscript to meet the high standards of your esteemed journal.

Following the editor's comments, we have registered DeepAnnotation in bio.tools (biotoolsID: deepannotation) and SciCrunch.org (RRID: SCR_026630). Additionally, all previous comments from the editor have been incorporated into the revised manuscript.

Since our responses to the reviewers' comments include figures and mathematical formulas, we have provided the point-by-point responses as supplementary materials (attached as point-by-point response to reviewers' comments.docx). We kindly request the reviewers to review our revisions by referring to this attachment.
