# Supplementary material for: DeepAnnotation: A novel interpretable deep learning–based genomic selection model that integrates comprehensive functional annotations
Source: Gigascience. 2025 Aug 28;14:giaf083. doi: 10.1093/gigascience/giaf083 (PMC12392413; doi:10.1093/gigascience/giaf083)
Supplement: giaf083_Reviewer_1_Report_Original_Submission — Felix Heinrich -- 2/24/2025 [file giaf083_reviewer_1_report_original_submission.pdf]

Reviewer Report

Title: DeepAnnotation: A novel interpretable deep learning-based genomic selection model that integrates comprehensive functional annotations

Version: Original Submission **Date:** 2/24/2025

Reviewer name: Felix Heinrich

Reviewer Comments to Author:

With this revised version, the authors have addressed the major issues I raised in my previous review. However, there are still concerns regarding the evaluation of predictive performance.In Figures 2 and 3, as well as the corresponding text, the authors use the Pearson correlation coefficient (PCC) of top-ranked samples for evaluation. It is commendable that the authors analyze the ability of the models to identify the highest-ranking individualsâ€"an aspect too often neglected in genomic prediction. However, I do not understand how this measure is calculated, making it difficult to assess whether it is appropriate.I am familiar with two established methods for evaluating a model's ability to correctly select the highest-ranking individuals:- Relative efficiency ("Applications of Machine Learning Methods to Genomic Selection in Breeding Wheat for Rust Resistance"; doi: 10.3835/plantgenome2017.11.0104)-Normalized discounted cumulative gain ("A Ranking Approach to Genomic Selection"; doi: 10.1371/journal.pone.0128570)Both methods compare the sum of the obtained phenotypes when selecting individuals based on model predictions versus selection based on true phenotypes.Regarding the PCC, if, for example, the authors take the top 10% of individuals with the highest predicted phenotype and calculate the PCC between their true and predicted phenotypes, this would not indicate whether the correct individuals were selected. Therefore, I ask the authors to clarify their methodology or replace it with a more suitable alternative.Additionally, I do not understand what is meant by "Spatial PCC values" in Line 804. Furthermore, why and how do the authors normalize PCC values in Figure 2C? PCC is already a normalized measure of covariance, ranging from [-1,1]. The applied normalization alters this range, making interpretation more difficult.Lastly, the authors claim that DeepAnnotation outperforms established methods in both prediction accuracy and computational efficiency. However, the training time shown in Figure 2D does not account for hyperparameter optimization, which, according to the authors' response, required 17 daysâ€"a substantial timespan. The authors themselves acknowledge in Line 562 that the model's performance depends on hyperparameter optimization and further demonstrate that optimal hyperparameters vary across datasets with different phenotypes. Therefore, when comparing methods, it is essential to acknowledge that the reported training time for DeepAnnotation does not include hyperparameter optimization. If this time were accounted for, DeepAnnotation would be the slowest method.The remaining points are line-specific and include questions that need to be clarified and points that may need to be added to or corrected in the manuscript as well as minor spelling mistakes.Line 74: "BULP-based" should likely be "BLUP-based"Line 85: "computational efficient" -> "computational efficiency"Line 162: Missing "the" before "of DeepAnnotation"Line 226: "MBULUP" should be "MBLUP."Line 283: "appeared to be as a" -> likely missing the word "acting" after "be"Line 283: The adjusted P-value is reported as "1.8e-04," whereas in

Figure 4B, it is "p=1.3e-12." If this discrepancy arises because the figure presents an unadjusted P-value, it does not match the P-value of the BP term, which is identical in Line 285 and Figure 4B.Line 305: The text states that there are only two significant non-coding SNPs, but Supplementary Table S3 lists three. SNP chr4_95309469 is not mentioned in the text.Line 326: Missing "in" between "role" and "UBE2Q1"Line 377: "gapes" should be "gaps"Line 416: Missing period between "traits" and "This"Line 458: According to the rrBLUP documentation, lambda should be defined as sigmaÂ²e/sigmaÂ²u (see page 10 of https://cran.r-project.org/web/packages/rrBLUP/rrBLUP.pdf).Line 466: Period at the end of the line instead of a comma.Line 480: Do you mean "BayesR" instead of "BLUP"?Line 497: "different" should be "difference"Line 537: "p values" is inconsistently formatted; elsewhere, "P-value" is used.Line 550: Missing comma between "markers" and "x"Line 617: Missing period after "NCBI"Line 623: Missing period after "server"Line 633: Missing "are" between "that" and "within"Line 648: "In comprehensively" -> "In order to comprehensively"Line 694: "distant" should be "distinct"Lines 803, 806, 833: The color described as "pink" would be more accurately labeled as "coral" or simply "red"Supplementary File 1:Line 45: "wights" should be "weights"Line 91: "phenome" should be "phenotype"Figure 2B: Some P-values are bolded while others are not. For consistency, all P-values should follow the same formatting. Compare, for example, the values for KAML-SNPAnnotation and KAML-Function.

Level of Interest

Please indicate how interesting you found the manuscript: Choose an item.

Quality of Written English

Please indicate the quality of language in the manuscript: Choose an item.

Declaration of Competing Interests

Please complete a declaration of competing interests, considering the following questions:

- Have you in the past five years received reimbursements, fees, funding, or salary from an organisation that may in any way gain or lose financially from the publication of this manuscript, either now or in the future?
- Do you hold any stocks or shares in an organisation that may in any way gain or lose financially from the publication of this manuscript, either now or in the future?
- Do you hold or are you currently applying for any patents relating to the content of the manuscript?
- Have you received reimbursements, fees, funding, or salary from an organization that holds or has applied for patents relating to the content of the manuscript?
- Do you have any other financial competing interests?
- Do you have any non-financial competing interests in relation to this paper?

If you can answer no to all of the above, write 'I declare that I have no competing interests' below. If your reply is yes to any, please give details below.

I declare that I have no competing interests

I agree to the open peer review policy of the journal. I understand that my name will be included on my report to the authors and, if the manuscript is accepted for publication, my named report including any attachments I upload will be posted on the website along with the authors' responses. I agree for my report to be made available under an Open Access Creative Commons CC-BY license (http://creativecommons.org/licenses/by/4.0/). I understand that any comments which I do not wish to be included in my named report can be included as confidential comments to the editors, which will not be published.

Choose an item.

To further support our reviewers, we have joined with Publons, where you can gain additional credit to further highlight your hard work (see: https://publons.com/journal/530/gigascience). On publication of this paper, your review will be automatically added to Publons, you can then choose whether or not to claim your Publons credit. I understand this statement.

Yes Choose an item.
