# Supplementary material for: DeepAnnotation: A novel interpretable deep learning–based genomic selection model that integrates comprehensive functional annotations
Source: Gigascience. 2025 Aug 28;14:giaf083. doi: 10.1093/gigascience/giaf083 (PMC12392413; doi:10.1093/gigascience/giaf083)
Supplement: giaf083_Reviewer_1_Report_Revision_1 — Felix Heinrich -- 3/25/2025 [file giaf083_reviewer_1_report_revision_1.pdf]

Reviewer Report

Title: DeepAnnotation: A novel interpretable deep learning-based genomic selection model that integrates comprehensive functional annotations

Version: Revision 1 **Date:** 3/25/2025

Reviewer name: Felix Heinrich

Reviewer Comments to Author:

I appreciate the authors' revisions and their detailed explanations, particularly regarding their calculation of PCC.However, the current PCC-based evaluation does not seem suitable for assessing the success of genomic selection, as it does not account for whether the correct individuals are selected. The authors' PCC measure only evaluates whether the true top-ranked individuals are predicted in the correct order, but it does not indicate whether these individuals are also predicted as top ranks and thus selected for breeding.To illustrate this issue, I have uploaded a small example comparing PCC and RE (https://github.com/FelixHeinrich/PCC_Example/blob/main/PCC_Example.R).Since the authors have already calculated RE values as part of their response to the review, I strongly recommend replacing PCC with RE for evaluating top-ranked individuals and revising the manuscript accordingly.Minor correctionsLine 311: "predicted to be reside" -> "predicted to reside"Line 458: "serval limitations" -> "several limitations"Line 468: "directly" should likely be "detected"

Level of Interest

Please indicate how interesting you found the manuscript: Choose an item.

Quality of Written English

Please indicate the quality of language in the manuscript: Choose an item.

Declaration of Competing Interests

Please complete a declaration of competing interests, considering the following questions:

- Have you in the past five years received reimbursements, fees, funding, or salary from an organisation that may in any way gain or lose financially from the publication of this manuscript, either now or in the future?
- Do you hold any stocks or shares in an organisation that may in any way gain or lose financially from the publication of this manuscript, either now or in the future?
- Do you hold or are you currently applying for any patents relating to the content of the manuscript?
- Have you received reimbursements, fees, funding, or salary from an organization that holds or has applied for patents relating to the content of the manuscript?

- Do you have any other financial competing interests?
- Do you have any non-financial competing interests in relation to this paper?

If you can answer no to all of the above, write 'I declare that I have no competing interests' below. If your reply is yes to any, please give details below.

I declare that I have no competing interests

I agree to the open peer review policy of the journal. I understand that my name will be included on my report to the authors and, if the manuscript is accepted for publication, my named report including any attachments I upload will be posted on the website along with the authors' responses. I agree for my report to be made available under an Open Access Creative Commons CC-BY license (http://creativecommons.org/licenses/by/4.0/). I understand that any comments which I do not wish to be included in my named report can be included as confidential comments to the editors, which will not be published.

Choose an item.

To further support our reviewers, we have joined with Publons, where you can gain additional credit to further highlight your hard work (see: https://publons.com/journal/530/gigascience). On publication of this paper, your review will be automatically added to Publons, you can then choose whether or not to claim your Publons credit. I understand this statement.

Yes Choose an item.
