# Supplementary material for: DeepAnnotation: A novel interpretable deep learning–based genomic selection model that integrates comprehensive functional annotations
Source: Gigascience. 2025 Aug 28;14:giaf083. doi: 10.1093/gigascience/giaf083 (PMC12392413; doi:10.1093/gigascience/giaf083)
Supplement: giaf083_Reviewer_2_Report_Original_Submission — Ruidong Xiang -- 2/27/2025 [file giaf083_reviewer_2_report_original_submission.pdf]

Reviewer Report

Title: DeepAnnotation: A novel interpretable deep learning-based genomic selection model that integrates comprehensive functional annotations

Version: Original Submission **Date:** 2/27/2025

Reviewer name: Ruidong Xiang

Reviewer Comments to Author:

I thank the authors for spending time and efforts revising the manuscript. The manuscript is significantly improved. I have the following comments:DeepAnnotation uses several other resources to predict variant functional impact, such as NAfold, DeepSEA,easyMF. While the authors provide instructions for DeepAnnotation, can they also provide more detailed instructions for how to train and analysis data using RNAfold, DeepSEA,easyMF for other domestic species. It would be good to have a Github tutorial and code as well to show how to use these models for non-human and non-pig species.Can the authors report the estimates of heritability and genetic correlation of analysed pig traits?One comprehensive tests of models are conducted, the data used for genomic prediction in the current study is small. This should be discussed as a limitation. Also, please describe the data size analysed in the abstract to include: number of individuals, traits and SNPsAnother limitation is lacking of external validation which uses independent population as validation population - only internal cross validation is used in the current study. This should also be discussed and mentioned in the abstract.Causal SNP: how does the model overcome LD which are higher in livestock? Wet-lab validation is cool but it does not demonstrate the effects on phenotype? For the analysed pig traits, it would be good to show a manhattan plot of GWAS results (say using linear mixed model) then compare this with the fine-mapping of DeepAnnotationIn the response letter, the QTL detection rates are very low in all models, why is that?

Level of Interest

Please indicate how interesting you found the manuscript: Choose an item.

Quality of Written English

Please indicate the quality of language in the manuscript: Choose an item.

Declaration of Competing Interests

Please complete a declaration of competing interests, considering the following questions:

- Have you in the past five years received reimbursements, fees, funding, or salary from an organisation that may in any way gain or lose financially from the publication of this manuscript, either now or in the future?

- Do you hold any stocks or shares in an organisation that may in any way gain or lose financially from the publication of this manuscript, either now or in the future?
- Do you hold or are you currently applying for any patents relating to the content of the manuscript?
- Have you received reimbursements, fees, funding, or salary from an organization that holds or has applied for patents relating to the content of the manuscript?
- Do you have any other financial competing interests?
- Do you have any non-financial competing interests in relation to this paper?

If you can answer no to all of the above, write 'I declare that I have no competing interests' below. If your reply is yes to any, please give details below.

I declare that I have no competing interests.

I agree to the open peer review policy of the journal. I understand that my name will be included on my report to the authors and, if the manuscript is accepted for publication, my named report including any attachments I upload will be posted on the website along with the authors' responses. I agree for my report to be made available under an Open Access Creative Commons CC-BY license (http://creativecommons.org/licenses/by/4.0/). I understand that any comments which I do not wish to be included in my named report can be included as confidential comments to the editors, which will not be published.

Choose an item.

To further support our reviewers, we have joined with Publons, where you can gain additional credit to further highlight your hard work (see: https://publons.com/journal/530/gigascience). On publication of this paper, your review will be automatically added to Publons, you can then choose whether or not to claim your Publons credit. I understand this statement.

Yes Choose an item.
