# Supplementary material for: DeepAnnotation: A novel interpretable deep learning–based genomic selection model that integrates comprehensive functional annotations
Source: Gigascience. 2025 Aug 28;14:giaf083. doi: 10.1093/gigascience/giaf083 (PMC12392413; doi:10.1093/gigascience/giaf083)
Supplement: giaf083_Reviewer_2_Report_Revision_1 — Ruidong Xiang -- 4/21/2025 [file giaf083_reviewer_2_report_revision_1.pdf]

Reviewer Report

Title: DeepAnnotation: A novel interpretable deep learning-based genomic selection model that integrates comprehensive functional annotations

Version: Revision 1 **Date:** 4/21/2025

Reviewer name: Ruidong Xiang

Reviewer Comments to Author:

The authors addressed the majority of my past comments. However, I still have concerns regarding the method and results of detecting causal mutations. First, the detection rate of causal mutations using simulations is all very low. It makes me wonder if there are errors in simulations and/or analyses.Second, in Figure 6 F, as the authors stated, "Red dots highlight potential causal SNPs (P-value < 0.01)". But if one looks at Figure 6F, all the red dots (DeepAnnotation identified causal variants) have higher p-values (insignificant) compared to other SNPs. In other words, DeepAnnotation identified causal variants are much less significant than other SNPs in GWAS. This is not very convincing. Can authors check these results? One way to validate this result is to test the genomic prediction accuracy of DeepAnnotation-identified causal variants. This can be compared with the same number of SNPs, but: 1) has a distance within 1Mb to DeepAnnotation-identified causal variants 2) with a much lower p-value than DeepAnnotation-identified causal variants; the prediction accuracy of DeepAnnotation-identified causal variants should also be compared with a set of random variants selected across the genome with the same number of SNPs to DeepAnnotation-identified causal variants.If the above results are not convincing within this revision, I suggest the authors remove the results of identifying causal mutations from the current manuscript and maybe consider this work as something to be refined in the future. The other results are enough for this paper to my understanding.

Level of Interest

Please indicate how interesting you found the manuscript: Choose an item.

Quality of Written English

Please indicate the quality of language in the manuscript: Choose an item.

Declaration of Competing Interests

Please complete a declaration of competing interests, considering the following questions:

- Have you in the past five years received reimbursements, fees, funding, or salary from an organisation that may in any way gain or lose financially from the publication of this manuscript, either now or in the future?

- Do you hold any stocks or shares in an organisation that may in any way gain or lose financially from the publication of this manuscript, either now or in the future?
- Do you hold or are you currently applying for any patents relating to the content of the manuscript?
- Have you received reimbursements, fees, funding, or salary from an organization that holds or has applied for patents relating to the content of the manuscript?
- Do you have any other financial competing interests?
- Do you have any non-financial competing interests in relation to this paper?

If you can answer no to all of the above, write 'I declare that I have no competing interests' below. If your reply is yes to any, please give details below.

I declare that I have no competing interests

I agree to the open peer review policy of the journal. I understand that my name will be included on my report to the authors and, if the manuscript is accepted for publication, my named report including any attachments I upload will be posted on the website along with the authors' responses. I agree for my report to be made available under an Open Access Creative Commons CC-BY license (http://creativecommons.org/licenses/by/4.0/). I understand that any comments which I do not wish to be included in my named report can be included as confidential comments to the editors, which will not be published.

Choose an item.

To further support our reviewers, we have joined with Publons, where you can gain additional credit to further highlight your hard work (see: https://publons.com/journal/530/gigascience). On publication of this paper, your review will be automatically added to Publons, you can then choose whether or not to claim your Publons credit. I understand this statement.

Yes Choose an item.
